# Tooth Function of the Northern Raccoon (*Procyon lotor*) and Adaptations to Omnivory in the Order Carnivora

**DOI:** 10.1002/jmor.70142

**Published:** 2026-07-03

**Authors:** Sophie E. Koomen, Andreas J. Lang, Thomas Martin

**Affiliations:** ^1^ Abteilung Paläontologie, Bonner Institut für Organismische Biologie Rheinische Friedrich‐Wilhelms‐Universität Bonn Bonn Germany

**Keywords:** dentine exposure, hypocone, metaconule, OFA, wear facets

## Abstract

The raccoon (*Procyon lotor*) is an omnivorous carnivoran, with a diet that consists of plant material such as acorns, corn, fruits and berries, as well as animal matter including insects, crustaceans and vertebrates. While the morphology of the teeth of *P. lotor* is well known, the occlusal functions of crown features of the cheek dentition have not been studied in detail. The aim of this study is to describe the tooth function of the raccoon. Of the 26 raccoon skulls studied, 19 were used in a wear facet analysis and seven were included in a dentine exposure series. Additionally, an Occlusal Fingerprint Analyser (OFA) analysis was performed. The results showed that the power stroke of *P. lotor* consists of two phases, both with a large horizontal component. This suggests that grinding plays a major role in its tooth function, which is corroborated by the presence of grinding facets on the buccal sides of the P4 hypocone, M1 protocone and metaconule and M2 protocone, as well as on the antagonistic surfaces on the lower molars. Abrasion on multiple cusps of the upper and lower cheek teeth and their antagonistic basins shows that crushing is also an important component of raccoon tooth function, and crushing basins exist on the lower as well as upper teeth. The OFA analysis furthermore revealed an occlusal relationship between the enlarged M1 metaconule and the trigonid basin of the m2, while the hypocone of the M1 is shifted lingually with respect to that of the P4, indicating that the M1 metaconule replaces the function of the hypocone. The observed functional replacement of the hypocone by the metaconule on the M1 has not been recorded previously in Carnivora and opens up new questions about the tooth evolution of *P. lotor* and other procyonids.

## Graphical abstract

The masticatory cycle of *Procyon lotor* was analysed and visualised, elucidating the occlusal relationships of its cheek dentition. Grinding and crushing were found to play a major role in the tooth function of *P. lotor*. An OFA analysis furthermore revealed an occlusal relationship between the M1 metaconule and the trigonid basin of the m2, indicating that the M1 metaconule replaces the function of the M1 hypocone, opening up new questions about the tooth evolution of *P. lotor* and other procyonids.

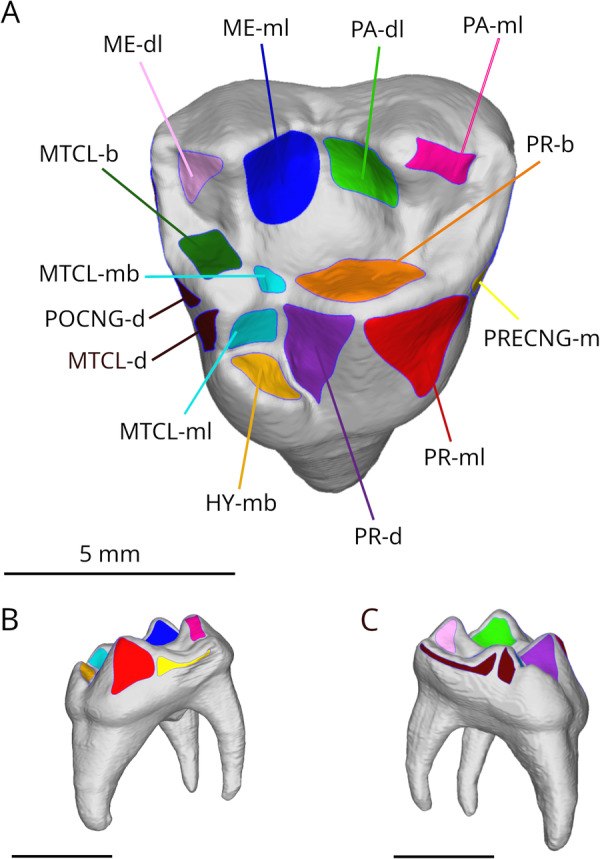

## 1 Introduction

The dentition of mammals is often regarded as a defining feature of the group (Ungar 2010). Complex mammalian teeth allow for efficient processing of food and increased energy uptake, and even though mammals are not the only animals in Earth's history to have chewed (Varriale 2016), the degree of dental specialisation seen in mammals in order to optimise mastication is not known from elsewhere in the animal kingdom (Ungar 2010). Through several evolutionary steps, the simple haplodont dentition of early synapsids evolved into shearing‐adapted triconodont dentition with precise occlusion and ultimately into the multi‐cusped tribosphenic molar that was adapted to a wider range of tooth functions, including crushing and grinding (Crompton 1971; Davis 2011; Martin et al. 2020). This type of molar is ancestral to all boreosphenidan molars, and is the basis for the current diversity of mammalian molar morphologies (Cope 1883; Martin et al. 2020; Osborn 1888, 1907). The group Carnivora is characterised by specialised cheek teeth that are used for shearing meat: the carnassials. These carnassials are made up of the P4 and the m1 and the molars distal to these are the postcarnassials. While this is the ancestral condition of the order, Carnivora is a diverse group, the members of which have developed tooth adaptations to all diets ranging from hypercarnivory to strict herbivory (Ewer 1973; Mittermeier and Wilson 2009).

*Procyon lotor*, commonly known as the northern raccoon, is a member of the family Procyonidae, a taxon of omnivorous species within the Caniformia (Ewer 1973; Mittermeier and Wilson 2009). Raccoons primarily reside in marshes and wooded wetlands, making use of the food sources these places provide (Goldman 1950; Lotze and Anderson 1979). They are generally considered hypocarnivores, as their diet consists of > 70% non‐vertebrate food (Van Valkenburgh 2007). Since raccoons are opportunistic generalists, their diets reflect the seasonal, geographical and ecological availability of food items (Ewer 1973; Mittermeier and Wilson 2009; Rulison, Luiselli and Burke 2012). Table 1 shows an overview of the dietary components of *P. lotor* per season.

**Table 1:** Dietary components of *P. lotor* per season as recorded by various sources. ND = no data.

	Vegetable matter			Animal matter			
Source	Fruits	Nuts and grains	Other	Crustaceans	Insects	Other invertebrates	Vertebrates
	**Spring**						
Baker et al. (1945)	6	22	0	57	5	1	8
Dorney (1954)	0	~20.5^a^	0	~29.5^a^	~2^a^	~1.5^a^	~45.5^a^
Giles (1940)	0	~47.8^a^	~18.5^a^	~11.3^a^	~17.5^a^	~0.4^a^	~4.1^a^
Hamilton (1951)	ND	ND	ND	ND	ND	ND	ND
Schoonover and Marshall (1951)	ND	ND	ND	ND	ND	ND	ND
Stuewer (1943)	0.1	39	4.1	14.7	3.2	8.1	30.9
Yeager and Rennels (1943)	ND	ND	ND	ND	ND	ND	ND
	**Summer**						
Baker et al. (1945)	27	26	0	29	12	2	4
Dorney (1954)	26	1	0	31	0	3	39
Giles (1940)	46.2	16.8	0	0	25.5	0	4.4
Hamilton (1951)	37.9	14.7	6.1	0	8.2	9.1	23.2
Schoonover and Marshall (1951)	34.9	9.7	0.1	31	11.8	0.1	5.6
Stuewer (1943)	77.3	0	0	1.7	19.3	1.7	0
Yeager and Rennels (1943)	ND	ND	ND	ND	ND	ND	ND
	**Autumn**						
Baker et al. (1945)	56	24	0	15	4	0	0
Dorney (1954)	41	9	0	16	0	10	24
Giles (1940)	1.1	85.2	0	2.1	10.1	0	1.2
Hamilton (1951)	ND	ND	ND	ND	ND	ND	ND
Schoonover and Marshall (1951)	ND	ND	ND	ND	ND	ND	ND
Stuewer (1943)	68.4	23.9	0.4	2.4	3.2	1.1	0.7
Yeager and Rennels (1943)	46.1	19.6	6.3	8.6	8	3.4	7.5
	**Winter**						
Baker et al. (1945)	~17^a^	~57^a^	0	~10^a^	~8.5^a^	~1^a^	~3.5^a^
Dorney (1954)	ND	ND	ND	ND	ND	ND	ND
Giles (1940)	ND	ND	ND	ND	ND	ND	ND
Hamilton (1951)	ND	ND	ND	ND	ND	ND	ND
Schoonover and Marshall (1951)	ND	ND	ND	ND	ND	ND	ND
Stuewer (1943)	0.9	54.5	18.2	0	8.2	18.2	0
Yeager and Rennels (1943)	ND	ND	ND	ND	ND	ND	ND

^a^ Values are averages of two data points.

**Figure 1:**
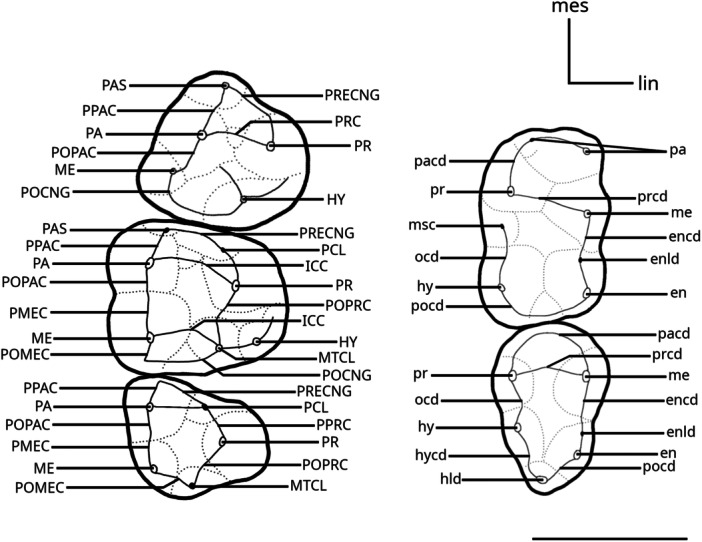
Schematic drawing of (on the left, from top to bottom) the upper P4, M1 and M2 of the right side of the jaw and (on the right, from top to bottom) the lower m1 and m2 of the left side of the jaw. Cusps and crests are labelled. See Table 2 for an overview of the anatomical abbreviations. The scale bar on the bottom right indicates 10 mm.

As an adaptation to its omnivorous diet, the fourth maxillary premolar of the raccoon, the tooth that ancestrally makes up the upper carnassial in Carnivora, not only has a large buccal paracone and metacone and lingual protocone, but likewise supports a prominent lingual hypocone (Ahrens 2012; Gorniak 1986) (see Figure 1 for a schematic overview of the P4, M1, M2, m1 and m2 of *P. lotor*). Like the P4, the M1 possesses a lingual hypocone, although it is reduced and shifted lingually in comparison to that of the P4 (Ahrens 2012; Rodriguez et al. 2016). The m1, the lower carnassial in Carnivora, lacks the blade‐like paracristid used by many carnivorans for shearing meat (Ahrens 2012). Instead, the large paraconid, metaconid and protoconid of the trigonid of the m1 are positioned with approximately equal distances from each other and the paracristid and protocristid are low. The paraconid is quite large and bifid in *P. lotor* (Ahrens 2012; Baskin 2004). Distal to the trigonid, there is a well‐developed talonid basin on the m1, bounded by a buccal hypoconid and a lingual entoconid. Mesial to the entoconid is an additional small entoconulid that is attached to the larger conid with a crest. The M2 of *P. lotor* is significantly smaller than the M1, and only contains three large cones compared to the four cones and additional large conule of the M1 (Gorniak 1986; Rodriguez et al. 2016). Buccally situated are the mesial paracone and distal metacone, and lingually there is a large protocone. A hypocone is absent in the M2 (Ahrens 2012). The m2 lacks a paraconid, so the most mesial conids on the m2 are the buccal protoconid and the lingual metaconid (Ahrens 2012; Rodriguez et al. 2016). Distal to the protoconid there is a hypoconid and distal to the metaconid there is an entoconid with a small associated entoconulid. On the far distal side of the m2 there is a hypoconulid present, unlike on the m1.

During the power stroke of the mastication cycle, teeth come into contact with food and any grit or dirt associated with it (abrasion), as well as opposing teeth (attrition), which wears down the dentition (Butler 1972; Stones 1948). While abrasion is the most common type of wear (Fortelius 1985), different wear patterns may arise when varying quantities of food are included between two wear surfaces (Fortelius 1985; Rensberger 1978). For example, antagonistic teeth that are closely associated during occlusion result in highly polished wear facets with clearly distinguishable boundaries, whereas dull or textured surfaces without sharp boundaries may be the result of the interposition of food between the antagonistic surfaces, which, therefore, do not come in close contact with each other (Rensberger 1978).

A wear facet analysis is one of the most common methods for describing the occlusal relationships in the dentition of mammals (Mills 1963; Schulz‐Kornas et al. 2020). It grants an insight into the tooth function of mammals and additionally serves as an important medium for interspecific comparison, due to the homology of many tooth structures (Butler 1972). While the tooth morphology of raccoons has previously been described by various authors (Ahrens 2012; Gorniak 1986; Rodriguez et al. 2016), no such wear facet analysis exists for this species, and the occlusal relationships between the cusps on the cheek dentition of *P. lotor* are unresearched.

This paper describes the tooth function of *P. lotor* by mapping the wear facets on the cheek teeth, defines a series with consecutive stages of abrasive wear and dentine exposure and provides a thorough analysis of the power stroke of the masticatory cycle with the associated occlusal contacts and movements using the OFA software in an effort to better understand the occlusal relationships of the cheek teeth of the raccoon.

## 2 Materials and Methods

The raccoon (*Procyon lotor* Linnaeus 1758) skulls with dentition studied here are from the mammal collection of the Leibniz‐Institut zur Analyse des Biodiversitätswandels, Museum Koenig Bonn (ZFMK) (list of specimens in Supporting Information S1: Table S1). Specimens were chosen based on the wear stages described by Anders et al. (2011). They describe IDAS 3 as the stage starting from the moment the molars are fully erupted to the complete abrasion of the cusps on the first molars. IDAS 4 is described as the period lasting from the full abrasion of the cusps on the first molar to the full abrasion of the cusps on the second molar. For the wear facet analysis, specimens exhibiting a wear stage in the early stage of IDAS 3 were chosen, so that wear facets could be distinguished and cusp height had not been reduced to the point of obstructing their visibility. For the dentine exposure series, skulls exhibiting various stages of cusp height reduction and dentine exposure (ranging from IDAS 3 to IDAS 4 according to Anders et al. 2011) were chosen. In total, 26 specimens were examined in this study, of which seven were used in the dentine exposure series (referred to as D1–D7, with D standing for dentine exposure analysis and the number representing the wear stage, with 1 being the least to 7 being the most worn) and 19 in the wear facet analysis. Epoxy casts of the upper P4, M1 and M2 as well as the lower m1 and m2 were made of both sides of the jaw of the specimens.

### 2.1 Dentine Exposure Series

Micro‐computed tomographic (μCT) scans were made of seven raccoon skulls with varying degrees of dentine exposure on their teeth. The scans of the specimens were made at the Bonn Institute of Organismic Biology, section Palaeontology, using a v|tome|x S240 microtomograph (GE Sensing & Inspection Technologies GmbH). After scanning, the crowns of the P4, M1, M2, m1 and m2 of one side of each skull were segmented with Avizo Lite version 2020.2 (Thermo Fisher Scientific Inc., Waltham, Massachusetts, USA). The generated 3D surfaces were subsequently imported into PolyWorks 2014 IR13 (InnovMetric Software Inc., Quebec City, Quebec, Canada) as polygonal models (STL format). The boundaries of the dentine exposure were outlined in the Edit function of PolyWorks by creating a curve. The area inside the curve was selected and a new polygonal surface was created with the selected triangles, representing the area of dentine exposure on the dentition.

### 2.2 Wear Facet Analysis

Wear facets were identified and documented on the tooth casts by S. Koomen using a Zeiss AXIO Zoom.V16 light microscope and subsequently the images were processed using the Zen 2 software version 2.0.0.0 (Carl Zeiss Microscopy GmbH, Jena, Germany). By tilting the teeth under oblique light, reflective planes were identified as attritional wear facets. Subsequently, the facets were mapped on a 3D model of the dentition of specimen ZFMK–MAM2013.0941 using PolyWorks. The facets were then coloured according to occlusal contact and similar placement on the tooth per tooth position.

Pictures were taken of all identified wear facets with a TESCAN VEGA S5122 Scanning Electron Microscope (SEM) (TESCAN GROUP, Brno, Czech Republic) to identify possible microwear. Tooth casts were coated for 45 s with palladium using a Cressington sputter coater 108 auto (Cressington Scientific Instruments Ltd., Watford, UK) prior to SEM study. The SEM pictures were taken and processed using the TESCAN Essence software version 1.1.9.1 build 5440 (TESCAN GROUP, Brno, Czech Republic). The pictures were taken with a speed of 7, and the resolution of the pictures was set to 2048 × 1536 pixels.

Facets were labelled following the system of Schultz et al. (2018) and Schultz et al. (2020). Abbreviations for the upper teeth are capitalised, and lower case is used for the lower teeth. Labels of wear facets consist of two parts: an abbreviation of the cusp that bears the facet and an indicator of the direction of the flank of the cusp that the facet occupies. Four directional terms are used to describe the placement of the facets: distal, mesial, lingual and buccal. If a facet is oriented in an intermediate fashion between these directions, the directional words are compounded to describe the orientation more accurately (e.g., distolingual, mesiobuccal etc.). Anatomical, directional and institutional abbreviations can be found in Table 2.

**Table 2:** List of abbreviations for crown structures, directions on the occlusal surface and institutions. Other abbreviations not included in the table are taken from Schultz et al. (2020).

Abbreviations for crown structures of mammalian molars	
encd	entocristid
enld	entoconulid
hycd	hypocristid
ICC	internal conular crista
msc	mesoconid
ocd	oblique cristid
PAS	parastyle
PMEC	premetacrista
POMEC	postmetacrista
pocd	postcristid
POCNG	postcingulum
PPAC	preparacrista
POPAC	postparacrista
PPRC	preprotocrista
POPRC	postprotocrista
PRC	protocrista
PRECNG	precingulum
**Directional abbreviations**	
buc/b	buccal
dis/d	distal
lin/l	lingual
mes/m	mesial
**Institutional abbreviations**	
ZFMK‐MAM	Mammal collection of the Leibniz‐Institut zur Analyse des Biodiversitätswandels, Museum Koenig Bonn, Germany

### 2.3 OFA Analysis

The occlusal relationships between antagonistic tooth structures were studied using the OFA method (Kullmer et al. 2009; Kullmer et al. 2020). The OFA software (ZiLoX IT GbR, Wallhausen, Germany, https://www.paleontology.uni-bonn.de/de/forschung/ehemalige-forschergruppen/for-771-ofa/occlusal-fingerprint-analyser-ofa) allows for a virtual reconstruction of the masticatory cycle including a quantitative analysis of tooth contacts and has already been applied to diverse mammalian taxa, such as primates (Kullmer et al. 2020), morganucodontans (Jäger et al. 2020; Jäger et al. 2019), dryolestids and didelphids (Schultz and Martin 2014), as well as dasyuromorphs and carnivorans (Lang and Martin 2024). A total of 5 path points were placed to define the movement of the lower teeth with respect to the upper teeth of *P. lotor*, starting with the point representing centric occlusion, or the point where the M1 protocone occludes in the talonid basin of the m1 (Kay and Hiiemae 1974). The upper molars were translated and rotated to match the occluding structures of the opposing teeth. The modelled movement of the lower dentition was divided into time steps, for which the sizes of the contact areas are recorded in mm^2^. The maximum degree for break free was set to 300 and the distance to 0.20. The final step length was set to 0.1.

For visualisation of the movements of the lower teeth relative to the upper teeth of the active side of the jaw during the power stroke of the mastication cycle, a mastication compass (von Koenigswald et al. 2013) was used. A mastication compass consists of a circle with anatomical directions. The arrows drawn in the compass represent the phases of the power stroke. The direction or angle of the arrows represents the direction of the movement of the lower dentition relative to the upper dentition on the working side of the jaw, and the length of the arrows represents the inclination during this movement, with longer arrows signifying a more horizontal movement and shorter arrows signifying a larger vertical component. Both measures are given in degrees. The mastication compass for *P. lotor* was created by exporting the path points of the movements of the lower molars during the power stroke calculated by the OFA. Subsequently, the occlusal compass was fashioned using GIMP version 2.10.38 (GIMP Development Team).

All descriptions of movement, occlusion and tooth function regard the working side of the jaw.

## 3 Results

### 3.1 Facets

A total of 58 facets were observed on the P4, M1, M2, m1 and m2 of *P. lotor*. Pictures of the facets taken using the digital microscope and the SEM can be found in the Supporting Information Data (Supporting Information S1: Figures S1–S20).

#### 3.1.1 P4

**Figure 2:**
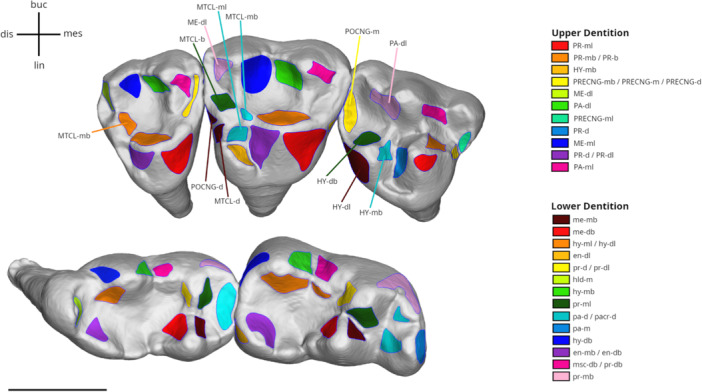
Overview of the recorded wear facets of the upper P4, M1 and M2 and the lower m1 and m2 in occlusal view. Facets on the lower teeth are coloured according to similar placement on the tooth and those of the upper teeth are coloured according to contact to antagonistic facets. The scale bar represents 5 mm. An overview of the abbreviations used can be found in Table 2.

On the P4, a total of 11 facets has been recorded (see Figure 2). Mesially‐located on the tooth are two facets that occupy the precingulum, which starts at the well‐developed P4 parastyle and runs lingually where it connects to the protocone. Of the two facets that lie on the precingulum, facet P4‐PRECNG‐ml (see Supporting Information S1: Figure S5B,C) faces mesiolingually and facet P4‐PRECNG‐mb (see Supporting Information S1: Figure S5A,C) faces mesiobuccally. Both facets meet at the deepest point of the precingulum.

The paracone, which is the middle of three large buccal cusps on the P4, supports two facets. Its mesiolingual face is occupied by facet P4‐PA‐ml (see Supporting Information S1: Figure S6C,D). This facet is long and narrow and has remarkably straight edges. Another facet, P4‐PA‐dl (see Supporting Information S1: Figure S6A,B) lies on the distolingual side of the paracone. This side of the paracone is continuous with the metacone and forms one uninterrupted area facing in distolingual direction. Facet P4‐PA‐dl occupies this area in part, although its extension onto the metacone differs per specimen. No clear boundary can be distinguished at the base of the cusp.

The protocone of the P4 supports three facets, one of which faces mesiobuccally, another which faces mesiolingually and a third one that faces distally. P4‐PR‐mb (see Supporting Information S1: Figure S7A,B) extends from the tip to the base of the protocone. Buccally, the facet ends where the crest connecting the protocone and the paracone originates on the protocone. The second facet on the protocone, P4‐PR‐ml (see Supporting Information S1: Figure S7C,D), starts on the mesial face of the protocone, where it contacts facet P4‐PR‐mb, and from there it wraps around the tip of the protocone until it reaches a mesiolingual orientation. Facet P4‐PR‐d (see Supporting Information S1: Figure S7E,F) faces distally on the protocone and starts at its base. It is defined both buccally and lingually by the curvature of the cusp.

Flanking P4‐PR‐d at the base of the protocone is facet P4‐HY‐mb (see Supporting Information S1: Figure S8A,B). This facet faces mostly mesially and slightly buccally and is located on the hypocone of the P4. Two other facets have been observed on the hypocone. One of these is P4‐HY‐dl (see Supporting Information S1: Figure S8C,D), which occupies a distolingual area on the hypocone. The third facet, P4‐HY‐db (see Supporting Information S1: Figure S8E,F), is situated distobuccally on the hypocone. The facet seems to gradually fade towards the base of the cusp.

A fourth facet was observed in some specimens on the mesial facing area of the crest connecting the metacone and the hypocone. This facet, P4‐POCNG‐m (see Supporting Information S1: Figure S9), is likely continuous with facet M1‐PRECNG‐m on the M1.

#### 3.1.2 M1

The first upper molar of *P. lotor* bears 14 attritional wear facets that are positioned on the various structures of the tooth. Figure 2 shows the position of these wear facets on the molar.

A total of four facets are located on the lingual sides of the buccally‐placed para‐ and metacone. Starting at the paracone, facet M1‐PA‐ml (see Supporting Information S1: Figure S1A,B) occupies the mesiolingual face of the paracone on the M1 and is bounded by the preparacrista and the internal conular crista connecting the paracone to the paraconule. On the distolingual side of the paracone there is another facet facing the basin of the trigon of the M1. This facet, M1‐PA‐dl (see Supporting Information S1: Figure S1C,D), spans between the postparacrista and the internal conular crista that connects with the paracone. Flanking this facet distally is facet M1‐ME‐ml (see Supporting Information S1: Figure S1E,F), on the mesiolingual side of the metacone. The facet's boundaries are defined by the premetacrista and a crest running from the metacone to the large metaconule. Occupying the distolingual face of the metacone is facet M1‐ME‐dl (see Supporting Information S1: Figure S1G,H), which does not follow the internal conular crista that connects the metacone to the metaconule, but instead follows the gentler slope of the metacone diagonally downwards from the tip of the cusp down to the base of the postcingulum.

Facet M1‐PRECNG‐m (see Supporting Information S1: Figure S2A,B) occupies the mesial side of the precingulum, often spanning the entire length of the crest from the parastyle to the small paraconule at the side of the protocone. The facet seems continuous with a small facet on the mesial side of the paraconule, although they are separated from each other by an angle.

Contacting this facet lingually is facet M1‐PR‐ml (see Supporting Information S1: Figure S2C,D). This facet is positioned on the mesiolingual side of the M1 protocone. The borders of this facet are formed by the curvature of the protocone on the lingual side and the start of the precingulum on the mesiobuccal side. Positioned on the relatively flat distal side of the protocone is facet M1‐PR‐d (see Supporting Information S1: Figure S2E,F). The protocone also supports a facet, M1‐PR‐b (see Supporting Information S1: Figure S2G,H), on its buccal side. No clearly marked boundary is visible towards the base of the facet. Flanking facet M1‐PR‐d distolingually, facet M1‐HY‐mb is located on the mesiobuccal face of the hypocone (see Supporting Information S1: Figure S4A,B).

Five more facets occur on the enlarged metaconule. One of these (M1‐MTCL‐ml) (see Supporting Information S1: Figure S3A,B) contacts facet M1‐HY‐mb lingually and facet M1‐PR‐d mesially and occupies the mesiolingual side of the metaconule. Facet M1‐MTCL‐mb (see Supporting Information S1: Figure S3C,D) occurs on the mesiobuccal side of the metacone. The facet usually occurs directly on the postprotocrista, causing this to wear down. Facet M1‐MTCL‐b (see Supporting Information S1: Figure S3G,H) is found on the buccal side of the metaconule and is bounded by the postcingulum and the internal conular crista that connects the metacone with the metaconule. No clear boundary is present at the base of the facet. On the distal side of the M1 metaconule, M1‐MTCL‐d (see Supporting Information S1: Figure S3D) is bordered buccally by facet M1‐POCNG‐d. No clear boundary could be observed towards the base of the facet, nor on its lingual side. Facet M1‐POCNG‐d (see Supporting Information S1: Figure S4C,D) occupies the distal side of the postcingulum. The facet has a slender and elongated shape and starts at the distobuccal side of the metaconule, in some cases following the postcingulum all the way distally to the metacone. The facet does not reach the metacone where the M2 is so high as to block part of the postcingulum of the M1.

#### 3.1.3 M2

Eight attritional wear facets have been observed on the surface of the upper second molar of *P. lotor* (see Figure 2). Two facets are situated on each of the two buccal cusps (the paracone and the metacone) of the tooth, with the same placement as those on the M1.

M2‐PA‐ml (see Supporting Information S1: Figure S10A,B) is located on the mesiolingual side of the paracone of the M2. The facet follows the internal conular crista between the paracone and the paraconule along its apical border. Its buccal border is formed by the preparacrista. Another facet lies on the distolingual side of the paracone. This facet, M2‐PA‐dl (see Supporting Information S1: Figure S10C,D), occupies the entirety of the distolingual face of the paracone from its tip to its base, although a basal border cannot be distinguished. On the mesiolingual side of the metacone, facing the trigon basin of the M2 is facet M2‐ME‐ml (see Supporting Information S1: Figure S10E,F). Like facet M2‐PA‐dl, this facet often occupies a large area on the metacone, although it can sometimes only be found on the tip of the cusp. Facet M2‐ME‐dl (see Supporting Information S1: Figure S10G,H) is positioned on the distolingual side of the metacone. It leads from the tip of the metacone to the base of the postcingulum.

The protocone supports three wear facets, quite similarly to the M1. Both the mesial and distal side of the lingual‐facing portion of the protocone bears a facet that is relatively large and can be described as having a half‐moon shape. Of these, M2‐PR‐ml (see Supporting Information S1: Figure S11A,B) follows the preprotocrista and M2‐PR‐dl (see Supporting Information S1: Figure S11C,D) is situated along the postprotocrista. There is another facet on the buccal side of the protocone, facing internally into the trigon basin of the M2: M2‐PR‐b (see Supporting Information S1: Figure S11E,F). This facet resembles facet M1‐PR‐b of the first upper molar in having no clearly defined boundaries except the pre‐ and postprotocrista that flank the protocone. The basal side of the facet fades gradually and leads into the trigon.

Contacting M2‐PR‐b to its distobuccal side is facet M2‐MTCL‐mb (see Supporting Information S1: Figure S12A,B). This small facet occupies the mesiobuccal side of the metaconule and is defined by the edges of this conule. M2‐PRECNG‐d (see Supporting Information S1: Figure S12C) is a facet that can be found on the distal side of the precingulum of the M2. The facet reaches from the parastyle to the small paraconule mesiobuccal of the protocone and follows the crest of the precingulum.

#### 3.1.4 m1

On the first mandibular molar, 13 facets have been observed in the specimens that are antagonistic to the ones of the upper cheek teeth (see Figure 2).

On the mesial side of the paraconid of the m1, facet m1‐pa‐m (see Supporting Information S1: Figure S13A,B) is situated. The paraconid is bifid in raccoons, with one lingual and one buccal cusplet. Facet m1‐pa‐m only occupies the mesial side of the lingual cusplet of the paraconid, and does not reach to the second cusplet. When the tooth comes into occlusion with the upper teeth, facet m1‐pa‐m comes into contact with facet P4‐PR‐d of the P4. Distobuccally situated to facet m1‐pa‐m on the mesiobuccal side of the protoconid is facet m1‐pr‐mb (see Supporting Information S1: Figure S13C,D). This facet forms the antagonist to the facet on the distolingual area between the paracone and metacone of the P4 (P4‐PA‐dl).

During occlusion, the hypocone of the P4 slides into the trigonid of the m1, which results in the presence of some wear facets on the internal faces of the para‐, proto‐ and metaconid of this tooth. Facet m1‐pa‐d (see Supporting Information S1: Figure S14A,B,E) can be divided into two components, each of which is associated with one of the two cusplets of the paraconid. The first starts at the distal side of the tip of the lingual cusplet of the paraconid and leads straight down to the base of the trigonid. The second is a small facet that occupies the distolingual face of the buccal cusplet of the paraconid. In some cases, the two components of the facet are connected to each other in a continuous facet that reaches from the lingual cusplet of the paraconid to the buccal cusplet and follows the distobuccal curvature of the paraconid (e.g., ZFMK‐MAM‐2016.0945). The opposing wear facet on the hypocone of the P4 is facet P4‐HY‐mb. Facet m1‐pr‐ml (see Supporting Information S1: Figure S14D,E) faces internally into the trigonid and is located on the mesiolingual side of the protoconid. The distal side of the facet follows the protocristid until it reaches its deepest point. From here the facet leads downward to the base of the trigonid basin. A straight edge between the base of the trigonid and the tip of the protoconid marks the mesial boundary of the facet. During occlusion, this facet comes into contact with the buccal side of the P4 hypocone, leaving facet P4‐HY‐b at the site of contact. The metaconid supports a triangular facet m1‐me‐mb (see Supporting Information S1: Figure S14C,E) on its mesiobuccal side. The boundaries of the facet are made up by the protocristid on the distal side of the facet and by the curvature of the metaconid on the mesial side. In occlusion, this facet touches the distolingual facet on the P4 hypocone (facet P4‐HY‐dl).

The protoconid and the metaconid each bear a facet on their distal sides, facing the talonid basin. Facet m1‐pr‐d (see Supporting Information S1: Figure S15A,B) is a facet on the distal side of the protoconid that occupies the tip of the cusp and is bounded by the buccal curvature of the protoconid. This facet is the antagonist to the elongated facet along the precingulum of the M1 (M1‐PRECNG‐m). During occlusion, the facet shears upward along this crest and might continue onto the postcingulum of the P4 (P4‐POCNG‐m), where this is close enough to the precingulum of the M1. Facet m1‐me‐db (see Supporting Information S1: Figure S15C,D) occupies the distobuccal face of the metaconid and is the antagonistic facet to facet M1‐PR‐ml on the M1.

Two other facets line the walls of the talonid basin. Facet m1‐hy‐ml (see Supporting Information S1: Figure S15E,F) is positioned on the mesiolingual face of the hypoconid of the m1. Mesiobuccally, the oblique cristid forms the border of the facet, while on the distolingual side the facet ends where the hypoconid starts to curve lingually. There is no clear border at the base of the facet. During occlusal contact, the facet collides with facet M1‐PR‐b. The second facet is facet m1‐en‐mb (see Supporting Information S1: Figure S15G,H). Facet m1‐en‐mb occupies the mesiobuccal face of the entoconid. The facet continues to the tip of the entoconid and the basal border, like in facet m1‐hy‐ml, is absent. This facet is the antagonistic facet of M1‐PR‐dl.

Distal to the protoconid there is a small cusp that is labelled as the mesoconid by Rodriguez et al. (2016). On the distobuccal face of this cusp lies a triangular facet that extends from the tip of the cusp along the oblique cristid until the base of the hypoconid; m1‐msc‐db (see Supporting Information S1: Figure S16A,B). The antagonistic facet on the opposing tooth, the M1, is facet M1‐PA‐ml. Flanking facet m1‐msc‐db distally is facet m1‐hy‐mb (see Supporting Information S1: Figure S16C,D). This facet is positioned on the mesiobuccal side of the hypoconid and like facet m1‐msc‐db occupies a triangular area. The opposing facet (M1‐PA‐dl) is positioned distolingually on the paracone of the M1.

Two more facets are located on the distal end of the m1. One of these is m1‐hy‐db (see Supporting Information S1: Figure S16E,F), which is placed on the distobuccal side of the hypoconid. This small oval‐shaped facet is the antagonist to M1‐ME‐ml and nearly reaches from the midline of the tooth to the tip of the hypoconid. The other is facet m1‐en‐dl (see Supporting Information S1: Figure S16G,H). This facet occupies a small oval area near the distolingual side of the tip of the entoconid and antagonises M1‐HY‐mb.

#### 3.1.5 m2

The m2 has 12 facets on its surface (see Figure 2).

The m2 lacks a paraconid and in the locus where it would have been, on the mesialmost portion of the tooth, the paracristid forms a ridge resembling a cingulid. Facet m2‐pacr‐d (see Supporting Information S1: Figure S17A,B) is positioned on the distal flank of this ridge. Apically, it follows the curve of the crest and basally contacts the protoconid and the metaconid. It contacts facet M1‐MTCL‐mb during the power stroke of a mastication cycle.

Facet m2‐pr‐mb (see Supporting Information S1: Figure S17G) is a small and narrow facet on the mesiobuccal side of the protoconid of the m2 and extends along the paracristid. During occlusion, the facet makes contact with facet M1‐ME‐dl. On the mesiolingual side of the m2 protoconid, there is facet m2‐pr‐ml (see Supporting Information S1: Figure S17C,D). This facet makes up part of what would have been the trigonid basin of the m2, had the paraconid not been absent. The facet forms the antagonist to facet M1‐MTCL‐b on the M1. Another facet is situated on the distolingual side of the protoconid. This facet, m2‐pr‐dl (see Supporting Information S1: Figure S17E,F), faces the talonid basin and is bounded on the apical side by the protocristid and a small section of the oblique cristid. Its antagonistic facet sits on the distal side of the precingulum of the M2 (M2‐PRECNG‐d).

Distal to the main cusp of the protoconid, there is a structure resembling a second cusplet. On the m1, this structure is interpreted to be a mesoconid by Rodriguez et al. (2016) (see Fig. 1 in Rodriguez et al. 2016). However, on the second lower molar, this structure is not explicitly named by Rodriguez et al. (2016), hence, in this study, this structure is considered as part of the protoconid. On the distobuccal face of this distal cusplet of the protoconid lies a small facet, here called m2‐pr‐db (see Supporting Information S1: Figure S18A,B). This facet spans from the tip of the protoconid to its base and contacts the base of the hypoconid. The antagonistic facet of m2‐pr‐db is M2‐PA‐ml.

Flanking facet m2‐pr‐ml to its lingual side is facet m2‐me‐mb (see Supporting Information S1: Figure S18C,D), which occupies the mesiobuccal face of the metaconid. During occlusion, facet m2‐me‐mb makes contact with wear facet M1‐MTCL‐d. Facet m2‐me‐db (see Supporting Information S1: Figure S18E,F) lies on the distobuccal side of the metaconid of the m2. There is no clear boundary at the base of the facet and the main attritional surface seems to be placed near the tip of the cusp. The antagonistic facet on the upper teeth is facet M2‐PR‐ml.

The talonid basin is bordered by three cusps in the m2: the buccal hypoconid, the lingual entoconid and a central hypoconulid, which lies distally to the hypo‐ and entoconid. Located distolingually on the hypoconid, and oriented internally into the talonid basin, is facet m2‐hy‐dl (see Supporting Information S1: Figure S19A,C). The facet occupies the tip of the hypoconid and gradually fades at the base of the cusp. During the power stroke, the lingual portion of the facet makes contact with facet M2‐PR‐b and the distolingual portion that is closest to facet m2‐hy‐db makes contact with facet M2‐MTCL‐mb. As the two parts of the facet are antagonised by two different structures on the upper teeth, the parts could potentially be interpreted as two separate facets. However, as there is no clear boundary between the parts and because they appear on the same structure—the hypoconid—they are considered as one in this study.

Facet m2‐hy‐db (see Supporting Information S1: Figure S19B,C) occupies the distobuccal face of the hypoconid of the m2 and flanks facet m2‐hy‐dl distolingually. It is bordered on the lingual side by the hypocristid and has an edge on the buccal side where the hypoconid curves buccally. The facet furthermore forms the antagonistic facet to M2‐ME‐ml. Facet m2‐hy‐mb (see Supporting Information S1: Figure S19D,E) occupies the mesiobuccal side of the hypoconid and is the antagonistic facet of M2‐PA‐dl. It reaches from the tip of the hypoconid down to the groove separating it from the protoconid, where the facet meets the distal edge of m2‐pr‐db. Along its apical edge, the facet follows the oblique cristid.

Facet m2‐en‐db (see Supporting Information S1: Figure S20A) is situated on the distobuccal side of the entoconid and is the antagonistic facet of M2‐PR‐dl on the M2. The facet is bounded on the apical side by the postcristid that runs from the entoconid to the hypoconulid, and ends at the most distal border of the entoconid. Along the base of the entoconid, the edge cannot be distinguished clearly.

Facet m2‐hld‐m (see Supporting Information S1: Figure S20B) sits on the distal hypoconulid and faces mesially. It is the antagonist to facet M2‐ME‐dl on the distolingual side of the M2 metacone. The facet often occupies the full mesial face of the hypoconulid. In some cases, the facet even connects with facet m2‐en‐db to form one large continuous facet along the mesiobuccal face of the entoconid and the hypoconulid.

### 3.2 OFA Analysis

**Figure 3:**
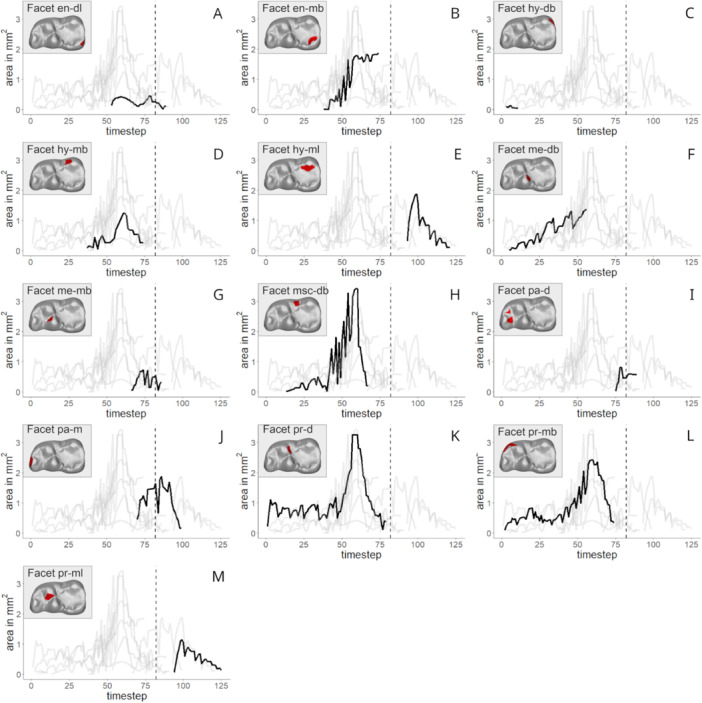
Line graphs showing the size of the contact areas in mm^2^ per time step of the OFA of individual facets for the lower m1. The dashed line marks the timestep of centric occlusion.

**Figure 4:**
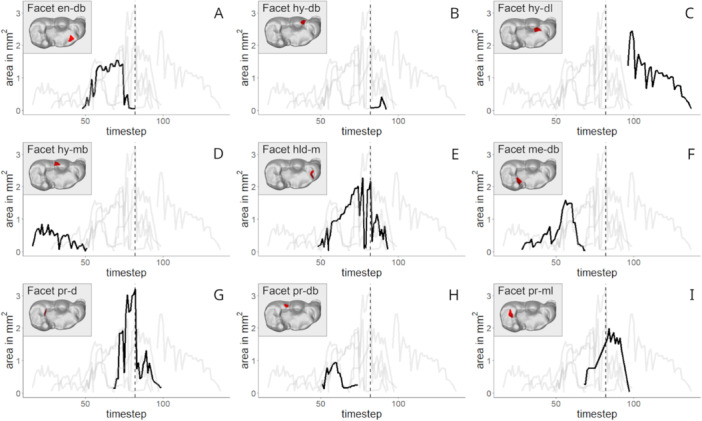
Line graphs showing the size of the contact areas in mm^2^ per time step of the OFA of individual facets for the lower m2. The dashed line marks the timestep of centric occlusion.

An OFA analysis was performed to measure the time of contact and the size of the contact areas between the P4, M1 and M2 and the m1 (Figure 3) and m2 (Figure 4) during the power stroke. The path of the power stroke calculated with the OFA software consists of 137 time steps, with centric occlusion (the point where the protocone of the M1 fully occludes with the talonid basin of the m1) occurring at time step 82. Tables with area per timestep can be found in the Supporting Information Data (Supporting Information S1: Tables S2 and S3). Not all facets observed in the wear facet analysis were supported by the OFA analysis. This is explained by the fact that not all wear facets may be present on the dentition of every individual specimen.

The first contact between the upper and lower cheek teeth of the raccoon takes place between facet M1‐PA‐ml on the mesiolingual side of the M1 paracone and the distal facet on the m1 protoconid (m1‐pr‐d) during time step 1 of the power stroke (Figure 3K). The tip of the m1 protoconid collides with the parastyle of the M1 in this time step. At time step 5, a contact area between the distobuccal side of the metaconid of the m1 (m1‐me‐db) and the mesiolingual side of the M1 protocone (M1‐PR‐ml) arises (Figure 3F). Between time steps 57 and its last moment of contact at time step 65, this contact area merges with the one on the distal side of the protoconid of the m1 (m1‐pr‐d), but should be considered as two separate facets.

At time step 2, a second site of contact occurs between the mesial side of the m1 paraconid and the distolingual tip of the paracone of the P4 (P4‐PA‐dl). The buccal side of the paraconid subsequently comes into occlusion and the mesiobuccal side of the buccal cusplet of the bifid paraconid occludes with the P4 paracone around time step 45. At time step 51, the mesiobuccal side of the m1 protoconid (m1‐pr‐mb) occludes with the distolingual side of the P4 metacone (part of facet P4‐PA‐dl) (Figure 3L). These cusps remain in contact until time step 71, although momentarily losing contact at time step 52.

Between time steps 3 and 7, a collision between the distobuccal side of the m1 postcristid (this corresponds to the facet on the distobuccal side of the hypoconid, m1‐hy‐db) and the mesiolingual side of the M1 metacone (M1‐ME‐ml) occurs (Figure 3C). This contact is lost for a moment between time steps 8–10 and then reappears for one time step at time step 11.

A new point of contact arises on time step 13 between the tip of the mesoconid on the m1 and the tip of the M1 paracone. As the lower dentition moves upward, the distobuccal side of the m1 mesoconid (m1‐msc‐db) and the mesiolingual side of the M1 paracone (M1‐PA‐ml) come into contact (Figure 3H). The contact is interrupted during time steps 16–17, 21, 25 and 35–36, and stops after time step 67. Between time steps 20 and 37, the contact area on the m1 occasionally merges with that on the mesiobuccal side of the hypoconid of the m1 (m1‐hy‐mb) (Figure 3D). The collision area on the mesiobuccal side of the m1 hypoconid first appears in time step 18 and finally disappears after time step 51.

The first point of contact on the m2 appears in time step 16. This contact area is formed by the collision of the mesiobuccal side of the m2 hypoconid (m2‐hy‐mb) with the distolingual side of the M2 paracone (M2‐PA‐dl) (Figure 4D). The contact is interrupted during time steps 40, 44 and 49 and the final contact between these structures occurs at time step 51.

At time step 28, the distobuccal side of the m2 metaconid (m2‐me‐db) occludes with the mesiolingual side of the paraconule on the M2 (Figure 4F). From time step 53, the lower dentition moves upwards to bring the mesiolingual side of the protocone (M2‐PR‐ml) and the distolingual edge of the metaconid in occlusion with each other. The mesial portion of the contact area on the m2 gradually fades between time steps 56 and 64, and the contact stops completely after time step 69.

A contact area on the buccal side of the entoconid of the m1 (referred to previously as m1‐en‐mb) appears for the first time on the mesially‐situated entoconulid at time step 38 (Figure 3B). The opposing contact area on the upper teeth starts at the tip of the M1 protocone (M1‐PR‐dl). The contact area on the entoconulid grows until it is accompanied by a contact area on the mesiobuccal side of the entoconid from time step 42 onwards. The contact area is lost during time steps 40 and 44, and disappears completely after time step 74.

A contact area on the buccal side of the entoconid of the m2 (referred to previously as m2‐en‐db) appears at time step 48 (Figure 4). The mesial entoconulid first comes into collision with the tip of the protocone of the M2, where the contact area grows distolingually (M2‐PR‐dl). At time step 51, the buccal side of the entoconid of the m2 occludes with the M2 and the distobuccal side of the entoconid does so at time step 54. The two contact areas on the entoconid merge with each other from time step 68 onwards, and disappear completely after time step 82. The contact area on the entoconulid disappears before that of the entoconid, at time step 75, and is absent during time step 49. At time step 48, a contact area furthermore appears on the mesial side of the hypoconulid on the m2 (m2‐hld‐m) (Figure 4E). This collision area disappears once at time step 49, but then continues to grow along on the mesial side of the hypoconulid, and from time step 76 it starts to expand mesiobuccally towards the hypoconid. The contact area finally disappears after time step 93.

At time step 51, the distobuccal face of the protoconid of the m2 (m2‐pr‐db) occludes with the mesiolingual side of the paracone of the M2 (M2‐PA‐ml) (Figure 4H). These structures are in contact continuously until time step 75.

Between time steps 53 and 89, there is a contact area on the distal side of the entoconid of the m1 (Figure 3A). This area is in contact with the mesiobuccal side of the metaconule on the M1 (M1‐MTCL‐mb). As the lower dentition moves lingually, the m1 postcristid occludes with the opposing tooth. In this case, the contact area that is observed on the distal side of the postcristid of the m1 serves as an extension of facet m2‐pacr‐d.

The first point of contact in the trigonid of the m1 occurs between the mesiobuccal face of its metaconid (m1‐me‐mb) and the distolingual side of the hypocone of the P4 (P4‐HY‐dl) (Figure 3G). This contact starts at time step 66 at the tip of the metaconid and extends to further down the mesiobuccal side of the cusp until it disappears after time step 86.

Two points of contact appear simultaneously at time step 68 when the protoconid of the m2 is wedged between the M1 and M2. The distal side of the protoconid (m2‐pr‐d) collides with the precingulum of the M2 (M2‐PRECNG‐m) (Figure 4G), while the mesial side of the protoconid (m2‐pr‐ml) is in contact with the distal side of the postcingulum of the M1 (M1‐POCNG‐d) (Figure 4I). Between time steps 72 and 82, the two contact areas occasionally merge with each other in the OFA analysis, although they should be considered as two separate facets. From time step 76 onwards, the contact area on the mesial side of the m2 protoconid extends into the basin to its mesial side and to the lingual side of the buccalmost portion of the paracristid. The contact area on the mesial side of the m2 protoconid disappears after time step 97, while the one on its distal side disappears two time steps later.

At time step 70, a collision occurs between the mesial side of the paraconid of the m1 (m1‐pa‐m) and the distal side of the P4 protocone (P4‐PR‐d) (Figure 3J). Contact between these areas is lost at time steps 75, 78–79, 83 and 88–89 before the contact is finally lost completely after time step 99.

Between the distal side of the paraconid of the m1 (m1‐pa‐d) and the mesial side of the P4 hypocone (P4‐HY‐mb), there is an area of contact that lasts from time step 75 to time step 89. It is absent at time steps 85–87 (Figure 3I).

At time step 82, the lingual side of the m2 hypoconid (m2‐hy‐l) collides with the buccal side of the M2 protocone (M2‐PR‐b) (Figure 4C). The contact area on the m2 hypoconid is limited to the tip of the cusp at time step 82 and is absent at time steps 83–86, 90, 93–95, before the contact area reappears on the lingual side of the hypoconid at time step 96. Contact between the lingual side of the m2 hypoconid and the M2 protocone is lost after time step 137.

There is one small contact area on the mesial side of the postcristid of the m1 that makes contact during a total of two time steps. This is time step 87 and time step 91. This contact area is not associated with an observed wear facet and is most likely an artifact of the OFA analysis.

A collision area between the lingual side of the m1 hypoconid (m1‐hy‐ml) and the buccal side of the M1 protocone (M1‐PR‐b) originates at time step 93 (Figure 3E). Their final contact occurs at time step 121.

At time step 94, the base of the lingual side of the m1 protoconid (m1‐pr‐ml) occludes with the tip of the P4 hypocone (P4‐HY‐db) (Figure 3M). The contact area on the m1 protoconid shifts towards the tip of the cusp gradually, until contact is lost at time step 126.

The last contact is the aforementioned contact between the m2 hypoconid (m2‐hy‐l) and the M2 protocone (M2‐PR‐b) (Figure 4C). This last point of contact occurs at time step 137 of the power stroke.

The OFA analysis of the power stroke of *P. lotor* clearly shows two phases (see Figures 3 and 4). As can be seen in Figure 3, the majority of contact areas on the m1 have their first and last contact with the antagonistic surface between time step 1 and time step 91, while there are just two that only appear after time step 93, forming two distinct phases of tooth contact. One additional contact area on the mesial side of the m1 paraconid bridges the gap between both phases, being in contact from time step 70 until 99.

### 3.3 Mastication Compass

**Figure 5:**
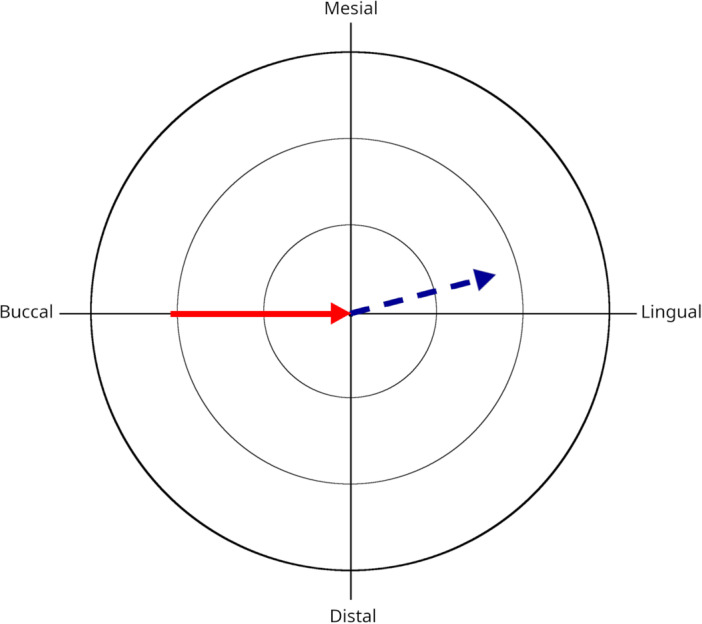
Mastication compass of *Procyon lotor* based on the OFA. The rings on the compass, from smallest to largest, represent 30, 60 and 90 degrees, respectively, and the axes represent directions on the occlusal plane. The arrows represent the different phases of the power stroke, with the red arrow indicating Phase I and the blue arrow indicating Phase II. The length of the arrows indicates the inclination of the lower jaw movement relative to the occlusal plane. The direction of the inclination of this movement is indicated by a continuous (upward movement) or dotted (downward movement) line.

The mastication compass graphically represents the movement of the lower dentition relative to the upper dentition on the working side of the jaw during the power stroke of raccoons and shows the two phases found in the OFA analysis: Phase I starts at the first contact between upper and lower teeth and ends when centric occlusion is reached, and the second, Phase II, starts at centric occlusion and continues until the last contact between the occluding teeth before the mandible lowers and the mouth opens (Figure 5).

The direction of the lower teeth during Phase I of the power stroke is nearly purely lingual, with a negligible mesial component. The lower teeth move upwards during Phase I, and do so with an inclination of 29.3° relative to the occlusal plane. For the purpose of representing this in the mastication compass, this translates to a 60.7° angle with a vector that is perpendicular to the occlusal plane.

During the second phase of the power stroke, the lower molars move mesially with an angle of 14.8°. The lower teeth furthermore move downward during the second phase of the power stroke, as indicated by the dotted line in Figure 5. The inclination relative to the occlusal plane during this movement is 35.9°, in other words, 54.1° relative to a vector perpendicular to the occlusal plane.

### 3.4 Dentine Exposure Series

In the series of dentitions showing the progression of dentine exposure in *P. lotor*, a clear trend can be observed in the advancement of the wear. Abrasive wear starts at the tips of the cusps, after which the areas spread along the crests, connecting the isolated sections of exposed dentine. In a next phase, the ridges of exposed dentine grow in width, until they cover the majority of the internal part of the teeth. The extent of dentine exposure differs per cusp on each tooth through this progression, however, and needs to be described individually.

**Figure 6:**
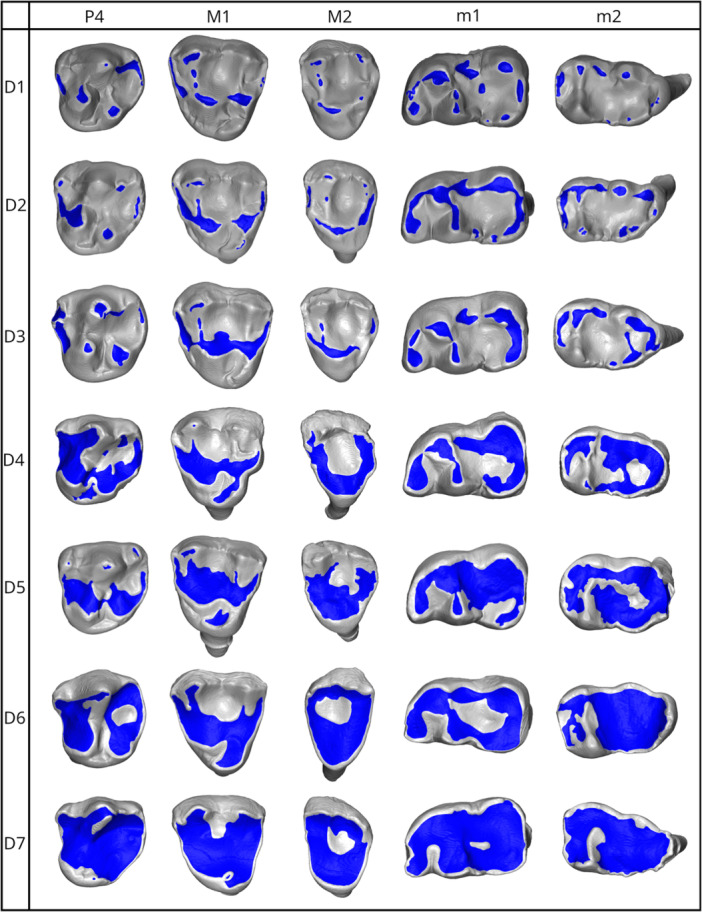
Dentine exposure series, with dentine in the colour blue and enamel in the colour grey. The column represents tooth position and the row represents the specimen. Right = distal, left = mesial, top = buccal, bottom = lingual. Teeth not to scale.

The dentine exposure on the P4 starts with the tips of the metacone, protocone, hypocone and the paracone, as can be observed on D1 (see Figure 6). The area of exposed dentine on the metacone is the largest of all the cusps and extends from halfway over the postcingulum, across the metacone and partly up the distal side of the paracone. There is a further area of exposed dentine on the precingulum near the parastyle. These areas grow in size throughout the dentine exposure series, with particularly the lingual cusps and the precingulum showing significant wear in many of the specimens. An exception is the metacone, which does not show any abrasion in some of the less abraded specimens, apart from the first one, but gets progressively more worn toward the end of the series. Abrasion of the basins of the tooth starts from the lingual cusps in specimen D4 and spreads buccally in subsequent specimens. The last area to be abraded is the lingual face of the paracone, where a crest extends towards the protocone.

On the M1 of specimen D1, the mesial side of the paracone shows exposed dentine, as well as the tips of the protocone and the metaconule (Figure 6). Some sections of the internal crest between the paracone and the protocone and an elongated area along the precingulum show signs of abrasion. Some abrasion can likewise be observed on the postcingulum. These areas of dentine exposure are conserved in specimen D2, but are extended in size. The dentine exposure on the protocone is connected to that of the precingulum and the internal crista in this specimen and the areas of exposed dentine on the cusps are larger. Finally, there is a small area of abrasion on the tip of the hypocone. Abrasion of the basin starts in specimen D3, which shows a clear rounded area of exposed dentine at the centre of the internal basin of the tooth. Significant wear on the hypocone commences from D4 onwards, and the buccal cusps of the tooth remain relatively unworn throughout the entire series, with the exception of the most worn specimen D7. In this specimen, the only remaining unworn area is a v‐shaped area between the buccal cusps.

On the M2 of D1, the tips of the metacone, metaconule and the protocone are lightly abraded (Figure 6). Wear on the paracone is limited to the mesiolingual side of the cone. Along the crest between the paracone and the protocone there is some further evidence of abrasion. In specimen D2, the area of exposed dentine on the protocone is elongated mesially and distally and the wear on the metaconule has spread to the postcingulum. There is an additional abraded surface along the precingulum. In subsequent specimens, the abrasion on the tips of the buccal cusps is less pronounced, whereas the dentine exposure of the protocone and connected crests is more extensive. In specimen D5, the internal basin of the tooth becomes abraded. In D6 and D7, most of the internal morphology of the tooth has been abraded, save for a roughly circular area at the centre of the basin.

The m1 of the lower dentition is initially abraded at the tips of the cusps (Figure 6). The first specimen shows exposed dentine on the tips of the paraconid, protoconid and metaconid, as well as on the crests between them, although these areas are not connected to each other. There are likewise signs of abrasion on the tips of the mesoconid, hypoconid, entoconid, and on the postcristid. Finally, there is a small abraded surface in the centre of the talonid basin. Specimen D2 shares all the sites of abrasion that were present in specimen D1, but nearly all of these are connected to each other in a continuous area of exposed dentine. In subsequent specimens, the dentine exposure grows in surface area from the hypoconid and the protoconid into the internal basins on the tooth. The deepest part of the talonid basin is the last area to be abraded.

The m2 of specimen D1 shows small abraded surfaces on the tips of the protoconid, hypoconid, entoconid, and the distal hypoconulid (Figure 6). Furthermore, only two crests show evidence of abrasional wear, which are the oblique cristid and the paracristid. The talonid basin of the m2 starts showing abrasion from D3 onwards. This specimen, as well as specimen D4, shows a narrow path of dentine exposure across the centre of the talonid basin that reaches from the hypoconid to the entoconid. Subsequent specimens show more homogeneous wear throughout the basin. Overall, the metaconid shows little abrasion throughout the dentine exposure series, and its tip remains unworn in the last specimen of the series. The distal faces of the metaconid and protoconid furthermore remain unworn in this specimen.

## 4 Discussion

### 4.1 A Second Phase of the Power Stroke

*P. lotor* has two phases of movement during the power stroke of its mastication cycle (see Figures 3, 4, 5). During the first phase of the power stroke, the buccal cusps of the upper dentition overlap those of the lower dentition and make contact with the buccal sides of the lower molars. Meanwhile, the hypocone of the P4 occludes into the trigonid and the M1 protocone occludes with the talonid basin of the m1. The metaconule of the M1 furthermore occludes into the trigonid of the m2 and the protocone of the M2 occludes into the talonid basin.

After centric occlusion, the lower teeth move lingually. When this happens, the protocones of the M1 and M2 collide with the hypoconids of the m1 and m2, respectively, and the protoconids of the lower molars come into contact with the hypocone of the P4 and the metaconule of the M1. When contact between these structures is procured, the mandible lowers, grinding the cusps against each other. During this movement no contact is made between opposing shearing edges. The time frame in which these contact areas exist is Phase II of the power stroke.

### 4.2 Mastication Compass

The mastication compass gives a quantitative representation of the mastication cycle of an animal in a two‐dimensional format (von Koenigswald et al. 2013). The power stroke consists of at least one phase, but may in some animals be accompanied by a second phase (von Koenigswald et al. 2013). The movement of the lower jaw during the first phase of the power stroke may include upwards or horizontal components, while the second phase of the power stroke, when present, might be either directed upwards or downwards. In *P. lotor*, the contact areas in the OFA clearly show a second phase of the power stroke, which is represented by a dotted line in the mastication compass (see Figure 5). This Phase II is directed downward, which is regularly the case for omnivores and insectivores (see e.g., the eulipotyphlans, primates and *Sus scrofa* in von Koenigswald et al. (2013)). The second phase of the power stroke of several specialised herbivores, in contrast, is directed upwards. This distinction in the inclination of the Phase II between omnivores/insectivores and specialised herbivores is owed to the guiding function of the cusps during occlusion (Bhullar et al. 2019; von Koenigswald et al. 2013). In specialised herbivores, the cusps are flattened (abraded) and, therefore, the lower jaw can freely move lingually at an incline during the second phase of the power stroke in these animals (von Koenigswald et al. 2013). In contrast, the cusps of omnivores and insectivores are higher and, therefore, when the cusps of the mandibular teeth come into contact with the lingual cusps of the maxillary teeth, these cusps guide the lower jaw downwards.

What is furthermore noteworthy is that both the first and second phase of the power stroke of the raccoon are oriented in a predominantly lingual direction. This direction of jaw movement has been associated with a grinding function (von Koenigswald et al. 2013; Mills 1967).

Finally, both Phases I and II of the power stroke of the raccoon have a very small vertical component. Carnivorans, which ancestrally have enlarged shearing blades on their carnassial teeth (upper fourth premolar and lower first molar) (Van Valkenburgh 2007), generally perform a steep jaw movement during the power stroke with a mostly orthal and only slightly lingual inclination (Lang and Martin 2024). This vertical movement is needed to support the efficacy of the shearing blades that are present on the dentition of carnivores to shear flesh, as is evident in the hypercarnivores like *Felis silvestris*.

### 4.3 Function During the First Phase of the Power Stroke

#### 4.3.1 Shearing

In general, the shearing features of the dentition of the raccoon are quite reduced. The cusps are short and blunted, the molars are rounded and the carnassial shearing blades of the P4 metacrista and the m1 paracristid are reduced (Ahrens 2012). Still, there are some wear facets on the dentition of *P. lotor* that are not described in the tribosphenic molar.

Raccoons have 14 facets on their upper first molar (see Figure 2). Seven of these correspond to the original facets that were described by Crompton (1971) on the tribosphenic molar. Two additional facets on the buccal sides of the metaconule and protocone are not associated with the first phase of the power stroke. The remaining five facets (M1‐MTCL‐mb, M1‐MTCL‐ml, M1‐MTCL‐d, M1‐POCNG‐d and M1‐HY‐mb) are associated with the hypocone and the metaconule of the M1; cusps that are either absent or relatively functionally unimportant in the tribosphenic molar.

Structures that are newly evolved in the dentition of mammals often bear shearing facets. Even though shearing might not be the primary function of these structures, they could compensate for the loss of shearing facets that occurs due to the development of these novelties, such as is the case in primates (Kay and Hiiemae 1974). In a similar way, the shearing function of the metaconule and hypocone on the upper dentition of *P. lotor* is likely a way to compensate for the reduction of the carnassial shearing blades and cutting edges in these teeth. However, the primary function of these newly evolved cusps is most probably different.

#### 4.3.2 Crushing Basins

Mammals crush food by pressing the crushing cusps into the basin of an occluding tooth (Ungar 2010). It is, therefore, the tips and the sides of the cusp, as well as the sides and floor of the opposing basin, that are involved in the crushing action, rather than crests or shearing edges of the cusp (Kay and Hiiemae 1974). Crushing structures are furthermore aligned roughly parallel to the occlusal plane, as the force exerted during crushing is perpendicular to this plane, and the walls of a crushing basin are usually inclined towards the deepest point of the basin. This is to trap the food item in the basin and to generate an increase of the force exerted on the food item as the basin gets narrower towards the base.

**Figure 7:**
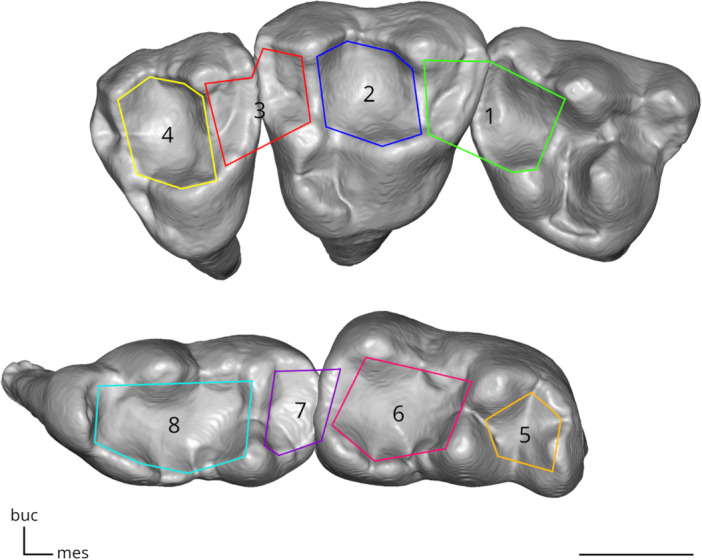
Overview of the crushing basins present on the upper molars and fourth premolar and the lower molars. The basins are numbered from 1 to 8 from mesial to distal starting with the upper dentition and ending with the lower dentition. The scale bar indicates 5 mm.

The teeth of *P. lotor* comprise several regions in which these characters are present (see Figure 7). On the lower teeth, the talonid basins of the m1 and m2 (numbers 6 and 8 in Figure 7) act as crushing basins with the opposing protocones of the M1 and M2 as the crushing structures that occlude into these basins. The interaction between protocone and talonid basin is a plesiomorphy stemming back from the tribosphenic molar pattern (Crompton 1971).

The crushing action between these structures is exemplified by the wear that was observed in the dentine exposure series. The protocones of the M1 and M2 bear some of the most expansive areas of dentine exposure. By specimen D2, the tip of the M1 protocone has already completely eroded away and in specimen D4, the protocone of the M2 follows. In comparison, the para‐ and metacones on the buccal sides of these teeth, which are not associated with an opposing crushing basin, remain relatively intact throughout the dentine exposure series. Moreover, there is evidence of wear in the occluding basins of the lower teeth, which implies a crushing relationship between the upper protocones and the lower talonid basins. In specimen D1, there is a small surface of exposed dentine in the talonid basin of the m1, which could plausibly have been formed by its collision with the tip of the M1 protocone, or otherwise by food particles that were trapped between both surfaces. Specimen D3 furthermore shows a path of exposed dentine leading from the base of the entoconid of the m2 across the talonid basin to the tip of the hypoconid. This follows the path of the occluding M2 protocone during the power stroke, as is evident from the OFA analysis. This pattern of wear is also present in specimen D4. From the fifth specimen of the dentine exposure series onward, much of the internal enamel of the talonid basins has been worn down.

Next to the talonid crushing basins on the m1 and m2, raccoons have developed other crushing basins on their lower teeth. On the first lower molar, the space in the trigonid is walled by tall cusps and crests, providing a barrier for any food that gets trapped between them (number 5 in Figure 7). Three facets were observed on the internally‐facing sides of each of these cusps, showing that there is an opposing structure that occludes with this basin, contrary to the occlusion pattern of the tribosphenic molar (see Crompton (1971)). The OFA analysis confirms that the hypocone of the P4 occludes with this basin. In the dentine exposure series, it is clear that the P4 hypocone of *P. lotor* wears fast, similarly to the protocones of the upper teeth. This suggests a possible crushing function of this cusp. However, the trigonid is narrow and its base is not flattened like the talonid basin, since the surrounding cusps are relatively close together and are steeply inclined towards the floor of the trigonid. The internal surface of the trigonid is furthermore preserved throughout the dentine exposure series, including in specimen D7. It is, therefore, conceivable that the interaction between the P4 hypocone and the trigonid of the m1 has a different function rather than crushing, such as grinding, which is discussed later in this paper. The steeply‐sloped cusps of the trigonid can alternatively be explained phylogenetically, rather than functionally. Since the trigonid ancestrally made up the shearing blade on this tooth (the m1 is a carnassial tooth in Carnivora) (Van Valkenburgh 2007), it is possible that the inclination of these cusps in *P. lotor* is a plesiomorphy that derives from the carnivoran ground pattern.

Between the postcristid along the distal edge of the m1 and the protocristid of the m2, there is a surface that is parallel to the occlusal plane (number 7 in Figure 7). This basin replaces the paraconid, which is absent in the m2 of *P. lotor*. The OFA analysis shows that the metaconule of the M1 occludes with this basin. The tip of this cusp is already worn down in D1 and, like the protocone of the M1, shows much wear throughout the rest of the series. The mesial basin on the m2 likewise shows signs of wear. In specimen D1, the postcristid of the m1 is worn down, as well as the paracristid on the m2 (see Figure 6). Subsequent specimens show wear on the same areas and from the third specimen of the dentine exposure series onward, the wear extends further into the basin on the m2. The abrasive wear on the two opposing structures suggests that there is an occlusal relationship between them and since the necessary features for crushing (a flat area roughly parallel to the occlusion plane, surrounding cusps that incline into the basin and an opposing structure that occludes into the basin) are in place, it is plausible that the basin on the mesial side of the m2 is an additional site for crushing. Seeing as the metaconule of the M1 occludes into this basin, it is likely that the paraconid reduced together with the development of the large metaconule on the M1.

In addition to the crushing basins formed by the lower dentition, there are some indications for potential crushing basins on the upper dentition of *P. lotor* as well. The upper molars are effectively divided into four basins. The first of these spans between the crest between the P4 paracone and hypocone until the internal crest between the M1 paracone and paraconule (number 1 in Figure 7), next to which there is the basin between the two internal crests of the first upper molar (number 2 in Figure 7). More distal in the tooth row there is a basin between the internal crest between the metacone and metaconule of the M1 and the internal crest between the paracone and paraconule on the M2 (number 3 in Figure 7). Finally, there is a basin internally on the M2, between the two internal crests of this tooth (number 4 in Figure 7). In the first specimen of the dentine exposure series, there is some wear along the postcingulum of the P4 and the precingulum of the M1, and this wear is present throughout the series. Localised wear in the centre of the internal basin on the M1 is most obvious in specimen D3. In this specimen, there is a clear circular‐shaped area of exposed dentine in the centre of the basin that cannot be attributed to wear of the protocone on the M1, as the area lies outside of the extent of this cusp. The wear is likely caused by the pressure of the occluding m1 hypoconid. Wear in this basin continues in the subsequent specimens. The third basin that spans across the distal side of the M1 and the mesial side of the M2 starts showing signs of abrasion from the second specimen of the dentine exposure series onward. Up until specimen D3, the wear is limited mostly to the postcingulum of the M1 and the precingulum of the M2, which make up the centralmost portion of the basin, after which the exposed dentine extends further to either side towards the crests that limit this basin. Finally, the basin on the M2 that spans between the two internal crests between the buccal cusps and their associated conules starts showing wear from D4 onwards. The worn area in this case expands from the protocone of the M2 into the basin, although never reaching all the way across to its deepest point: on specimens D6 and D7, the two most worn specimens in the series, there is an unworn surface of enamel in the centre of the basin on the M2.

The opposing cusps that occlude with these basins are, from mesial to distal, the protoconid of the m1, the hypoconid of the m1, the protoconid of the m2 and the hypoconid of the m2. In the dentine exposure series, these cusps appear to be some of the structures that are worn the fastest, as can be inferred from specimen D1, where the protoconids and hypoconids of the lower molars are the most worn structures on each respective tooth, save for the mesoconid of the m1 and the homologous structure on the m2. In specimen D4, the large extent of the wear on the hypoconids of the lower molars is especially apparent, and the protoconid of the m2 is also highly worn compared to other structures in this specimen.

### 4.4 Function During the Second Phase of the Power Stroke

#### 4.4.1 The P4 Hypocone as an Additional Grinding Structure

Most notable in the morphology of the tooth crown of the upper fourth premolar of *P. lotor* is a large hypocone to the distal side of the protocone. According to the results of the OFA analysis that was performed in this study, this hypocone occludes into the trigonid of the m1. This finding is corroborated by the facets that were found on the sides of the paraconid (m1‐pa‐d), protoconid (m1‐pr‐ml) and metaconid (m1‐me‐mb) of the m1 that face internally into the trigonid, as well as their antagonistic facets on the hypocone of the P4: P4‐HY‐mb, P4‐HY‐b and P4‐HY‐dl.

Hypocones have evolved convergently more than twenty different times in the therian clade (Hunter and Jernvall 1995). The additional lingual cone appears especially frequently in herbivores, having evolved in artiodactyls, perissodactyls and proboscideans, amongst others. However, these hypocones cannot be compared to that of the raccoon, given their distinct function. The tooth crowns of most herbivores are specialised and fully adapted to processing plant material. A series of enamel ridges that become exposed when the enamel on the cusps is abraded are ground together to finely process the fibrous plant foods of their diet before digestion (Ungar 2010; Winkler et al. 2020). Since their tooth crowns are primarily flat, the hypocone, in their case, functions as a site for widening, and, therefore, increasing the efficiency, of the tooth as a whole. It does not have an individual function, however, as the teeth of herbivores are specialised to work as one functional unit in breaking up plant matter.

Omnivores, in contrast, use the hypocone as an additional crushing structure besides the protocone and it is likewise involved in grinding between the hypocone and the protoconid of the m1 (Berkovitz and Shellis 2018). In carnivorans, hypocones are relatively rare, as the members of this group generally have dentitions that are specialised for shearing meat using the blades on the paracristid on the m1 and the metacrista of the P4 (Van Valkenburgh 2007; Ungar 2010). The hypocone, being positioned distal to the protocone, which originally defined the interdental space where the two blades shear past each other, obstructs this path and prevents efficient shearing between the carnassial blades. Instead, the hypocone broadens the tooth and occludes with the trigonid of the first mandibular molar. This effectively increases the crushing potential of the tooth, as noted by Hunter and Jernvall (1995). Animals in the carnivoran clade that are strict carnivores thus lack a hypocone, and this cusp is only found in species that have an omnivorous or herbivorous lifestyle, like some members among the ursids, mustelids and procyonids.

With *P. lotor* being a generalist, the structures on its tooth crown have varying functions and their morphology is more reminiscent of the classic tribosphenic pattern than those of specialised herbivores. In this way, its hypocone is more similar to that found in primates. While ancestrally primates had teeth that were adapted mainly for shearing, as is evident from the dentition of early Eocene *Tetonoides*, primates gradually developed dentitions with special adaptations for the crushing and grinding of food (Kay 1977). One of these adaptations was a hypocone, which appears first in the lineage leading up to the first catarrhines. Like in raccoons, the hypocone of primates occludes with the trigonid, performing a crushing function. The buccal sides of the protocone and hypocone of primates, furthermore each support a facet that is inconsistent with the contact areas that exist in Phase I of the power stroke. These facets instead originate during Phase II of the power stroke, when the lower dentition moves lingually on the working side of the jaw. During this phase, the protocone comes into contact with the hypoconid of the opposing tooth and the hypocone with the hypoconulid or hypoconid of the opposing and the protoconid of the succeeding tooth. These facets are described by Kay (1977) as having been produced due to the grinding function of the associated cusps in primates. The hypocone, in these animals, has a double function as a crushing and grinding device.

Analogous facets can be found on the protocone and metaconule on the M1 in *P. lotor*, as well as the hypocone of the P4 and the protocone of the M2. These facets have in common that they do not have boundaries towards the base of the cusp that are as clearly defined as the other facets on the dentition of *P. lotor*. A possible explanation for this is that like in primates, these facets are the result of grinding rather than shearing, and, therefore, particles are trapped between the occluding structures, partially obstructing tooth‐tooth contact (Rensberger 1978).

#### 4.4.2 The M1 Metaconule as a Functional Substitute for the Hypocone

Located distally to the protocone on the M1 of *P. lotor*, where the hypocone is situated on the P4, there is an enlarged metaconule. This is one of the four large cusps on this tooth, next to the protocone, paracone and metacone. The hypocone at this tooth position is shifted lingually and reduced in size significantly compared to the homologous cusp on the P4. This organisation of the tooth crown of the M1 indicates that the metaconule might fulfil the function of the hypocone in this tooth.

One argument that supports this observation comes from the OFA analysis. This shows that the metaconule of the M1 occludes with the (incomplete) trigonid of the second lower molar. On the P4, however, it is the hypocone that occludes with the m1 trigonid. When comparing the facets present on the M1 metaconule and P4 hypocone (Figure 2), it can be observed that each facet on the M1 metaconule was formed through contact with opposing structures that are homologous to those that formed the facets on the P4 hypocone.

The buccal facet on the M1 metaconule can be compared to the buccal facet on the P4 hypocone and those of the protocones of the upper molars. This facet only comes into contact with the opposing tooth during the second phase of the power stroke, when the lower dentition moves downward and the m2 protoconid moves along the M1 metaconule from its base to its tip. As in the case of the P4 hypocone, this facet is a result of grinding, unlike the other facets on this cusp, which are a result of shearing.

Besides this, the metaconule of the M1 is likely involved in crushing as well. Like the protocone of the M1, the metaconule gets worn fast during occlusion, as is evident from the dentine exposure series. Furthermore, as mentioned before in the section on the crushing basins found in *P. lotor*, the trigonid basin of the m2, which occludes with the M1 metaconule, likewise shows clear signs of abrasion through the dentine exposure series. This shows that these structures have an occlusal relationship with each other and are involved in crushing. Seeing, therefore, that the metaconule is involved in the same food processing functions as the hypocone, and furthermore occupies the same spatial position on the M1 which is occupied by the hypocone on the P4, it seems likely that the metaconule serves as a hypocone in the M1 of raccoons.

The idea that the metaconule on the M1 of *P. lotor* functionally replaced the hypocone was mentioned before in the phylogenetic analysis of Procyonidae by Decker and Wozencraft (1991). However, this hypothesis was rejected by these authors, as they argued that the large lingual cusp that is distal to the protocone on the M1 is a hypocone that was misidentified as a metaconule. In their phylogeny, *Nasua* and *Procyon* are placed together as sister taxa, as they share many dental characters. They characterise *Procyon cancrivorus*, a South and Central American raccoon species, as having a large hypocone and reduced metaconule on the M1. Therefore, if the enlarged metaconule on the M1 were a synapomorphy between *Nasua* and *Procyon*, they argue that the metaconule would have had to increase in size and functionally replace the hypocone in these genera, after which it got secondarily reduced, and the hypocone got enlarged again in *P. cancrivorus*.

Both before and since that publication, several other phylogenies of Procyonidae have identified the distolingual cusp of the upper M1 of all species within *Nasua*, *Nasuella* and *Procyon* as the metaconule—not the hypocone (Ahrens 2012; Baskin 1982, 2004). However, the functional implications of the identification for these cusps have not been re‐examined and the probability that the metaconule functionally replaced the hypocone on the M1 still stands.

In fact, a metaconule with the functional role of a hypocone is hardly rare. The term “hypocone” is by definition a functional term rather than a term relating to the homologous origin of the cusp (Hunter and Jernvall 1995). Hypocones in therians have interchangeably originated from the postprotocingulum (these are considered “true” hypocones) or the metaconule, and in some mammalian taxa, the cusp is even derived from the meta‐ or protocone. Extant mammals that have an enlarged metaconule distal to the protocone on the first molar in the stead of a hypocone include the Diprotodontia, some primates and, closest to raccoons, the ursids (Anemone et al. 2012; Hunter and Jernvall 1995; Jiangzuo et al. 2019; Tedford and Woodburne 1998).

Of all the taxa, extinct and extant, described by Hunter and Jernvall (1995) to possess such a “pseudohypocone”, hardly any show a secondary replacement of the hypocone by the metaconule. In these taxa, a “true” hypocone never evolved, and the metaconule is the original cusp to function as a hypocone (Chornogubsky et al. 2009; Goin 2023; Ladevèze et al. 2010; Luccisano et al. 2020; Prothero and Foss 2007; Tedford and Woodburne 1998; Travouillon et al. 2014). In *P. lotor* this is not the case, since the presence of a hypocone is the ancestral state for the Procyonidae (Ahrens 2012). The secondary reduction and replacement of the hypocone by the metaconule on the M1 is, therefore, noteworthy. The only other taxon in which this could conceivably have occurred is Artiodactyla. Eocene artiodactyls show the development of a distolingual hypocone, originating from the postprotocingulum on the upper molars, as in dichobunids (Luccisano et al. 2020; Prothero and Foss 2007). Subsequently, the molars became increasingly bunoselenodont, lacking a hypocone, approaching the four‐cusped selenodont dentitions of many modern artiodactyls, in which the metaconule forms the distolingual cusp (Hunter and Jernvall 1995; Luccisano et al. 2020; Prothero and Foss 2007).

In *Procyon gibsoni*, an extinct species of raccoon from the early Pleistocene of Florida, the M1 metaconule is quite reduced in size (Emmert and Short 2018). The internal conular crista between the metacone and the metaconule is furthermore interrupted and the crest between the paracone and hypocone on the P4 is lacking, which makes the previously‐discussed crushing basins that are clearly apparent in *P. lotor* less distinguishable in *P. gibsoni*. *P. megalokolos*, from the same time and locality, however, does show a well‐developed M1 metaconule and associated crest (Emmert and Short 2018), as does an earlier *Procyon* finding from the Pliocene of Texas, which has since tentatively been synonymized with *P. lotor* (Emmert and Short 2018; Hibbard 1941). This implies that the condition present in *P. lotor* is plesiomorphic for the genus and the reduction of the metaconule in *P. gibsoni* is apomorphic for the species.

Indeed, it is agreed between the morphologically‐based phylogenies of Baskin (2004) and Ahrens (2012) that the reduction of the hypocone and the enlargement and repositioning of the metaconule on the M1 of *P. lotor* is a phenomenon that evolved in the lineage including *Paranasua, Nasua, Nasuella* and *Procyon*. This supports the idea of a functional replacement of the hypocone by the metaconule in this lineage. While the functional relationships of the cusps of coati dentition have yet to be studied, the morphology of the tooth crown of these mammals does appear to point to this conclusion. However, whether this organisation of the tooth crown is evolutionarily homologous between the coatis and the raccoons depends on the type of data that is used in the phylogenetic analysis. While raccoons and coatis are grouped together in morphologically‐based studies, molecular studies place them in two separate clades, and group them with species that show a completely different tooth morphology (raccoons are placed with *Bassariscus* and coatis with *Bassaricyon*) (Ahrens 2012; Baskin 2004; Decker and Wozencraft 1991; Eizirik et al. 2010; Forasiepi et al. 2014; Fulton and Strobeck 2007; Hassanin et al. 2021; Koepfli et al. 2007). Further evidence for the latter hypothesis comes from the earliest described *Procyon* species from the Late Miocene of California, *P. garberi*, which shows the morphological similarity between the m1 of *Procyon* and *Bassariscus* (Wagner and Wang 2025). The explanation that these studies give for the morphological similarity of phylogenetically distant raccoons and coatis is their similar lifestyle. It is possible that raccoons and coatis convergently developed the metaconule and reduced the hypocone as an adaptation to their ecology, similarly to what can be observed with *Ailurus*.

According to the phylogenetic analysis of Ahrens (2012), the presence of a (“true”) hypocone on the M1 is a plesiomorphic character for procyonids. The metaconule, therefore, did not initially develop in the place of a hypocone, as is the case in many other mammal taxa (Hunter and Jernvall 1995), but functionally replaced it during the course of procyonid evolution. One explanation for the co‐occurrence of the two cusps in *P. lotor* is that the reduced and more lingually‐positioned M1 hypocone grants *P. lotor* a functional advantage, since the hypocone still performs a shearing function, as demonstrated by the wear facet analysis and OFA analysis. The retaining of the hypocone as well as an enlarged metaconule broadens the tooth surface and increases the number of attritional contacts between the upper and lower teeth, which assists the fragmentation of food between the molars. Alternatively, with ongoing evolution of *P. lotor*, the hypocone may become completely reduced, like is the case for *Nasua* and *Nasuella*. Since the tooth crown of the M1 of *P. lotor* is quite comparable to those of coatis, *P. lotor* may serve as a morphofunctional analogue to a hypothetical ancestral condition of coatis where both the metaconule and the hypocone are still in use, before the hypocone is fully reduced. Nonetheless, the reality of such an ancestor is difficult to confirm due to a lack of fossil data of the genus (Dalquest 1978; Emmert and Short 2018; Prevosti and Forasiepi 2018), as well as conflicting systematics within the entire family (Ahrens 2012; Baskin 1982, 2004; Decker and Wozencraft 1991; Eizirik et al. 2010; Forasiepi et al. 2014; Fulton and Strobeck 2007; Hassanin et al. 2021; Koepfli et al. 2007).

## 5 Conclusions

The lifestyle and diet of *P. lotor* are reflected in its dental adaptations. Its cheek teeth are rounded with blunted cusps, and its carnassial blades are reduced, implying the diminution of the shearing function of its dentition. The P4 furthermore shows the development of a large hypocone—a synapomorphy of the Procyonidae. This hypocone is positioned directly distal to the protocone of this tooth and is involved in crushing and grinding. The organisation of the upper and lower cheek teeth of *P. lotor* into compact, steep‐walled crushing chambers confirms this conclusion. The M1 is in possession of both a large distolingual metaconule that is mesio‐distally aligned with the protocone, and a lingually‐shifted hypocone that is reduced in size relative to the one on the P4. The occlusal relationships, positioning and wear patterns of these cusps imply a functional replacement of the M1 hypocone by the metaconule. This organisation might pose an adaptive advantage to the reduction of food items, in which case the role of the reduced hypocone must be further investigated using, for example, microwear analysis. However, the more likely scenario is that *P. lotor* shows an intermediate stage towards complete reduction of the M1 hypocone and its functional replacement by the metaconule, such as is the case in closely related and ecologically similar *Nasua*. The evolutionary trajectory of the metaconule in the family of Procyonidae can nevertheless only be properly investigated once the phylogeny of the family is resolved.

## Author Contributions

**Sophie E. Koomen:** conceptualisation, data curation, resources, formal analysis, investigation, methodology, writing – original draft, writing – review and editing. **Andreas J. Lang:** conceptualisation, data curation, methodology, supervision, writing – review and editing. **Thomas Martin:** conceptualisation, resources, supervision, writing – review and editing.

## Funding

The authors have nothing to report.

## Ethics Statement

The authors have nothing to report.

## Conflicts of Interest

The authors declare no conflicts of interest.

## Supporting information

Supporting File:

## Data Availability

The data that support the findings of this study are openly available in MorphoMuseum at https://doi.org/10.18563/journal.m3.286.
