# Supplementary material for: Tooth Function of the Northern Raccoon (Procyon lotor) and Adaptations to Omnivory in the Order Carnivora
Source: J Morphol. 2026 Jul 3;287(7):e70142. doi: 10.1002/jmor.70142 (PMC13329977; doi:10.1002/jmor.70142)
Supplement: Supplementary file 1 — Supporting File: [file JMOR-287-e70142-s001.docx]

**Supplementary Data**

**Table 1.** Specimens used for each analysis. ZFMK = Zoologisches Forschungsmuseum Koenig. MAM = mammal department. For specimens 2017-0575, 2017-0578 and 2017-0580 only the year of addition to the collection is known.

| **Collection Nr.** | **Collection Day** | **Collection Month** | **Collection Year** | **Locality** | **Country** | **Collector** | **Gender** |
| --- | --- | --- | --- | --- | --- | --- | --- |
| **MAM1993-0289** |  |  | 1981 |  | United States | Entzeroth | ? |
| **MAM2013-0341** |  |  | 2012 |  |  | Türsen | male |
| **MAM2016-0879** | 28 | 1 | 1977 | Hessen, Umgebung Edersee | Germany | Lutz, W. | female |
| **MAM2016-0881** | 6 | 2 | 1977 | Hessen, Umgebung Edersee | Germany | Lutz, W. | female |
| **MAM2016-0894** | 7 | 5 | 1977 | Hessen, Umgebung Edersee | Germany | Lutz, W. | male |
| **MAM2016-0897** | 15 | 5 | 1977 | Hessen, Umgebung Edersee | Germany | Lutz, W. | male |
| **MAM2016-0907** | 31 | 7 | 1977 | Hessen, Umgebung Edersee | Germany | Lutz, W. | male |
| **MAM2016-0912** |  |  | 1977 | Hessen, Umgebung Edersee | Germany | Lutz, W. | male |
| **MAM2016-0914** | 4 | 11 | 1977 | Hessen, Umgebung Edersee | Germany | Lutz, W. | female |
| **MAM2016-0923** | 8 | 3 | 1978 | Hessen, Umgebung Edersee | Germany | Lutz, W. | ? |
| **MAM2016-0928** | 1 | 3 | 1978 | Hessen, Umgebung Edersee | Germany | Lutz, W. | female |
| **MAM2016-0932** | 18 | 4 | 1978 | Hessen, Umgebung Edersee | Germany | Lutz, W. | male |
| **MAM2016-0933** | 6 | 5 | 1978 | Hessen, Umgebung Edersee | Germany | Lutz, W. | male |
| **MAM2016-0935** | 21 | 5 | 1978 | Hessen, Umgebung Edersee | Germany | Lutz, W. | female |
| **MAM2016-0936** | 4 | 6 | 1978 | Hessen, Umgebung Edersee | Germany | Lutz, W. | male |
| **MAM2016-0937** | 7 | 6 | 1978 | Hessen, Umgebung Edersee | Germany | Lutz, W. | ? |
| **MAM2016-0938** | 18 | 6 | 1978 | Hessen, Umgebung Edersee | Germany | Lutz, W. | ? |
| **MAM2016-0939** | 15 | 6 | 1978 | Hessen, Umgebung Edersee | Germany | Lutz, W. | female |
| **MAM2016-0945** | 21 | 9 | 1978 | Hessen, Umgebung Edersee | Germany | Lutz, W. | ? |
| **MAM2016-0966** |  |  | 1977 | Hessen, Umgebung Edersee | Germany | Lutz, W. | ? |
| **MAM2016-0967** |  |  | 1977 | Hessen, Umgebung Edersee | Germany | Lutz, W. | male |
| **MAM2016-0968** |  |  | 1977 | Hessen, Umgebung Edersee | Germany | Lutz, W. | ? |
| **MAM2016-0969** |  |  | 1977 | Hessen, Umgebung Edersee | Germany | Lutz, W. | ? |
| **MAM2017-0575** | ded. III.2017 | | | Nordrhein-Westfalen, Rheinland | Germany | Gey; ded. Dr. W. Lutz | ? |
| **MAM2017-0578** | ded. III.2017 | | | Nordrhein-Westfalen, Rheinland | Germany | Gey; ded. Dr. W. Lutz | male |
| **MAM2017-0580** | ded. III.2017 | | | Nordrhein-Westfalen, Rheinland | Germany | Gey; ded. Dr. W. Lutz | male |

**Table 2.** Area of OFA contact points on the lower m1 per time step. Area is given in mm^2^.

| Timestep | Facet | Triangles | Area |
| --- | --- | --- | --- |
| 53 | en-dl | 54 | 0.134052 |
| 54 | en-dl | 119 | 0.278967 |
| 55 | en-dl | 139 | 0.32772 |
| 56 | en-dl | 160 | 0.384365 |
| 57 | en-dl | 157 | 0.379244 |
| 58 | en-dl | 170 | 0.40759 |
| 59 | en-dl | 176 | 0.421274 |
| 60 | en-dl | 175 | 0.420773 |
| 61 | en-dl | 158 | 0.39286 |
| 62 | en-dl | 158 | 0.39286 |
| 63 | en-dl | 147 | 0.374909 |
| 64 | en-dl | 120 | 0.334457 |
| 65 | en-dl | 112 | 0.315488 |
| 66 | en-dl | 85 | 0.249547 |
| 67 | en-dl | 85 | 0.249547 |
| 68 | en-dl | 59 | 0.177848 |
| 69 | en-dl | 59 | 0.177848 |
| 70 | en-dl | 53 | 0.121757 |
| 71 | en-dl | 52 | 0.115099 |
| 72 | en-dl | 74 | 0.174073 |
| 73 | en-dl | 72 | 0.172893 |
| 74 | en-dl | 74 | 0.183307 |
| 75 | en-dl | 100 | 0.242105 |
| 76 | en-dl | 128 | 0.334587 |
| 77 | en-dl | 133 | 0.345982 |
| 78 | en-dl | 174 | 0.44369 |
| 79 | en-dl | 174 | 0.44369 |
| 80 | en-dl | 102 | 0.257681 |
| 81 | en-dl | 102 | 0.257681 |
| 82 | en-dl | 107 | 0.270064 |
| 83 | en-dl | 87 | 0.214459 |
| 84 | en-dl | 94 | 0.229849 |
| 87 | en-dl | 7 | 0.019669 |
| 88 | en-dl | 42 | 0.114039 |
| 89 | en-dl | 44 | 0.117838 |
| 38 | en-mb | 5 | 0.008645 |
| 39 | en-mb | 9 | 0.01625 |
| 41 | en-mb | 6 | 0.011068 |
| 42 | en-mb | 132 | 0.318076 |
| 43 | en-mb | 142 | 0.337071 |
| 45 | en-mb | 103 | 0.268359 |
| 46 | en-mb | 172 | 0.455355 |
| 47 | en-mb | 96 | 0.277621 |
| 48 | en-mb | 214 | 0.680818 |
| 49 | en-mb | 42 | 0.112553 |
| 50 | en-mb | 175 | 0.562411 |
| 51 | en-mb | 343 | 1.12433 |
| 52 | en-mb | 101 | 0.305055 |
| 53 | en-mb | 277 | 0.94944 |
| 54 | en-mb | 497 | 1.65109 |
| 55 | en-mb | 204 | 0.731645 |
| 56 | en-mb | 255 | 0.93932 |
| 57 | en-mb | 454 | 1.61843 |
| 58 | en-mb | 499 | 1.74568 |
| 59 | en-mb | 511 | 1.78141 |
| 60 | en-mb | 509 | 1.77748 |
| 61 | en-mb | 470 | 1.66937 |
| 62 | en-mb | 470 | 1.66937 |
| 63 | en-mb | 486 | 1.73388 |
| 64 | en-mb | 430 | 1.57062 |
| 65 | en-mb | 443 | 1.62529 |
| 66 | en-mb | 477 | 1.83325 |
| 67 | en-mb | 475 | 1.79376 |
| 68 | en-mb | 432 | 1.59513 |
| 69 | en-mb | 432 | 1.59513 |
| 70 | en-mb | 478 | 1.80747 |
| 71 | en-mb | 485 | 1.8207 |
| 72 | en-mb | 475 | 1.82331 |
| 73 | en-mb | 473 | 1.81973 |
| 74 | en-mb | 480 | 1.86333 |
| 3 | hy-db | 46 | 0.112422 |
| 4 | hy-db | 34 | 0.076847 |
| 5 | hy-db | 44 | 0.111977 |
| 6 | hy-db | 53 | 0.13665 |
| 7 | hy-db | 32 | 0.075762 |
| 11 | hy-db | 14 | 0.039273 |
| 37 | hy-mb | 34 | 0.098856 |
| 38 | hy-mb | 50 | 0.145389 |
| 39 | hy-mb | 53 | 0.153252 |
| 41 | hy-mb | 36 | 0.106405 |
| 42 | hy-mb | 130 | 0.446984 |
| 44 | hy-mb | 18 | 0.061812 |
| 45 | hy-mb | 103 | 0.346806 |
| 47 | hy-mb | 141 | 0.51201 |
| 49 | hy-mb | 72 | 0.267966 |
| 52 | hy-mb | 71 | 0.267641 |
| 55 | hy-mb | 106 | 0.423554 |
| 56 | hy-mb | 182 | 0.685567 |
| 61 | hy-mb | 316 | 1.2395 |
| 62 | hy-mb | 316 | 1.2395 |
| 63 | hy-mb | 289 | 1.15203 |
| 64 | hy-mb | 183 | 0.734553 |
| 65 | hy-mb | 173 | 0.695962 |
| 66 | hy-mb | 162 | 0.682656 |
| 67 | hy-mb | 161 | 0.678089 |
| 68 | hy-mb | 129 | 0.542859 |
| 69 | hy-mb | 127 | 0.538212 |
| 70 | hy-mb | 129 | 0.547909 |
| 71 | hy-mb | 128 | 0.536894 |
| 72 | hy-mb | 64 | 0.262924 |
| 73 | hy-mb | 64 | 0.262924 |
| 74 | hy-mb | 65 | 0.274732 |
| 93 | hy-ml | 102 | 0.311727 |
| 94 | hy-ml | 285 | 0.959399 |
| 95 | hy-ml | 373 | 1.29176 |
| 96 | hy-ml | 421 | 1.52513 |
| 97 | hy-ml | 448 | 1.67043 |
| 98 | hy-ml | 495 | 1.84236 |
| 99 | hy-ml | 500 | 1.87184 |
| 100 | hy-ml | 420 | 1.57783 |
| 101 | hy-ml | 228 | 0.83264 |
| 102 | hy-ml | 276 | 1.01359 |
| 103 | hy-ml | 302 | 1.12258 |
| 104 | hy-ml | 266 | 0.972908 |
| 105 | hy-ml | 230 | 0.830412 |
| 106 | hy-ml | 233 | 0.848862 |
| 107 | hy-ml | 233 | 0.843902 |
| 108 | hy-ml | 106 | 0.375776 |
| 109 | hy-ml | 164 | 0.540602 |
| 110 | hy-ml | 190 | 0.629797 |
| 111 | hy-ml | 200 | 0.659522 |
| 112 | hy-ml | 62 | 0.173848 |
| 113 | hy-ml | 110 | 0.324737 |
| 114 | hy-ml | 136 | 0.415449 |
| 115 | hy-ml | 134 | 0.411388 |
| 116 | hy-ml | 96 | 0.268455 |
| 117 | hy-ml | 96 | 0.268455 |
| 118 | hy-ml | 100 | 0.279232 |
| 119 | hy-ml | 45 | 0.114217 |
| 120 | hy-ml | 45 | 0.114217 |
| 121 | hy-ml | 43 | 0.111226 |
| 5 | me-db | 9 | 0.018531 |
| 6 | me-db | 23 | 0.047924 |
| 7 | me-db | 51 | 0.111011 |
| 9 | me-db | 29 | 0.068048 |
| 10 | me-db | 51 | 0.115772 |
| 11 | me-db | 102 | 0.22855 |
| 12 | me-db | 111 | 0.254714 |
| 13 | me-db | 123 | 0.283253 |
| 14 | me-db | 125 | 0.290934 |
| 15 | me-db | 127 | 0.295006 |
| 16 | me-db | 36 | 0.096466 |
| 17 | me-db | 89 | 0.220154 |
| 18 | me-db | 131 | 0.325952 |
| 19 | me-db | 144 | 0.352655 |
| 20 | me-db | 147 | 0.357055 |
| 21 | me-db | 92 | 0.228635 |
| 22 | me-db | 149 | 0.376941 |
| 23 | me-db | 210 | 0.535612 |
| 24 | me-db | 225 | 0.571274 |
| 25 | me-db | 108 | 0.283252 |
| 26 | me-db | 147 | 0.389211 |
| 27 | me-db | 199 | 0.521793 |
| 28 | me-db | 244 | 0.63912 |
| 29 | me-db | 299 | 0.780483 |
| 30 | me-db | 347 | 0.907335 |
| 31 | me-db | 356 | 0.931633 |
| 32 | me-db | 367 | 0.958299 |
| 33 | me-db | 347 | 0.908001 |
| 34 | me-db | 413 | 1.07368 |
| 35 | me-db | 214 | 0.5852 |
| 36 | me-db | 227 | 0.617663 |
| 37 | me-db | 285 | 0.767562 |
| 38 | me-db | 296 | 0.809897 |
| 39 | me-db | 296 | 0.809897 |
| 40 | me-db | 296 | 0.812718 |
| 41 | me-db | 337 | 0.943569 |
| 42 | me-db | 376 | 1.11434 |
| 43 | me-db | 380 | 1.14096 |
| 44 | me-db | 360 | 1.07685 |
| 45 | me-db | 392 | 1.25642 |
| 46 | me-db | 400 | 1.31724 |
| 47 | me-db | 219 | 0.724344 |
| 48 | me-db | 246 | 0.833875 |
| 49 | me-db | 259 | 0.950678 |
| 50 | me-db | 273 | 1.01618 |
| 51 | me-db | 293 | 1.10666 |
| 52 | me-db | 295 | 1.12362 |
| 53 | me-db | 301 | 1.20155 |
| 54 | me-db | 314 | 1.30471 |
| 55 | me-db | 328 | 1.3426 |
| 56 | me-db | 341 | 1.37771 |
| 66 | me-mb | 32 | 0.060581 |
| 67 | me-mb | 36 | 0.074382 |
| 68 | me-mb | 102 | 0.231307 |
| 69 | me-mb | 102 | 0.231307 |
| 70 | me-mb | 180 | 0.448116 |
| 71 | me-mb | 197 | 0.502153 |
| 72 | me-mb | 254 | 0.672775 |
| 73 | me-mb | 259 | 0.682282 |
| 74 | me-mb | 275 | 0.727304 |
| 75 | me-mb | 109 | 0.243841 |
| 76 | me-mb | 217 | 0.54439 |
| 77 | me-mb | 277 | 0.721487 |
| 78 | me-mb | 60 | 0.150725 |
| 79 | me-mb | 62 | 0.156678 |
| 80 | me-mb | 181 | 0.480282 |
| 81 | me-mb | 183 | 0.486816 |
| 82 | me-mb | 200 | 0.551314 |
| 84 | me-mb | 21 | 0.061532 |
| 85 | me-mb | 98 | 0.264547 |
| 86 | me-mb | 121 | 0.337794 |
| 13 | msc-db | 10 | 0.018185 |
| 14 | msc-db | 18 | 0.036312 |
| 15 | msc-db | 21 | 0.041737 |
| 18 | msc-db | 50 | 0.094994 |
| 19 | msc-db | 66 | 0.129815 |
| 20 | msc-db | 69 | 0.136663 |
| 22 | msc-db | 71 | 0.145068 |
| 23 | msc-db | 133 | 0.273001 |
| 24 | msc-db | 144 | 0.305601 |
| 26 | msc-db | 27 | 0.065147 |
| 27 | msc-db | 35 | 0.084589 |
| 28 | msc-db | 116 | 0.263017 |
| 29 | msc-db | 135 | 0.337024 |
| 30 | msc-db | 146 | 0.396846 |
| 31 | msc-db | 155 | 0.420951 |
| 32 | msc-db | 167 | 0.445596 |
| 33 | msc-db | 86 | 0.241998 |
| 34 | msc-db | 167 | 0.500315 |
| 37 | msc-db | 51 | 0.149704 |
| 38 | msc-db | 71 | 0.201476 |
| 39 | msc-db | 71 | 0.201476 |
| 40 | msc-db | 16 | 0.036324 |
| 41 | msc-db | 133 | 0.418558 |
| 42 | msc-db | 257 | 0.85433 |
| 43 | msc-db | 419 | 1.4268 |
| 44 | msc-db | 166 | 0.606013 |
| 45 | msc-db | 303 | 1.08458 |
| 46 | msc-db | 511 | 1.80767 |
| 47 | msc-db | 68 | 0.267202 |
| 48 | msc-db | 456 | 1.63364 |
| 49 | msc-db | 120 | 0.472503 |
| 50 | msc-db | 460 | 1.75004 |
| 51 | msc-db | 655 | 2.33871 |
| 52 | msc-db | 305 | 1.15884 |
| 53 | msc-db | 685 | 2.50177 |
| 54 | msc-db | 892 | 3.26566 |
| 55 | msc-db | 461 | 1.68185 |
| 56 | msc-db | 521 | 1.90099 |
| 57 | msc-db | 862 | 3.15651 |
| 58 | msc-db | 920 | 3.35049 |
| 59 | msc-db | 938 | 3.40852 |
| 60 | msc-db | 936 | 3.40516 |
| 61 | msc-db | 419 | 1.50924 |
| 62 | msc-db | 414 | 1.4838 |
| 63 | msc-db | 296 | 1.07976 |
| 64 | msc-db | 176 | 0.713033 |
| 65 | msc-db | 163 | 0.665255 |
| 66 | msc-db | 46 | 0.207338 |
| 67 | msc-db | 43 | 0.195659 |
| 75 | pa-d | 12 | 0.030682 |
| 76 | pa-d | 78 | 0.256004 |
| 77 | pa-d | 94 | 0.346796 |
| 78 | pa-d | 235 | 0.811501 |
| 79 | pa-d | 235 | 0.811501 |
| 80 | pa-d | 121 | 0.453929 |
| 81 | pa-d | 121 | 0.453929 |
| 82 | pa-d | 123 | 0.460575 |
| 83 | pa-d | 158 | 0.566167 |
| 84 | pa-d | 164 | 0.602308 |
| 88 | pa-d | 169 | 0.572902 |
| 89 | pa-d | 171 | 0.583284 |
| 70 | pa-m | 163 | 0.456688 |
| 71 | pa-m | 222 | 0.615498 |
| 72 | pa-m | 398 | 1.10307 |
| 73 | pa-m | 404 | 1.11649 |
| 74 | pa-m | 454 | 1.23126 |
| 76 | pa-m | 313 | 0.896148 |
| 77 | pa-m | 522 | 1.44398 |
| 80 | pa-m | 482 | 1.46649 |
| 81 | pa-m | 483 | 1.47037 |
| 82 | pa-m | 542 | 1.62962 |
| 84 | pa-m | 218 | 0.661128 |
| 85 | pa-m | 570 | 1.76277 |
| 86 | pa-m | 611 | 1.86443 |
| 87 | pa-m | 495 | 1.54104 |
| 90 | pa-m | 429 | 1.36218 |
| 91 | pa-m | 552 | 1.69 |
| 92 | pa-m | 482 | 1.48544 |
| 93 | pa-m | 316 | 0.995733 |
| 94 | pa-m | 249 | 0.79498 |
| 95 | pa-m | 205 | 0.673053 |
| 96 | pa-m | 167 | 0.52924 |
| 97 | pa-m | 129 | 0.412163 |
| 98 | pa-m | 61 | 0.176975 |
| 99 | pa-m | 51 | 0.157554 |
| 87 | pocd-m | 16 | 0.067676 |
| 91 | pocd-m | 24 | 0.116011 |
| 1 | pr-d | 71 | 0.218406 |
| 2 | pr-d | 233 | 0.706835 |
| 3 | pr-d | 340 | 1.10619 |
| 4 | pr-d | 278 | 0.920659 |
| 5 | pr-d | 296 | 0.976737 |
| 6 | pr-d | 308 | 1.01805 |
| 7 | pr-d | 265 | 0.918334 |
| 8 | pr-d | 145 | 0.531984 |
| 9 | pr-d | 185 | 0.654174 |
| 10 | pr-d | 184 | 0.65075 |
| 11 | pr-d | 244 | 0.861573 |
| 12 | pr-d | 220 | 0.797701 |
| 13 | pr-d | 225 | 0.814717 |
| 14 | pr-d | 227 | 0.818245 |
| 15 | pr-d | 229 | 0.826626 |
| 16 | pr-d | 132 | 0.514756 |
| 17 | pr-d | 162 | 0.631622 |
| 18 | pr-d | 190 | 0.729206 |
| 19 | pr-d | 197 | 0.756676 |
| 20 | pr-d | 197 | 0.756676 |
| 21 | pr-d | 157 | 0.573696 |
| 22 | pr-d | 194 | 0.74074 |
| 23 | pr-d | 250 | 0.915482 |
| 24 | pr-d | 254 | 0.927264 |
| 25 | pr-d | 150 | 0.487508 |
| 26 | pr-d | 186 | 0.659184 |
| 27 | pr-d | 215 | 0.70804 |
| 28 | pr-d | 243 | 0.822145 |
| 29 | pr-d | 263 | 0.864892 |
| 30 | pr-d | 284 | 0.911403 |
| 31 | pr-d | 292 | 0.934967 |
| 32 | pr-d | 293 | 0.943746 |
| 33 | pr-d | 248 | 0.751931 |
| 34 | pr-d | 300 | 0.940601 |
| 35 | pr-d | 147 | 0.432518 |
| 36 | pr-d | 164 | 0.493266 |
| 37 | pr-d | 192 | 0.580501 |
| 38 | pr-d | 202 | 0.616253 |
| 39 | pr-d | 202 | 0.616253 |
| 40 | pr-d | 167 | 0.495602 |
| 41 | pr-d | 233 | 0.734963 |
| 42 | pr-d | 291 | 0.957235 |
| 43 | pr-d | 294 | 0.968261 |
| 44 | pr-d | 263 | 0.835638 |
| 45 | pr-d | 316 | 1.05881 |
| 46 | pr-d | 352 | 1.18857 |
| 47 | pr-d | 165 | 0.502603 |
| 48 | pr-d | 210 | 0.718541 |
| 49 | pr-d | 211 | 0.70324 |
| 50 | pr-d | 278 | 0.967753 |
| 51 | pr-d | 370 | 1.28233 |
| 52 | pr-d | 383 | 1.3069 |
| 53 | pr-d | 475 | 1.61325 |
| 54 | pr-d | 538 | 1.83505 |
| 55 | pr-d | 596 | 2.02013 |
| 56 | pr-d | 629 | 2.11614 |
| 57 | pr-d | 901 | 3.24866 |
| 58 | pr-d | 904 | 3.23904 |
| 59 | pr-d | 904 | 3.23885 |
| 60 | pr-d | 908 | 3.25004 |
| 61 | pr-d | 793 | 2.79444 |
| 62 | pr-d | 790 | 2.78466 |
| 63 | pr-d | 688 | 2.43964 |
| 64 | pr-d | 534 | 1.89861 |
| 65 | pr-d | 491 | 1.78846 |
| 66 | pr-d | 327 | 1.24154 |
| 67 | pr-d | 322 | 1.21838 |
| 68 | pr-d | 259 | 1.0489 |
| 69 | pr-d | 259 | 1.0489 |
| 70 | pr-d | 177 | 0.799371 |
| 71 | pr-d | 165 | 0.770359 |
| 72 | pr-d | 108 | 0.593858 |
| 73 | pr-d | 108 | 0.593858 |
| 74 | pr-d | 102 | 0.551118 |
| 75 | pr-d | 125 | 0.651165 |
| 76 | pr-d | 48 | 0.282259 |
| 77 | pr-d | 19 | 0.127786 |
| 78 | pr-d | 76 | 0.403297 |
| 79 | pr-d | 70 | 0.379731 |
| 2 | pr-mb | 49 | 0.102762 |
| 3 | pr-mb | 108 | 0.24265 |
| 4 | pr-mb | 141 | 0.330292 |
| 5 | pr-mb | 152 | 0.353303 |
| 6 | pr-mb | 155 | 0.357914 |
| 7 | pr-mb | 210 | 0.500951 |
| 8 | pr-mb | 194 | 0.447743 |
| 9 | pr-mb | 201 | 0.467631 |
| 10 | pr-mb | 129 | 0.284131 |
| 11 | pr-mb | 169 | 0.390225 |
| 12 | pr-mb | 204 | 0.49757 |
| 13 | pr-mb | 208 | 0.509679 |
| 14 | pr-mb | 216 | 0.523641 |
| 15 | pr-mb | 216 | 0.523641 |
| 16 | pr-mb | 195 | 0.457563 |
| 17 | pr-mb | 272 | 0.628744 |
| 18 | pr-mb | 356 | 0.802645 |
| 19 | pr-mb | 368 | 0.826769 |
| 20 | pr-mb | 372 | 0.832803 |
| 21 | pr-mb | 247 | 0.480858 |
| 22 | pr-mb | 342 | 0.699955 |
| 23 | pr-mb | 276 | 0.525689 |
| 24 | pr-mb | 279 | 0.530202 |
| 25 | pr-mb | 215 | 0.409246 |
| 26 | pr-mb | 298 | 0.590885 |
| 27 | pr-mb | 175 | 0.341271 |
| 28 | pr-mb | 235 | 0.462267 |
| 29 | pr-mb | 200 | 0.411815 |
| 30 | pr-mb | 183 | 0.399428 |
| 31 | pr-mb | 194 | 0.426957 |
| 32 | pr-mb | 203 | 0.444834 |
| 33 | pr-mb | 127 | 0.277683 |
| 34 | pr-mb | 183 | 0.429036 |
| 35 | pr-mb | 185 | 0.429729 |
| 36 | pr-mb | 204 | 0.487775 |
| 37 | pr-mb | 211 | 0.564718 |
| 38 | pr-mb | 231 | 0.620167 |
| 39 | pr-mb | 231 | 0.620167 |
| 40 | pr-mb | 135 | 0.349129 |
| 41 | pr-mb | 205 | 0.590059 |
| 42 | pr-mb | 288 | 0.847562 |
| 43 | pr-mb | 292 | 0.855025 |
| 44 | pr-mb | 165 | 0.534572 |
| 45 | pr-mb | 257 | 0.828004 |
| 46 | pr-mb | 310 | 0.950643 |
| 47 | pr-mb | 317 | 0.973367 |
| 48 | pr-mb | 384 | 1.15774 |
| 49 | pr-mb | 239 | 0.826285 |
| 50 | pr-mb | 356 | 1.1765 |
| 51 | pr-mb | 496 | 1.53497 |
| 52 | pr-mb | 269 | 0.987216 |
| 53 | pr-mb | 473 | 1.55443 |
| 54 | pr-mb | 672 | 2.05282 |
| 55 | pr-mb | 357 | 1.29309 |
| 56 | pr-mb | 460 | 1.55844 |
| 57 | pr-mb | 717 | 2.2729 |
| 58 | pr-mb | 763 | 2.39261 |
| 59 | pr-mb | 772 | 2.41064 |
| 60 | pr-mb | 775 | 2.41771 |
| 61 | pr-mb | 694 | 2.26021 |
| 62 | pr-mb | 694 | 2.26021 |
| 63 | pr-mb | 704 | 2.34801 |
| 64 | pr-mb | 528 | 1.90622 |
| 65 | pr-mb | 529 | 1.90797 |
| 66 | pr-mb | 468 | 1.74727 |
| 67 | pr-mb | 465 | 1.72677 |
| 68 | pr-mb | 284 | 1.11223 |
| 69 | pr-mb | 281 | 1.10344 |
| 70 | pr-mb | 246 | 0.952198 |
| 71 | pr-mb | 243 | 0.929134 |
| 72 | pr-mb | 101 | 0.397897 |
| 73 | pr-mb | 101 | 0.397897 |
| 74 | pr-mb | 86 | 0.338721 |
| 94 | pr-ml | 17 | 0.069038 |
| 95 | pr-ml | 104 | 0.359429 |
| 96 | pr-ml | 200 | 0.650576 |
| 97 | pr-ml | 278 | 0.843889 |
| 98 | pr-ml | 371 | 1.10336 |
| 99 | pr-ml | 389 | 1.15048 |
| 100 | pr-ml | 359 | 1.06878 |
| 101 | pr-ml | 199 | 0.606346 |
| 102 | pr-ml | 266 | 0.793425 |
| 103 | pr-ml | 300 | 0.900445 |
| 104 | pr-ml | 286 | 0.823486 |
| 105 | pr-ml | 272 | 0.772831 |
| 106 | pr-ml | 269 | 0.757054 |
| 107 | pr-ml | 273 | 0.76325 |
| 108 | pr-ml | 164 | 0.453897 |
| 109 | pr-ml | 212 | 0.553189 |
| 110 | pr-ml | 257 | 0.656555 |
| 111 | pr-ml | 268 | 0.679933 |
| 112 | pr-ml | 135 | 0.355499 |
| 113 | pr-ml | 181 | 0.431546 |
| 114 | pr-ml | 210 | 0.499231 |
| 115 | pr-ml | 211 | 0.499928 |
| 116 | pr-ml | 183 | 0.426082 |
| 117 | pr-ml | 183 | 0.426082 |
| 118 | pr-ml | 184 | 0.427401 |
| 119 | pr-ml | 139 | 0.308429 |
| 120 | pr-ml | 139 | 0.308429 |
| 121 | pr-ml | 135 | 0.300131 |
| 122 | pr-ml | 92 | 0.19068 |
| 123 | pr-ml | 92 | 0.186994 |
| 124 | pr-ml | 100 | 0.207004 |
| 125 | pr-ml | 71 | 0.132463 |

**Table 3.** Area of OFA contact points on the lower m2 per time step. Area is given in mm^2^.

| Timestep | Facet | Triangles | Area |
| --- | --- | --- | --- |
| 16 | hy-mb | 77 | 0.174271 |
| 17 | hy-mb | 149 | 0.348433 |
| 18 | hy-mb | 264 | 0.595501 |
| 19 | hy-mb | 308 | 0.691528 |
| 20 | hy-mb | 317 | 0.709146 |
| 21 | hy-mb | 245 | 0.549375 |
| 22 | hy-mb | 389 | 0.849617 |
| 23 | hy-mb | 243 | 0.512314 |
| 24 | hy-mb | 263 | 0.559634 |
| 25 | hy-mb | 296 | 0.626219 |
| 26 | hy-mb | 394 | 0.835021 |
| 27 | hy-mb | 246 | 0.522042 |
| 28 | hy-mb | 317 | 0.673531 |
| 29 | hy-mb | 287 | 0.615482 |
| 30 | hy-mb | 233 | 0.498392 |
| 31 | hy-mb | 254 | 0.53869 |
| 32 | hy-mb | 269 | 0.569065 |
| 33 | hy-mb | 36 | 0.07537 |
| 34 | hy-mb | 163 | 0.357155 |
| 35 | hy-mb | 197 | 0.456426 |
| 36 | hy-mb | 218 | 0.505681 |
| 37 | hy-mb | 199 | 0.471463 |
| 38 | hy-mb | 214 | 0.525138 |
| 39 | hy-mb | 214 | 0.525138 |
| 41 | hy-mb | 44 | 0.105987 |
| 42 | hy-mb | 125 | 0.308277 |
| 43 | hy-mb | 132 | 0.322671 |
| 45 | hy-mb | 35 | 0.089961 |
| 46 | hy-mb | 65 | 0.171665 |
| 47 | hy-mb | 61 | 0.166448 |
| 48 | hy-mb | 92 | 0.243545 |
| 50 | hy-mb | 10 | 0.02825 |
| 51 | hy-mb | 48 | 0.125189 |
| 28 | me-db | 31 | 0.063488 |
| 29 | me-db | 89 | 0.171924 |
| 30 | me-db | 134 | 0.254732 |
| 31 | me-db | 148 | 0.279573 |
| 32 | me-db | 158 | 0.295429 |
| 33 | me-db | 148 | 0.275973 |
| 34 | me-db | 202 | 0.392363 |
| 35 | me-db | 79 | 0.142096 |
| 36 | me-db | 100 | 0.181785 |
| 37 | me-db | 138 | 0.263189 |
| 38 | me-db | 149 | 0.288661 |
| 39 | me-db | 149 | 0.288661 |
| 40 | me-db | 148 | 0.279468 |
| 41 | me-db | 195 | 0.405683 |
| 42 | me-db | 233 | 0.524564 |
| 43 | me-db | 238 | 0.558718 |
| 44 | me-db | 236 | 0.536386 |
| 45 | me-db | 270 | 0.658645 |
| 46 | me-db | 305 | 0.772985 |
| 47 | me-db | 113 | 0.240007 |
| 48 | me-db | 138 | 0.323178 |
| 49 | me-db | 158 | 0.377789 |
| 50 | me-db | 196 | 0.534528 |
| 51 | me-db | 232 | 0.652424 |
| 52 | me-db | 244 | 0.715351 |
| 53 | me-db | 308 | 0.90413 |
| 54 | me-db | 461 | 1.27823 |
| 55 | me-db | 529 | 1.44066 |
| 56 | me-db | 586 | 1.57942 |
| 57 | me-db | 502 | 1.44148 |
| 58 | me-db | 525 | 1.48021 |
| 59 | me-db | 529 | 1.48918 |
| 60 | me-db | 526 | 1.48099 |
| 61 | me-db | 300 | 0.899105 |
| 62 | me-db | 296 | 0.891198 |
| 63 | me-db | 200 | 0.598746 |
| 64 | me-db | 108 | 0.228558 |
| 65 | me-db | 107 | 0.227759 |
| 66 | me-db | 74 | 0.167159 |
| 67 | me-db | 74 | 0.167159 |
| 68 | me-db | 19 | 0.037955 |
| 69 | me-db | 17 | 0.035843 |
| 48 | en-db | 28 | 0.050461 |
| 50 | en-db | 83 | 0.145763 |
| 51 | en-db | 207 | 0.404714 |
| 52 | en-db | 84 | 0.157326 |
| 53 | en-db | 226 | 0.470383 |
| 54 | en-db | 433 | 0.959081 |
| 55 | en-db | 199 | 0.424351 |
| 56 | en-db | 252 | 0.557517 |
| 57 | en-db | 496 | 1.20438 |
| 58 | en-db | 549 | 1.32506 |
| 59 | en-db | 563 | 1.36005 |
| 60 | en-db | 563 | 1.36005 |
| 61 | en-db | 501 | 1.23643 |
| 62 | en-db | 499 | 1.23016 |
| 63 | en-db | 554 | 1.35239 |
| 64 | en-db | 481 | 1.18059 |
| 65 | en-db | 497 | 1.21403 |
| 66 | en-db | 586 | 1.42195 |
| 67 | en-db | 589 | 1.43694 |
| 68 | en-db | 560 | 1.34905 |
| 69 | en-db | 560 | 1.34905 |
| 70 | en-db | 626 | 1.53334 |
| 71 | en-db | 626 | 1.53444 |
| 72 | en-db | 553 | 1.36579 |
| 73 | en-db | 556 | 1.3764 |
| 74 | en-db | 571 | 1.42611 |
| 75 | en-db | 90 | 0.174922 |
| 76 | en-db | 186 | 0.403572 |
| 77 | en-db | 222 | 0.482973 |
| 78 | en-db | 32 | 0.070168 |
| 79 | en-db | 32 | 0.070168 |
| 80 | en-db | 24 | 0.052609 |
| 81 | en-db | 24 | 0.052609 |
| 82 | en-db | 30 | 0.066834 |
| 48 | hld-m | 98 | 0.157364 |
| 50 | hld-m | 128 | 0.216614 |
| 51 | hld-m | 231 | 0.404148 |
| 52 | hld-m | 93 | 0.151416 |
| 53 | hld-m | 238 | 0.435313 |
| 54 | hld-m | 381 | 0.723979 |
| 55 | hld-m | 19 | 0.034513 |
| 55 | hld-m | 176 | 0.322683 |
| 56 | hld-m | 273 | 0.500709 |
| 57 | hld-m | 465 | 0.895914 |
| 58 | hld-m | 488 | 0.942578 |
| 59 | hld-m | 488 | 0.942578 |
| 60 | hld-m | 490 | 0.946395 |
| 61 | hld-m | 517 | 1.00636 |
| 62 | hld-m | 517 | 1.00636 |
| 63 | hld-m | 572 | 1.13992 |
| 64 | hld-m | 573 | 1.15873 |
| 65 | hld-m | 588 | 1.20217 |
| 66 | hld-m | 661 | 1.37444 |
| 67 | hld-m | 666 | 1.38687 |
| 68 | hld-m | 679 | 1.45157 |
| 69 | hld-m | 681 | 1.45537 |
| 70 | hld-m | 803 | 1.75522 |
| 71 | hld-m | 806 | 1.7642 |
| 72 | hld-m | 881 | 1.94793 |
| 73 | hld-m | 881 | 1.94793 |
| 74 | hld-m | 908 | 2.02062 |
| 75 | hld-m | 517 | 1.11276 |
| 76 | hld-m | 773 | 1.69395 |
| 77 | hld-m | 971 | 2.2743 |
| 78 | hld-m | 96 | 0.203778 |
| 78 | hld-m | 32 | 0.093363 |
| 78 | hld-m | 96 | 0.196024 |
| 79 | hld-m | 97 | 0.205521 |
| 79 | hld-m | 43 | 0.120378 |
| 79 | hld-m | 101 | 0.205326 |
| 80 | hld-m | 800 | 1.89872 |
| 81 | hld-m | 807 | 1.91581 |
| 82 | hld-m | 890 | 2.16209 |
| 83 | hld-m | 145 | 0.319354 |
| 84 | hld-m | 379 | 0.857775 |
| 85 | hld-m | 374 | 0.905176 |
| 86 | hld-m | 478 | 1.14515 |
| 87 | hld-m | 404 | 0.940854 |
| 88 | hld-m | 227 | 0.491471 |
| 89 | hld-m | 313 | 0.684436 |
| 90 | hld-m | 174 | 0.379562 |
| 91 | hld-m | 348 | 0.782308 |
| 92 | hld-m | 154 | 0.324864 |
| 93 | hld-m | 36 | 0.071391 |
| 51 | pr-db | 68 | 0.279327 |
| 52 | pr-db | 32 | 0.139895 |
| 53 | pr-db | 140 | 0.51048 |
| 54 | pr-db | 232 | 0.77795 |
| 55 | pr-db | 171 | 0.577647 |
| 56 | pr-db | 193 | 0.673546 |
| 57 | pr-db | 229 | 0.799769 |
| 58 | pr-db | 265 | 0.891848 |
| 59 | pr-db | 282 | 0.936232 |
| 60 | pr-db | 279 | 0.93159 |
| 61 | pr-db | 190 | 0.617606 |
| 62 | pr-db | 190 | 0.617606 |
| 63 | pr-db | 139 | 0.462354 |
| 64 | pr-db | 69 | 0.194382 |
| 65 | pr-db | 61 | 0.159675 |
| 66 | pr-db | 61 | 0.155266 |
| 67 | pr-db | 61 | 0.155266 |
| 68 | pr-db | 74 | 0.187259 |
| 69 | pr-db | 72 | 0.183774 |
| 70 | pr-db | 84 | 0.218844 |
| 71 | pr-db | 82 | 0.213237 |
| 72 | pr-db | 87 | 0.237545 |
| 73 | pr-db | 87 | 0.237545 |
| 74 | pr-db | 89 | 0.24291 |
| 68 | pr-ml | 115 | 0.266389 |
| 69 | pr-ml | 119 | 0.277406 |
| 70 | pr-ml | 259 | 0.698908 |
| 71 | pr-ml | 287 | 0.778499 |
| 75 | pr-ml | 277 | 0.757742 |
| 83 | pr-ml | 629 | 1.68086 |
| 84 | pr-ml | 741 | 1.98051 |
| 85 | pr-ml | 601 | 1.57227 |
| 86 | pr-ml | 669 | 1.74263 |
| 87 | pr-ml | 716 | 1.85696 |
| 88 | pr-ml | 592 | 1.52209 |
| 89 | pr-ml | 659 | 1.70516 |
| 90 | pr-ml | 593 | 1.51371 |
| 91 | pr-ml | 665 | 1.68944 |
| 92 | pr-ml | 532 | 1.32615 |
| 93 | pr-ml | 425 | 1.05152 |
| 94 | pr-ml | 326 | 0.800969 |
| 95 | pr-ml | 232 | 0.53227 |
| 96 | pr-ml | 109 | 0.249816 |
| 97 | pr-ml | 12 | 0.035084 |
| 68 | pr-d | 77 | 0.15032 |
| 69 | pr-d | 78 | 0.152062 |
| 70 | pr-d | 179 | 0.431696 |
| 71 | pr-d | 184 | 0.441443 |
| 72 | pr-d | 718 | 1.83046 |
| 73 | pr-d | 718 | 1.83046 |
| 74 | pr-d | 754 | 1.91487 |
| 75 | pr-d | 263 | 0.630946 |
| 76 | pr-d | 928 | 2.45185 |
| 77 | pr-d | 1134 | 3.02281 |
| 78 | pr-d | 931 | 2.48264 |
| 79 | pr-d | 952 | 2.53976 |
| 80 | pr-d | 1147 | 2.99779 |
| 81 | pr-d | 1162 | 3.024591 |
| 82 | pr-d | 1253 | 3.20016 |
| 83 | pr-d | 318 | 0.750267 |
| 84 | pr-d | 405 | 0.927985 |
| 85 | pr-d | 194 | 0.448611 |
| 86 | pr-d | 214 | 0.487593 |
| 87 | pr-d | 389 | 0.875451 |
| 88 | pr-d | 430 | 0.99892 |
| 89 | pr-d | 555 | 1.30957 |
| 90 | pr-d | 299 | 0.621724 |
| 91 | pr-d | 413 | 0.913104 |
| 92 | pr-d | 331 | 0.703399 |
| 93 | pr-d | 210 | 0.443639 |
| 94 | pr-d | 161 | 0.347581 |
| 95 | pr-d | 148 | 0.319752 |
| 96 | pr-d | 135 | 0.275483 |
| 97 | pr-d | 125 | 0.243522 |
| 98 | pr-d | 103 | 0.182474 |
| 99 | pr-d | 98 | 0.172058 |
| 82 | hy-db | 41 | 0.077167 |
| 87 | hy-db | 55 | 0.101116 |
| 88 | hy-db | 68 | 0.128764 |
| 89 | hy-db | 199 | 0.409625 |
| 91 | hy-db | 92 | 0.175634 |
| 92 | hy-db | 19 | 0.031984 |
| 96 | hy-l | 487 | 1.36385 |
| 97 | hy-l | 736 | 2.04463 |
| 98 | hy-l | 883 | 2.39791 |
| 99 | hy-l | 903 | 2.44154 |
| 100 | hy-l | 796 | 2.16573 |
| 101 | hy-l | 356 | 0.981165 |
| 102 | hy-l | 536 | 1.44127 |
| 103 | hy-l | 644 | 1.73116 |
| 104 | hy-l | 588 | 1.55448 |
| 105 | hy-l | 527 | 1.38499 |
| 106 | hy-l | 545 | 1.42481 |
| 107 | hy-l | 555 | 1.45909 |
| 108 | hy-l | 249 | 0.673021 |
| 109 | hy-l | 397 | 1.05043 |
| 110 | hy-l | 507 | 1.29515 |
| 111 | hy-l | 524 | 1.3437 |
| 112 | hy-l | 262 | 0.723822 |
| 113 | hy-l | 372 | 0.962441 |
| 114 | hy-l | 470 | 1.2109 |
| 115 | hy-l | 477 | 1.22889 |
| 116 | hy-l | 448 | 1.14011 |
| 117 | hy-l | 449 | 1.14288 |
| 118 | hy-l | 460 | 1.15723 |
| 119 | hy-l | 422 | 1.0486 |
| 120 | hy-l | 423 | 1.04974 |
| 121 | hy-l | 439 | 1.08643 |
| 122 | hy-l | 404 | 0.975743 |
| 123 | hy-l | 415 | 0.995426 |
| 124 | hy-l | 442 | 1.04489 |
| 125 | hy-l | 432 | 1.01775 |
| 126 | hy-l | 278 | 0.641443 |
| 127 | hy-l | 320 | 0.725232 |
| 128 | hy-l | 338 | 0.760606 |
| 129 | hy-l | 342 | 0.758405 |
| 130 | hy-l | 341 | 0.753423 |
| 131 | hy-l | 191 | 0.419527 |
| 132 | hy-l | 192 | 0.411269 |
| 133 | hy-l | 184 | 0.380545 |
| 134 | hy-l | 148 | 0.298918 |
| 135 | hy-l | 123 | 0.249947 |
| 136 | hy-l | 75 | 0.1514 |
| 137 | hy-l | 22 | 0.045916 |


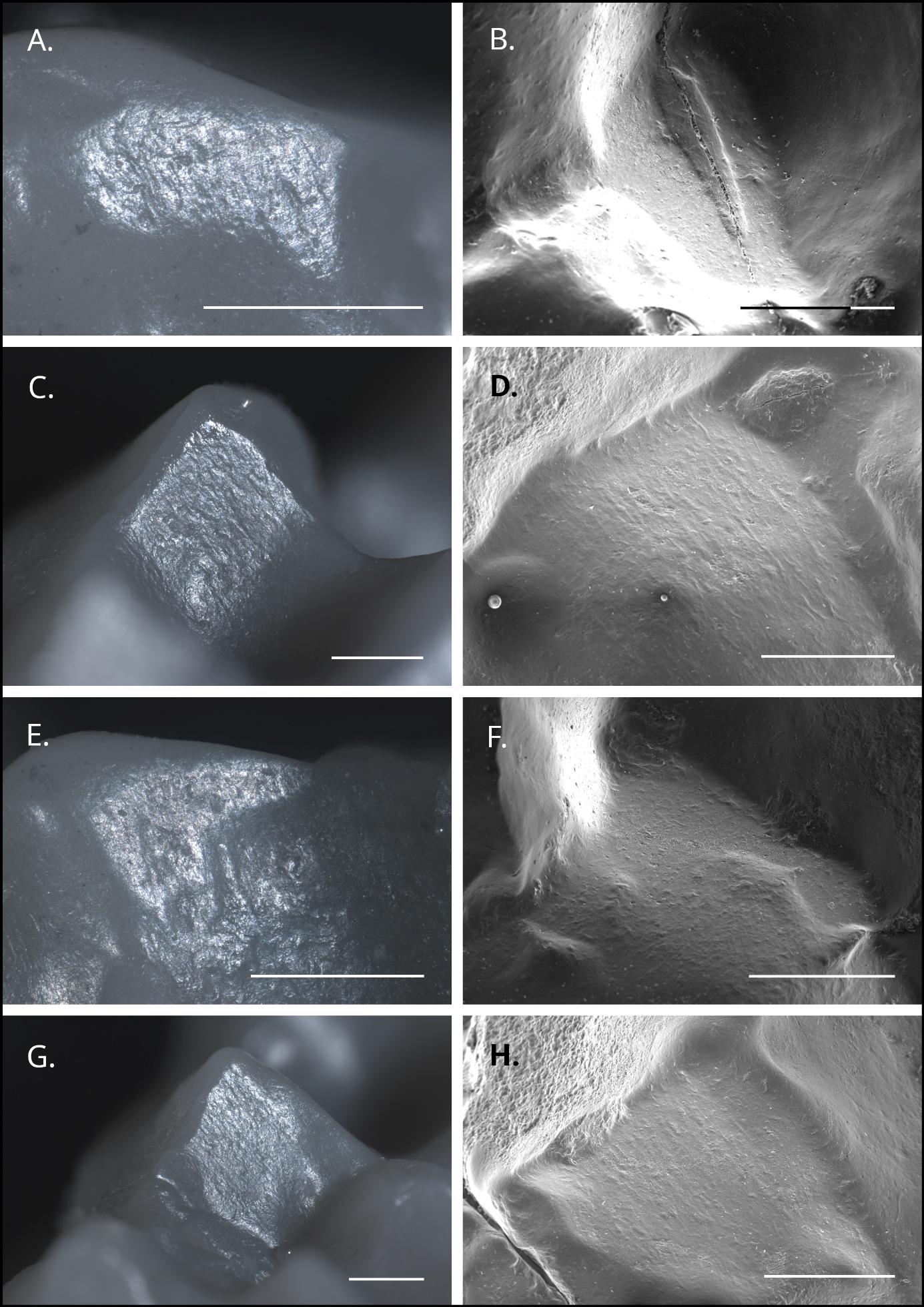


**Figure 1.** Detailed pictures of (A, B) facet M1-PA-ml, (C, D) facet M1-PA-dl, (E, F) facet M1-ME-ml and (G, H) facet M1-ME-dl using (A, C, E, G) a digital microscope and (B, D, F, H) a scanning electron microscope. The specimens of which the pictures were taken are (A, E) ZFMK-MAM-2016.0936 dex, (B, D) ZFMK-MAM-2016.0986 dex and (C, D, G, H) ZFMK-MAM-2016.0932 dex. The scale bars represent 1 mm.


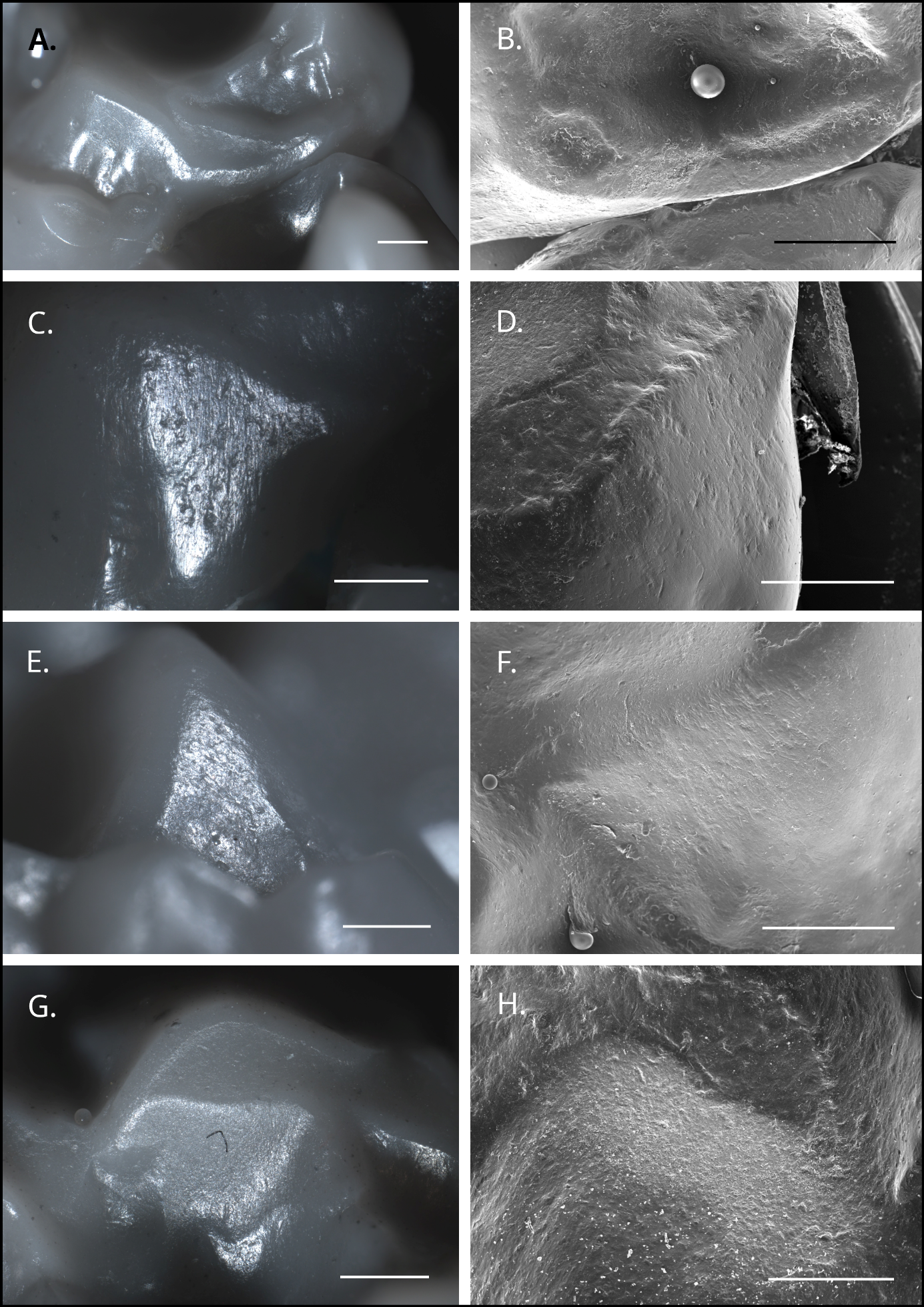


**Figure 2.** Detailed pictures of (A, B) facet M1-PRECNG-m, (C, D) facet M1-PR-ml, (E, F) facet M1-PR-d and (G, H) facet M1-PR-b using (A, C, E, G) a digital microscope and (B, D, F, H) a scanning electron microscope. The specimens of which the pictures were taken are (A) ZFMK-MAM-2016.0945 dex, (B, E, F) ZFMK-MAM-2016.0932 dex, (C, D, H) ZFMK-MAM-2016.0939 dex and (G) ZFMK-MAM-2017.0575 sin. The scale bars represent 1 mm.


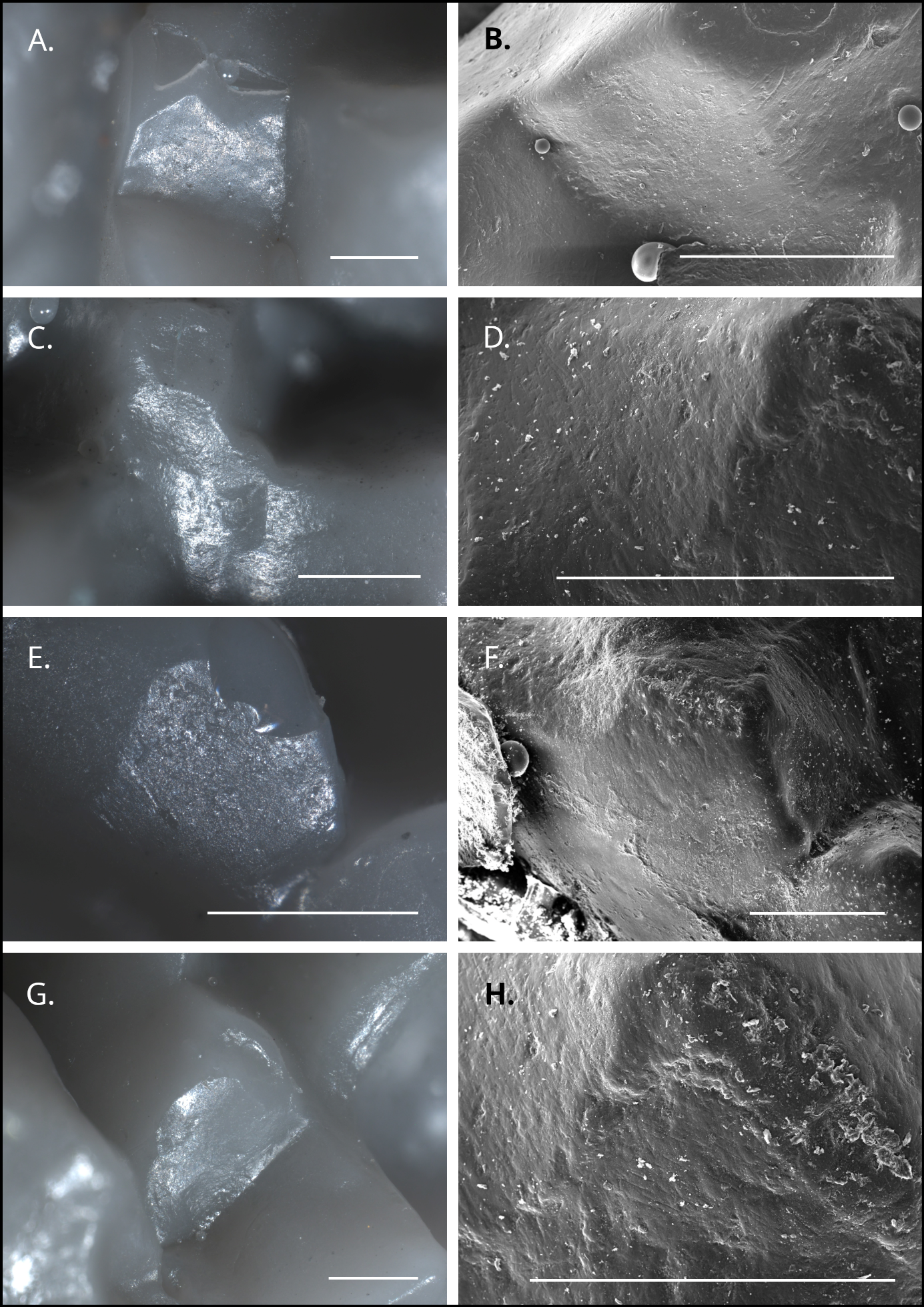


**Figure 3.** Detailed pictures of (A, B) facet M1-MTCL-ml, (C, D) facet M1-MTCL-mb, (E, F) facet M1-MTCL-d and (G, H) facet M1-MTCL-b using (A, C, E, G) a digital microscope and (B, D, F, H) a scanning electron microscope. The specimens of which the pictures were taken are (A) ZFMK-MAM-2016.0969 dex, (B) ZFMK-MAM-2016.0932 dex, (C) ZFMK-MAM-2016.0968 dex, (D, F, H) ZFMK-MAM-2016.0939 dex, (E) ZFMK-MAM-2016.0897 dex and (G) ZFMK-MAM-2016.0932 sin. The scale bars represent 1 mm.


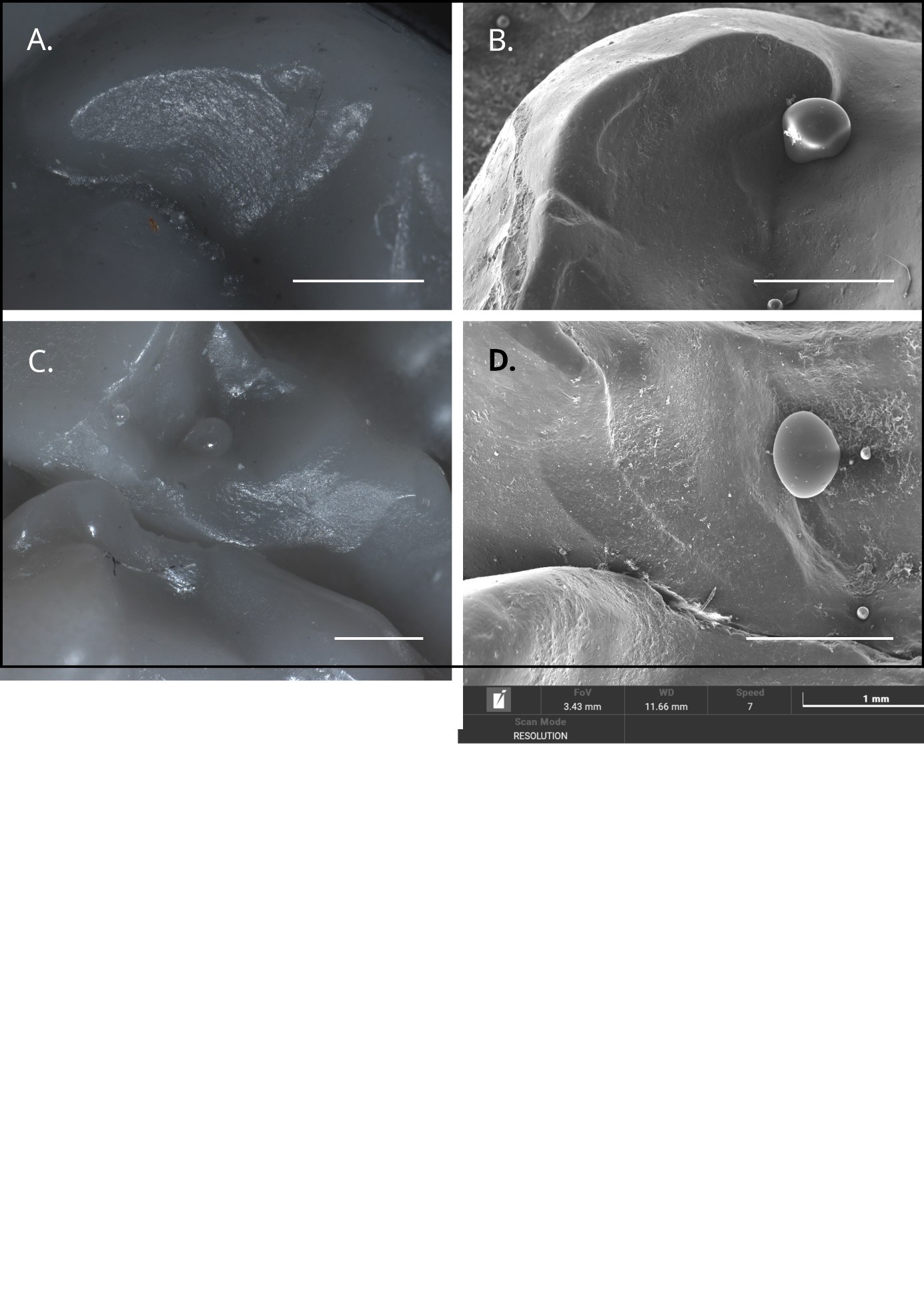


**Figure 4.** Detailed pictures of (A, B) facet M1-HY-mb and (C, D) facet M1-POCNG-d using (A, C) a digital microscope and (B, D) a scanning electron microscope. The specimens of which the pictures were taken are (A) ZFMK-MAM-2016.0936 dex, (B) ZFMK-MAM-2016.0936 sin, (C) ZFMK-MAM-2016.0897 dex and (D) ZFMK-MAM-2017.0578 sin. The scale bars represent 1 mm.


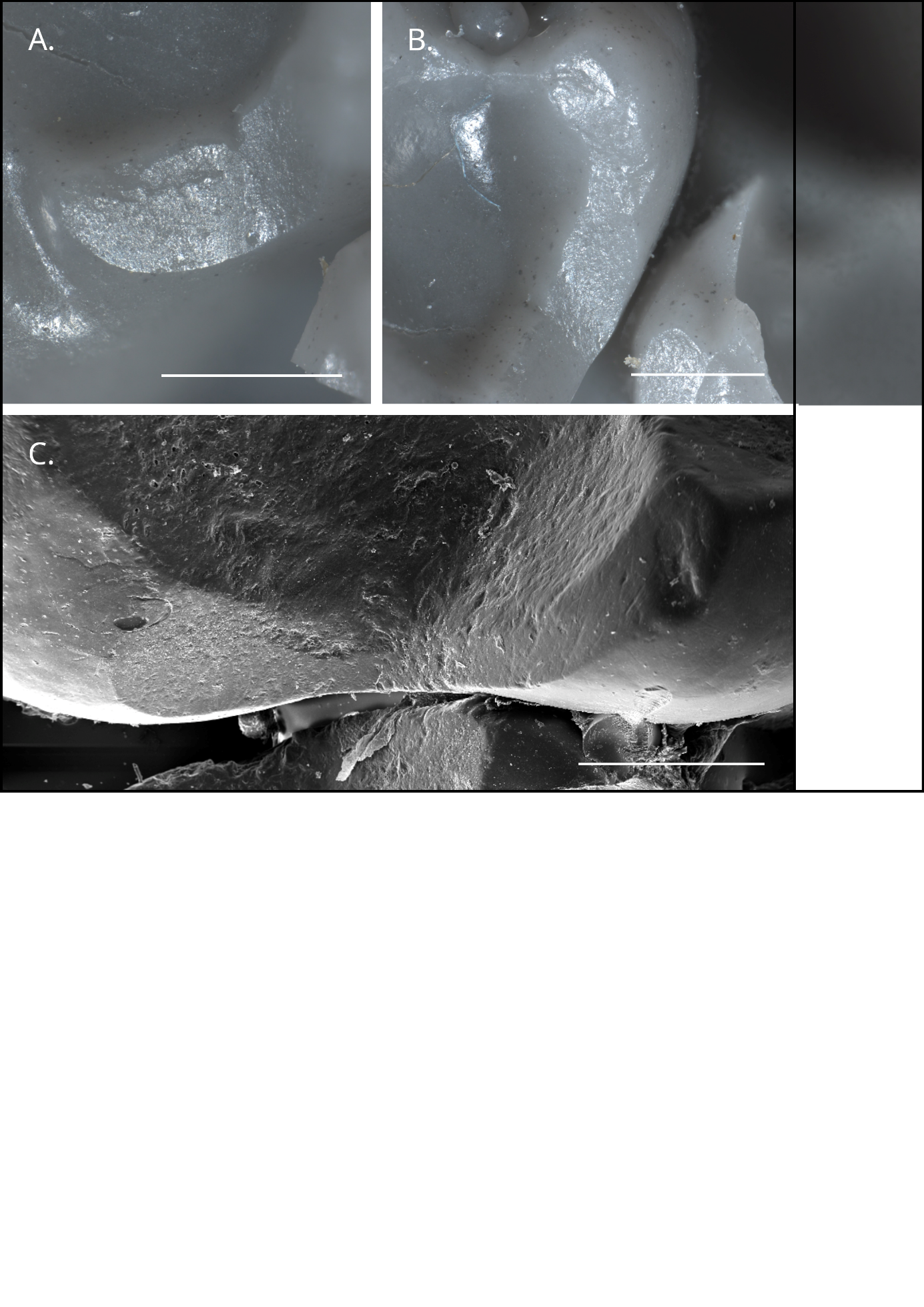


**Figure 5.** Detailed pictures of (A) facet P4-PRECNG-mb, (B) facet P4-PRECNG-ml and (C) an overview of the two facets together using (A, B) a digital microscope and (C) a scanning electron microscope. The specimens of which the pictures were taken are (A, B) ZFMK-MAM-2016.0968 dex and (C) ZFMK-MAM-2016.0939 dex. The scale bars represent 1 mm.


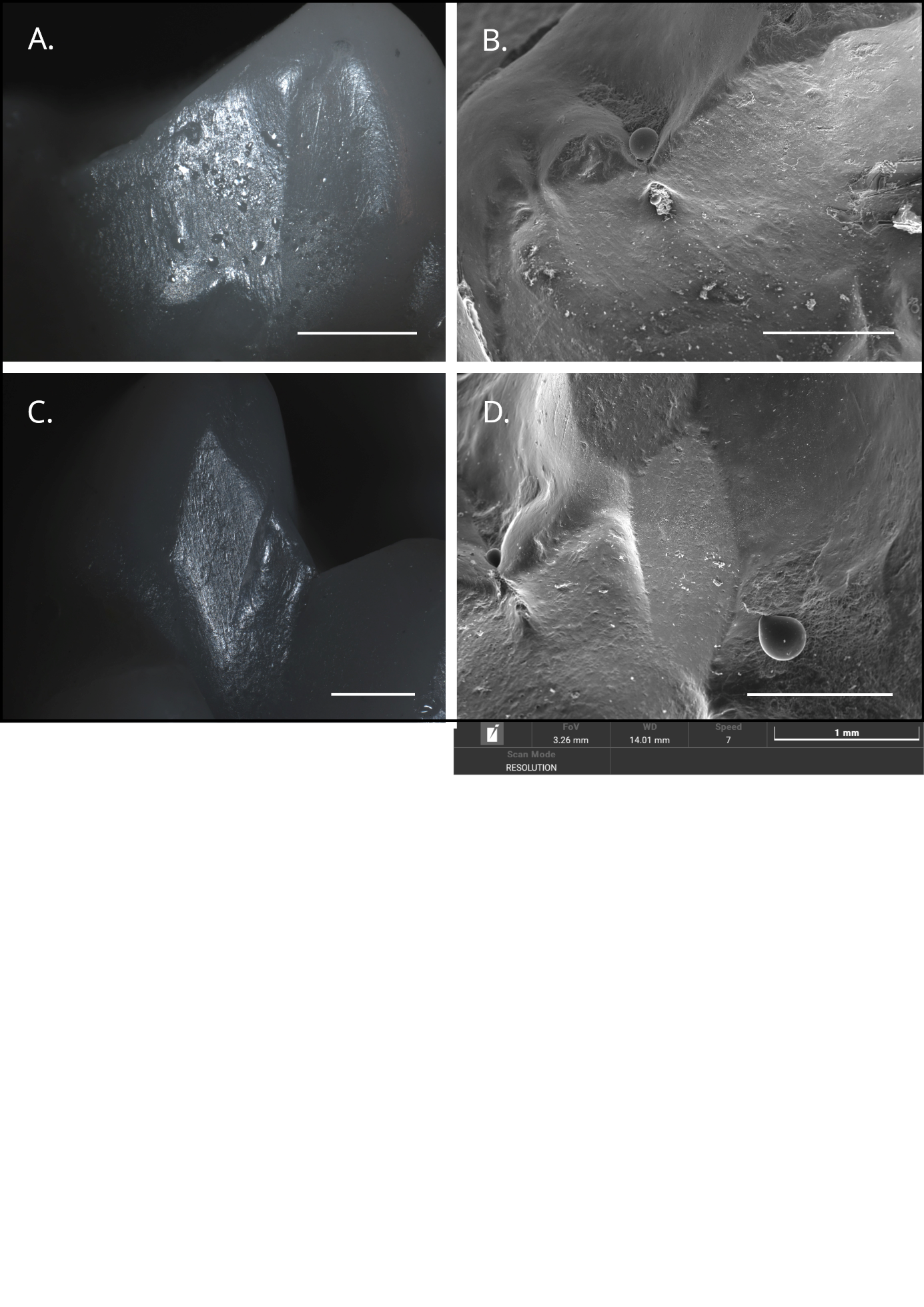


**Figure 6.** Detailed pictures of (A, B) facet P4-PA-dl, (C, D) facet P4-PA-ml, using (A, C) a digital microscope and (B, D) a scanning electron microscope. The specimens of which the pictures were taken are (A) ZFMK-MAM-2016.0897 dex, (B) ZFMK-MAM-2016.0936 sin, (C) ZFMK-MAM-2016.0939 sin and (D) ZFMK-MAM-2016.0939 dex. The scale bars represent 1 mm.


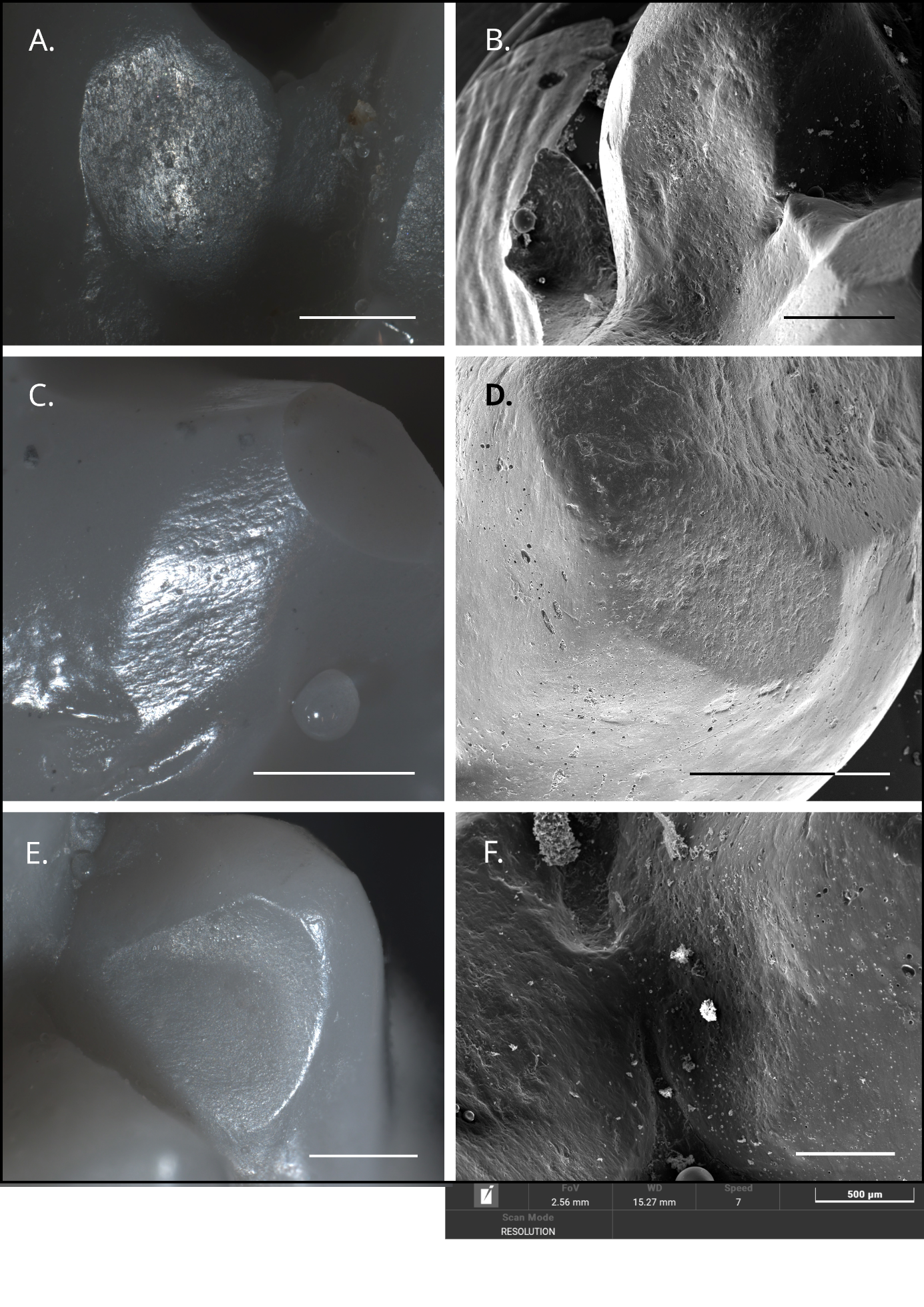


**Figure 7.** Detailed pictures of (A, B) facet P4-PR-mb, (C, D) facet P4-PR-ml and (E, F) facet P4-PR-d using (A, C, E) a digital microscope and (B, D, F) a scanning electron microscope. The specimens of which the pictures were taken are (A, E) ZFMK-MAM-2017.0578 dex, (B, F) ZFMK-MAM-2016.0939 dex, (C) ZFMK-MAM-2016.0897 dex and (D) ZFMK-MAM-2016.0939 dex. The scale bars represent 1 mm.


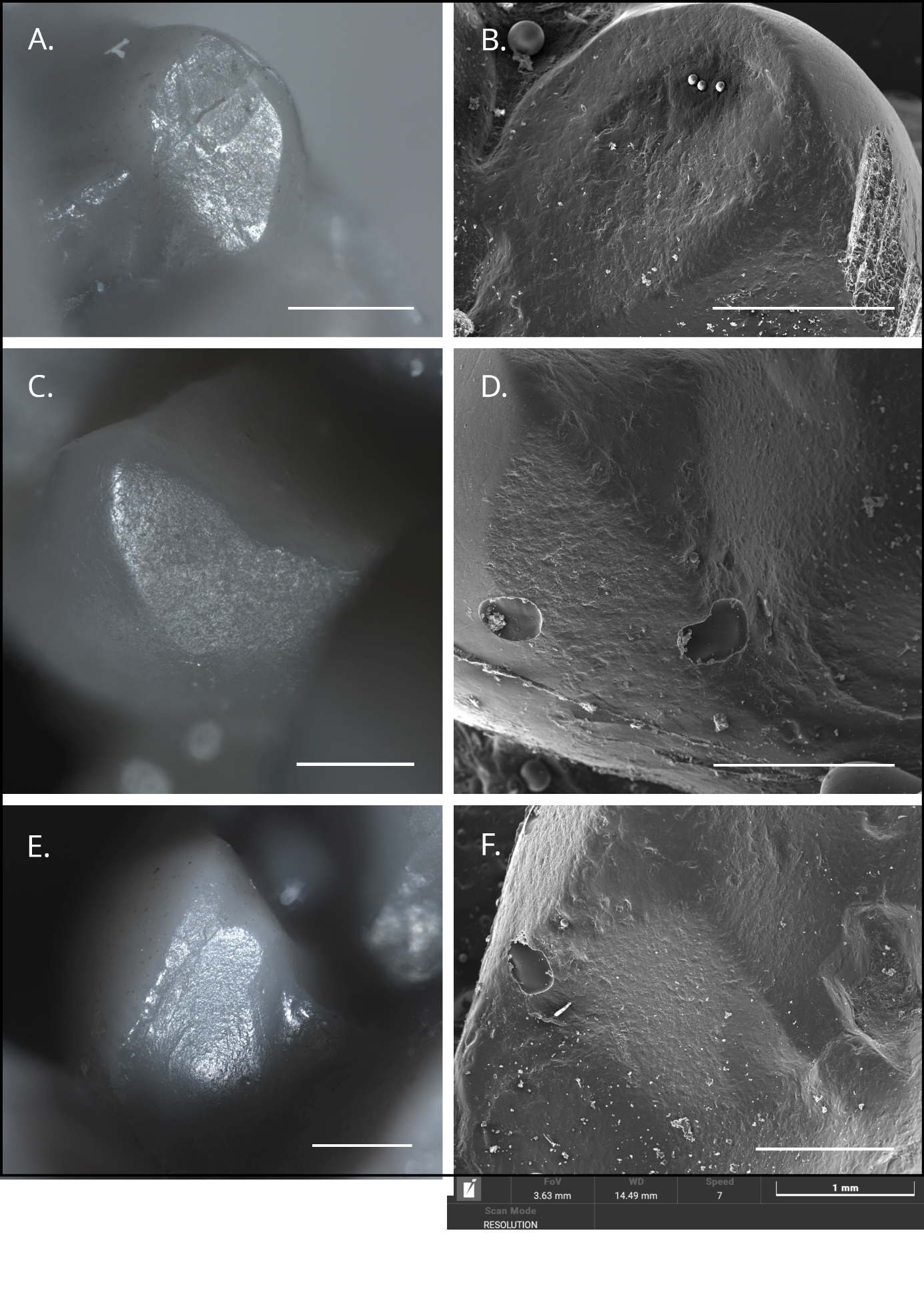


**Figure 8.** Detailed pictures of (A, B) facet P4-HY-mb, (C, D) facet P4-HY-dl and (E, F) facet P4-HY-db using (A, C, E) a digital microscope and (B, D, F) a scanning electron microscope. The specimens of which the pictures were taken are (A) ZFMK-MAM-2016.0968 dex, (B) ZFMK-MAM-2016.0939 dex, (C) ZFMK-MAM-2017.0578 dex, (D, F) ZFMK-MAM-2016.0907 sin and (E) ZFMK-MAM-2016.0936 sin. The scale bars represent 1 mm.


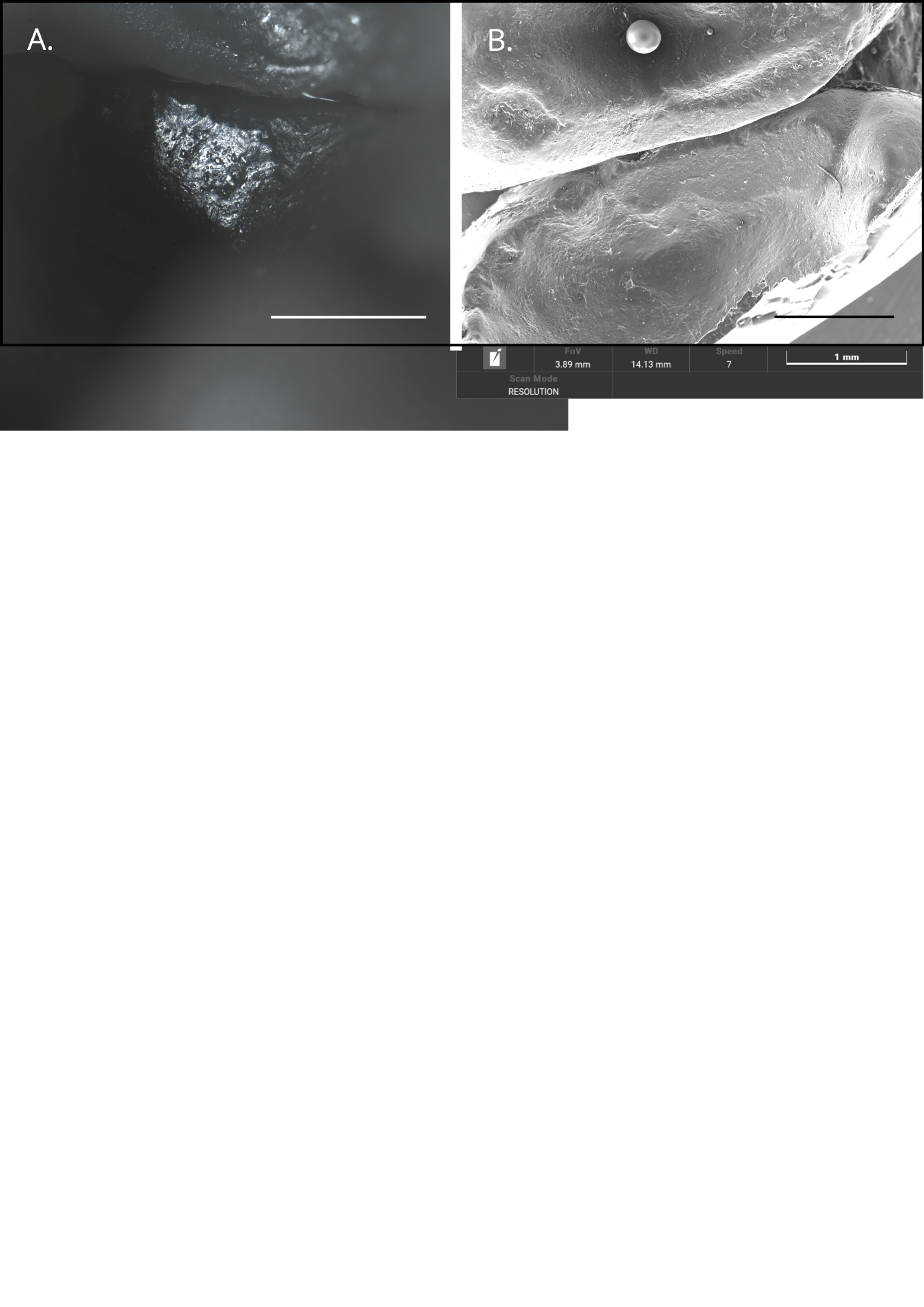


**Figure 9.** Detailed pictures of facet P4-POCNG-m using (A) a digital microscope and (B) a scanning electron microscope. The specimens of which the pictures were taken are (A) ZFMK-MAM-2016.0897 dex and (B) ZFMK-MAM-2016.0932 dex. The scale bars represent 1 mm.


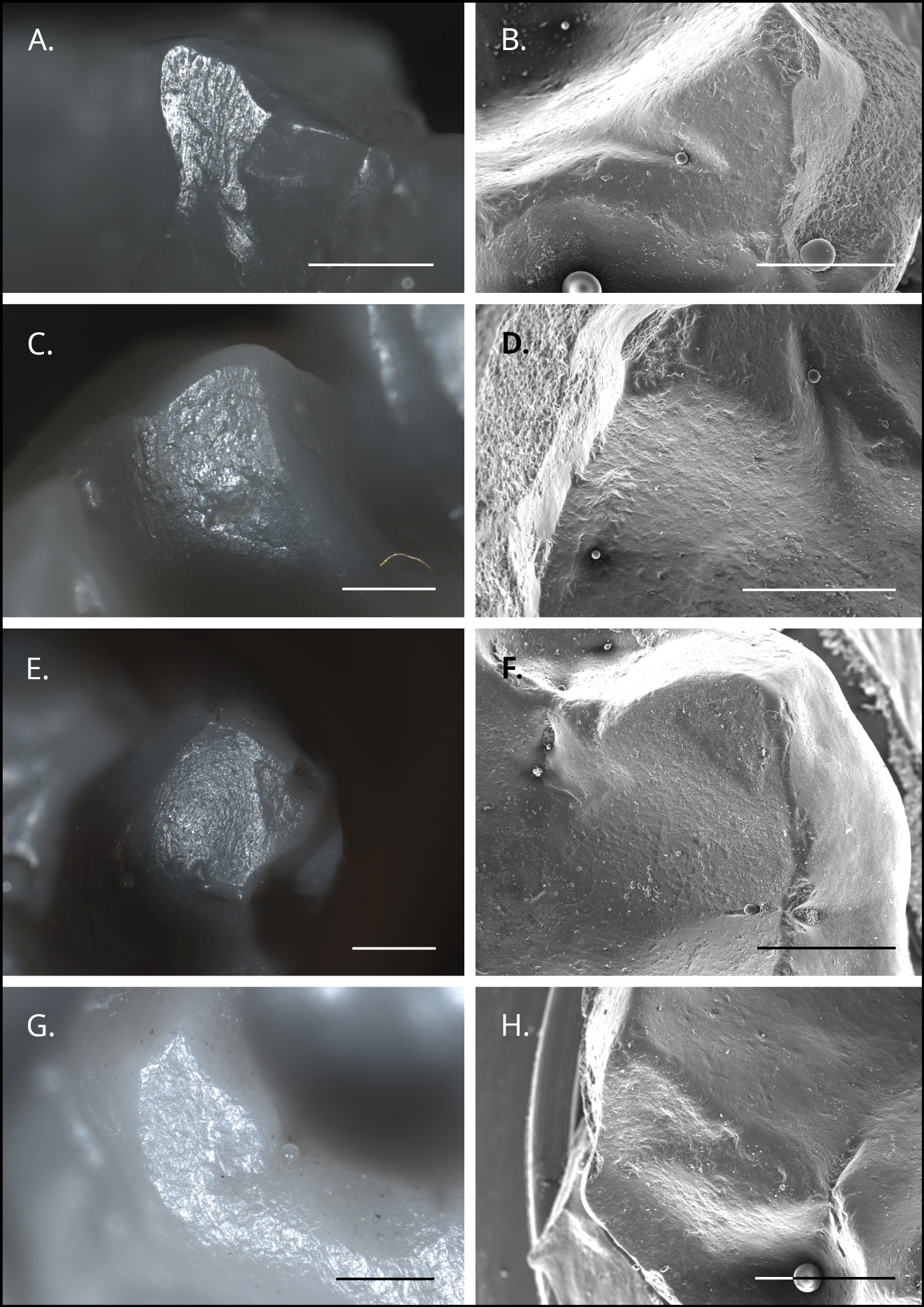


**Figure 10.** Detailed pictures of (A, B) facet M2-PA-ml, (C, D) facet M2-PA-dl, (E, F) facet M2-ME-ml and (G, H) facet M2-ME-dl using (A, C, E, G) a digital microscope and (B, D, F, H) a scanning electron microscope. The specimens of which the pictures were taken are (A) ZFMK-MAM-2016.0936 dex, (B, D) ZFMK-MAM-2016.0932 dex, (C) ZFMK-MAM-2016.0945 dex, (E) ZFMK-MAM-2016.0968 sin, (F) ZFMK-MAM-2016.0986 dex, (G) ZFMK-MAM-2016.0939 dex and (H) ZFMK-MAM-2016.0932 sin. The scale bars represent 1 mm.


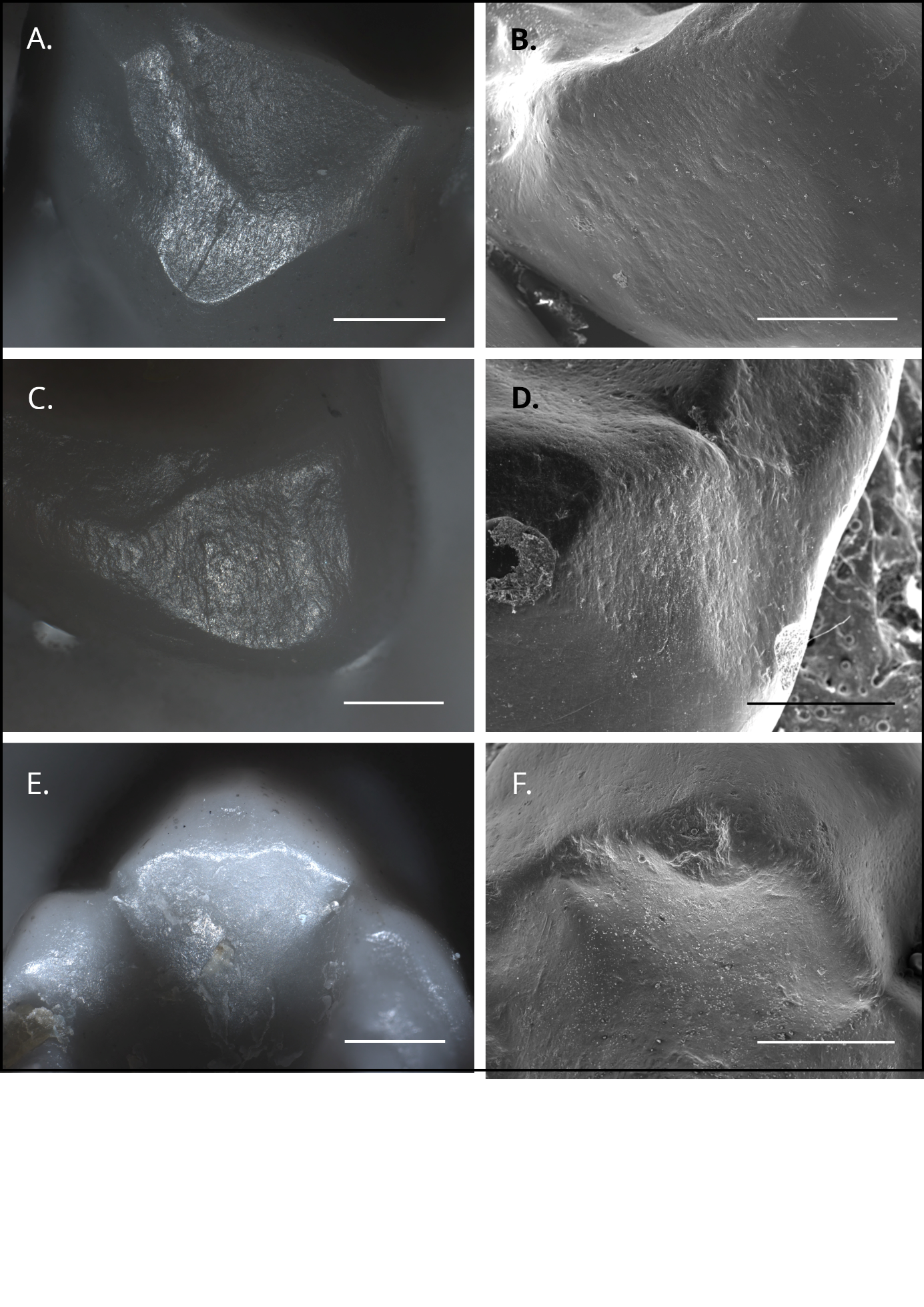


**Figure 11.** Detailed pictures of (A, B) facet M2-PR-ml, (C, D) facet M2-PR-dl and (E, F) facet M2-PR-b using (A, C, E) a digital microscope and (B, D, F) a scanning electron microscope. The specimens of which the pictures were taken are (A) ZFMK-MAM-2017.0575 sin, (B) ZFMK-MAM-2016.0936 sin, (C) ZFMK-MAM-2016.0945 dex, (D) ZFMK-MAM-2016.0939 dex, (E) ZFMK-MAM-2016.0966 sin and (F) ZFMK-MAM-2016.0932 dex. The scale bars represent 1 mm.

**Figure 12.** Detailed pictures of (A, B) facet M2-MTCL-mb and (C) facet M2-PRECNG-d using (A) a digital microscope and (B, C) a scanning electron microscope. The specimens of which the pictures were taken are (A)
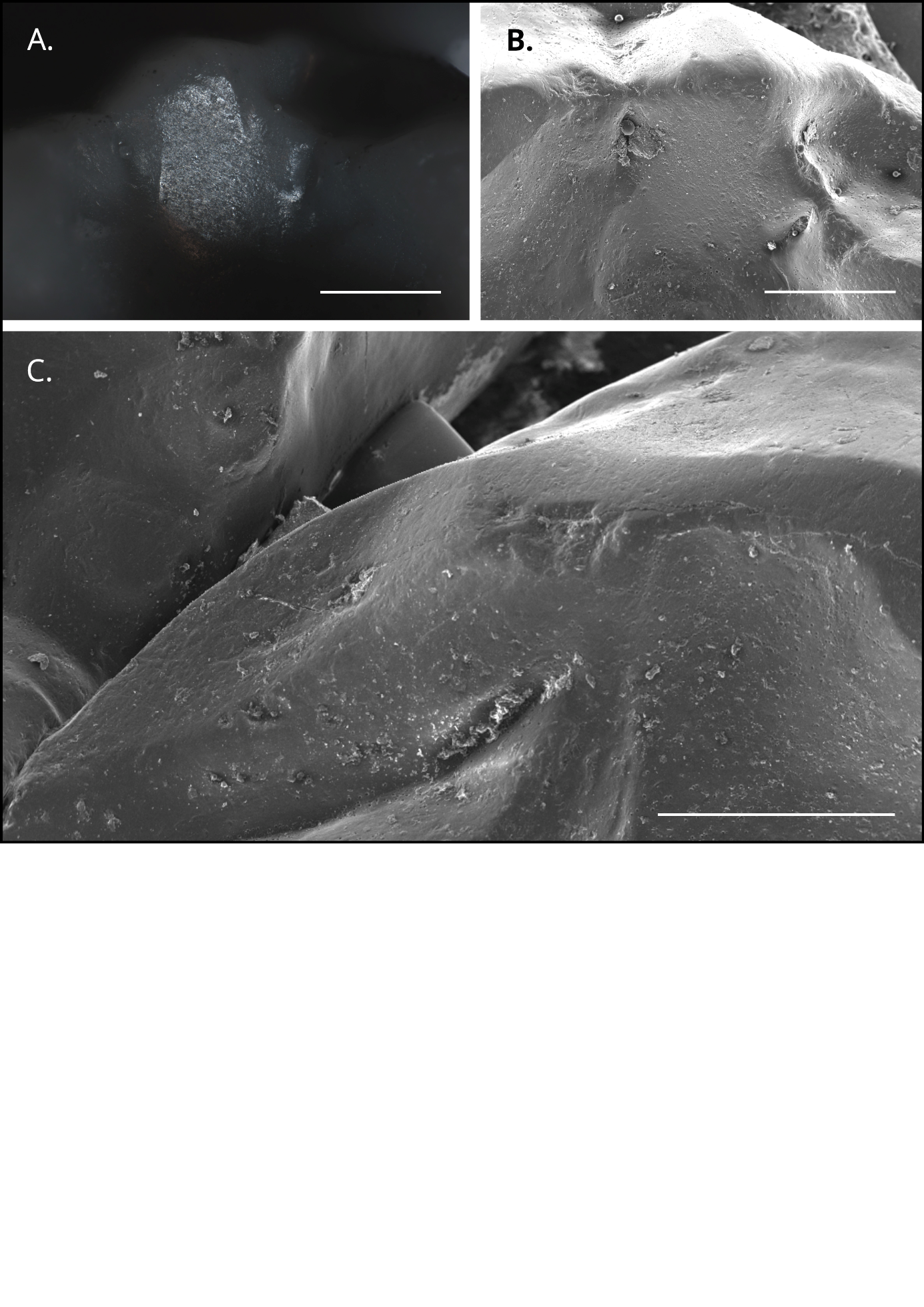
ZFMK-MAM-2016.0968 dex and (B, C) ZFMK-MAM-2016.0986 dex. The scale bars represent 1 mm.


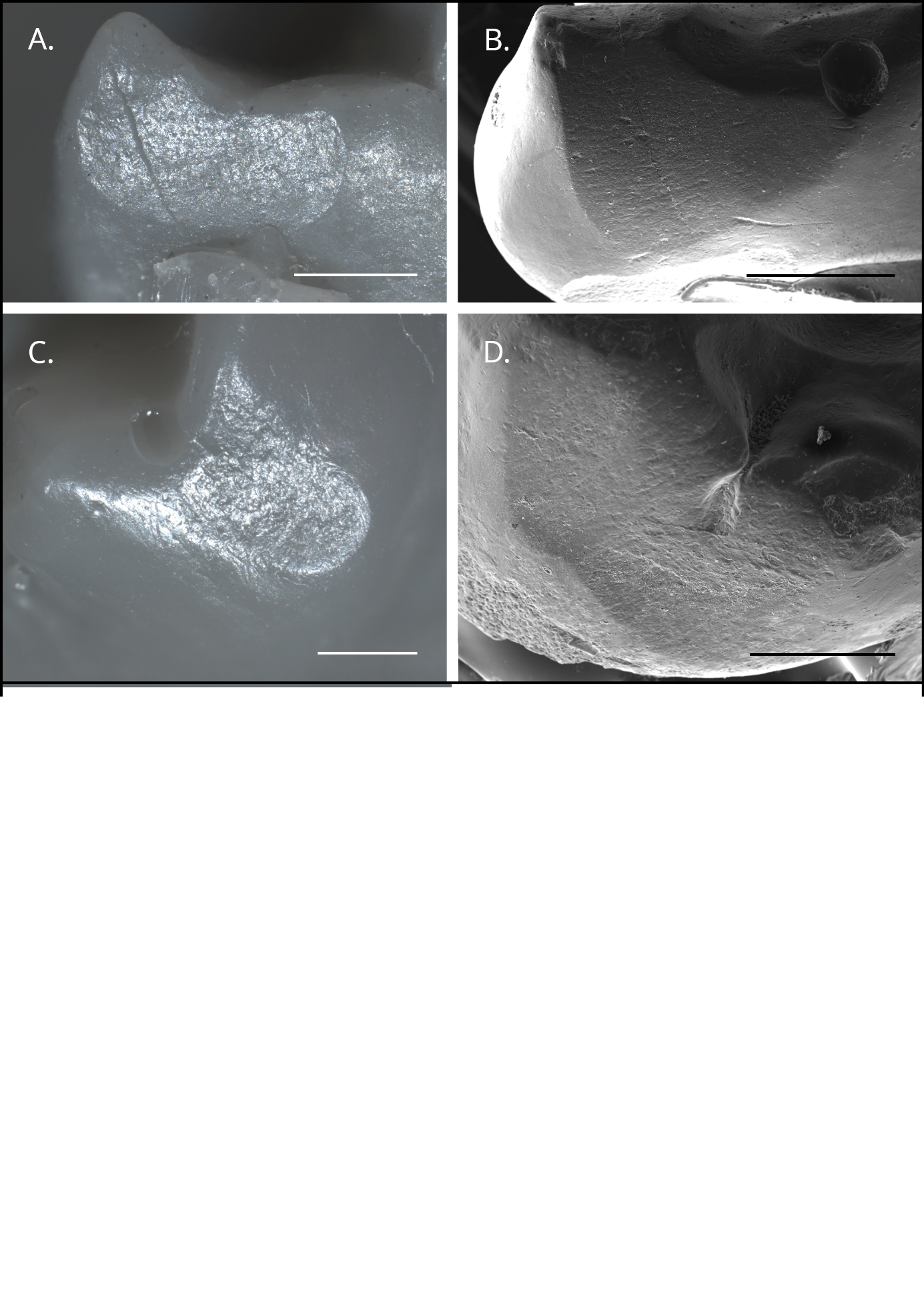


**Figure 13.** Detailed pictures of (A, B) facet m1-pa-m and (C, D) facet m1-pr-mb using (A, C) a digital microscope and (B, D) a scanning electron microscope. The specimens of which the pictures were taken are (A) ZFMK-MAM-2016.0907 sin, (B) ZFMK-MAM-2016.0968 dex, (C) ZFMK-MAM-2016.0945 dex and (D) ZFMK-MAM-2016.0936 dex. The scale bars represent 1 mm.


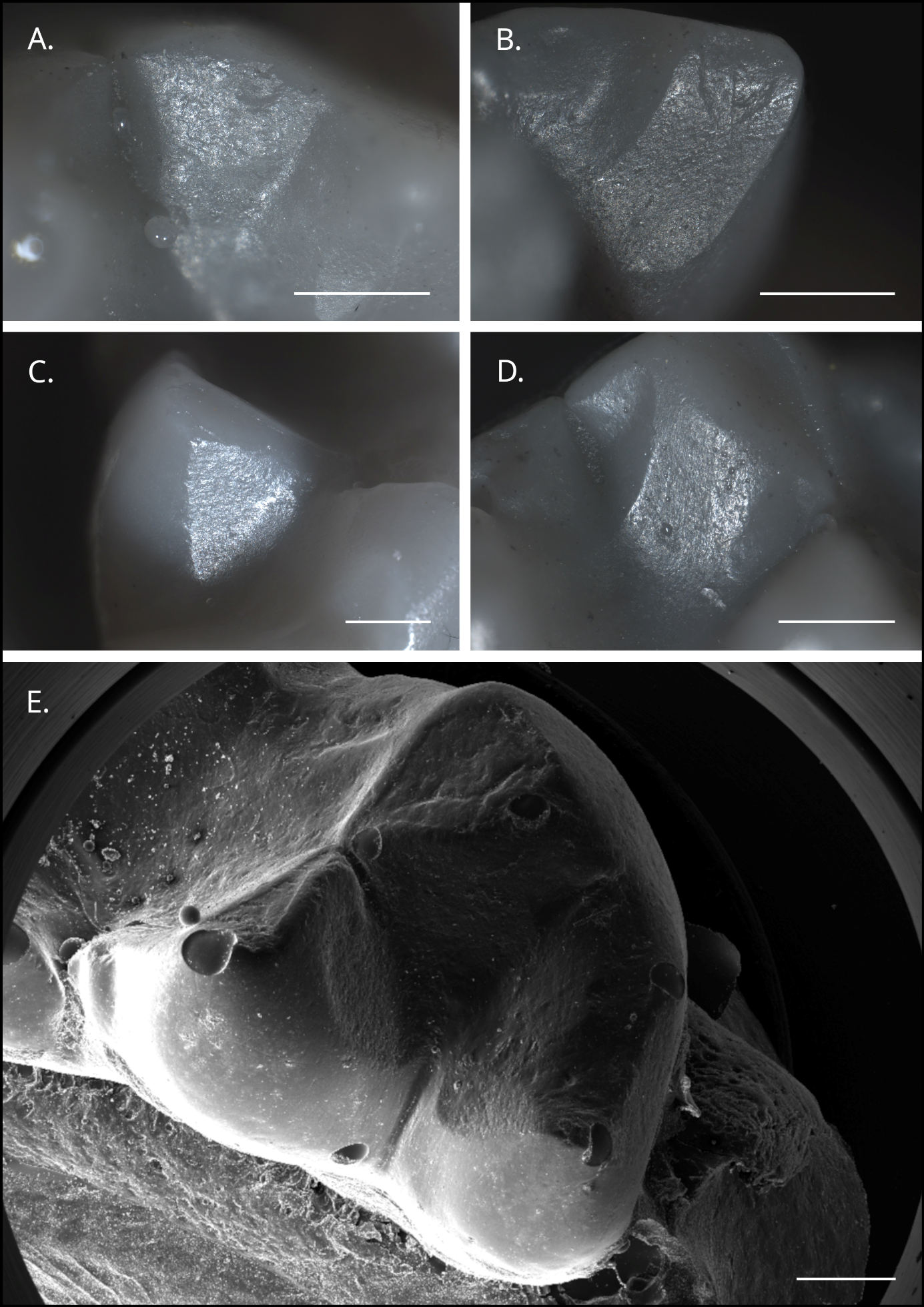


**Figure 14.** Detailed pictures of (A, B) facet m1-pa-d, (C) facet m1-me-mb, (D) facet m1-pr-ml and (E) an overview of the m1 trigonid using (A, B, C, D) a digital microscope and (E) a scanning electron microscope. The specimens of which the pictures were taken are (A) ZFMK-MAM-2016.0907 sin, (B) ZFMK-MAM-2016.0968 sin, (C) ZFMK-MAM-2016.0928 sin, (D) ZFMK-MAM-2016.0936 dex and (E) ZFMK-MAM-2016.0936. The scale bars represent 1 mm.


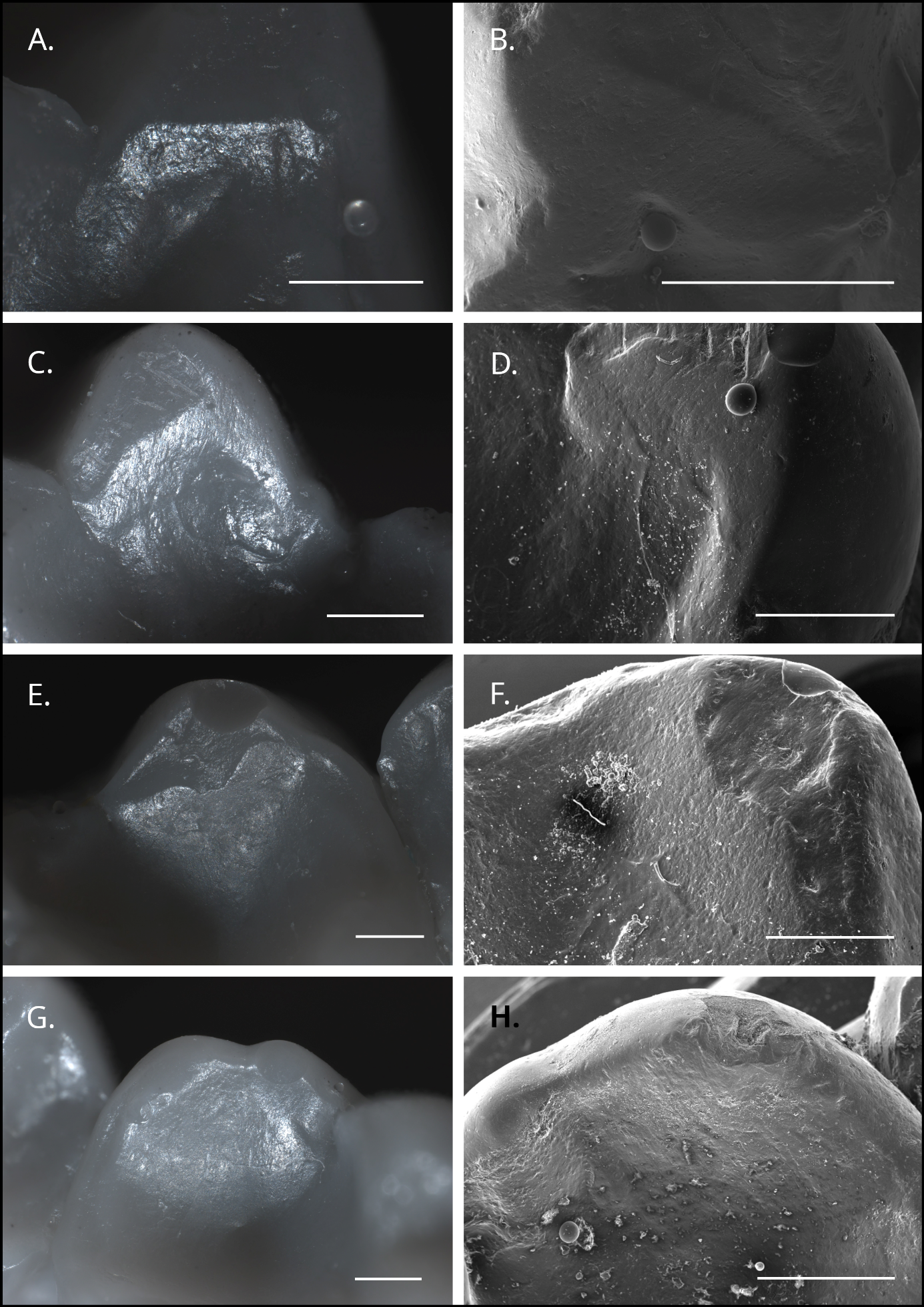


**Figure 15.** Detailed pictures of (A, B) facet m1-pr-d and (C, D) facet m1-me-db, (E, F) facet m1-hy-ml and (G, H) facet m1-en-mb using (A, C, E, G) a digital microscope and (B, D, F, H) a scanning electron microscope. The specimens of which the pictures were taken are (A, E, G) ZFMK-MAM-2016.0945 dex, (B) ZFMK-MAM-2016.0968 dex, (C, D, F) ZFMK-MAM-2016.0939 sin, (H) ZFMK-MAM-2016.0879 dex. The scale bars represent 1 mm.


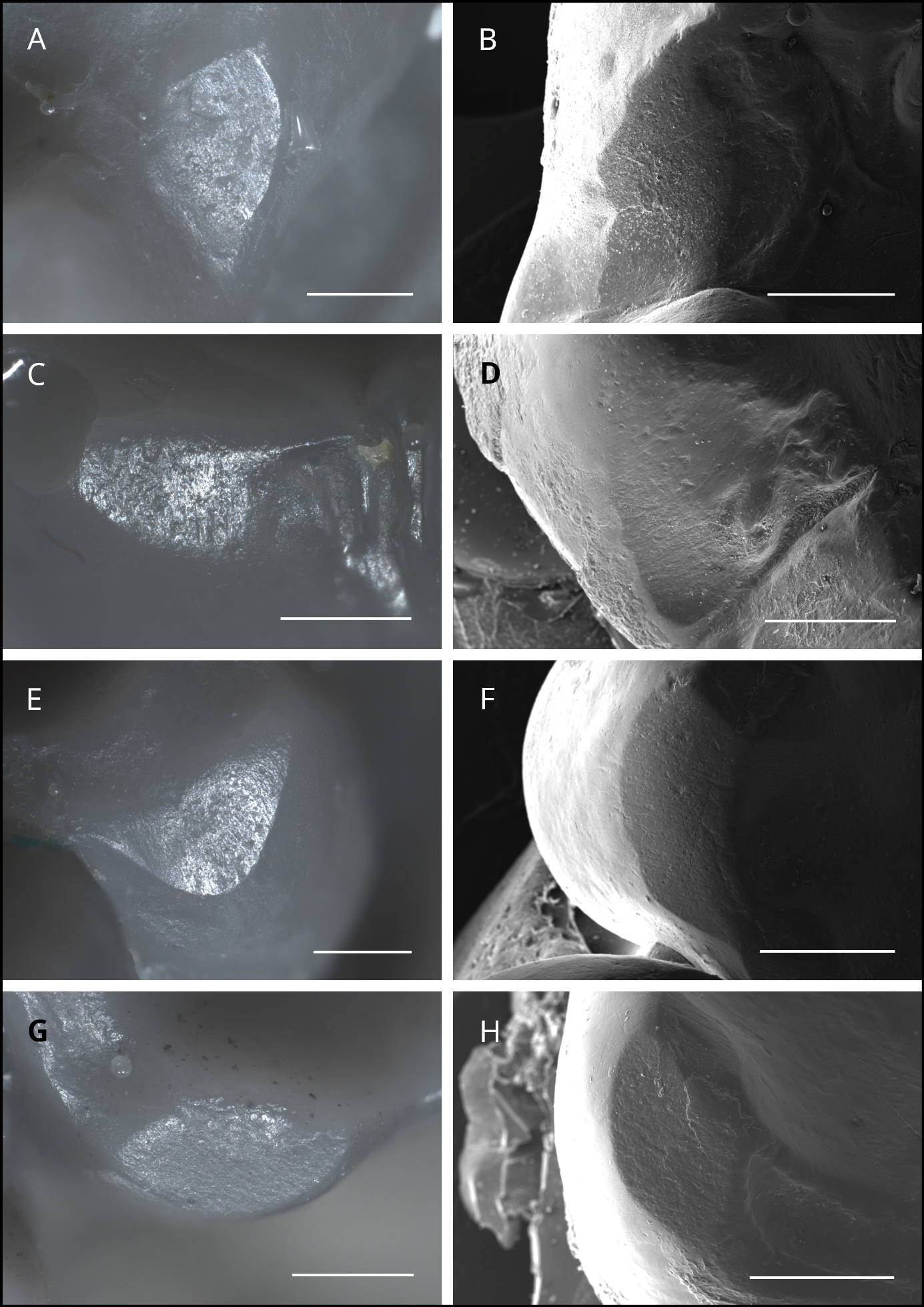


**Figure 16.** Detailed pictures of (A, B) facet m1-msc-db and (C, D) facet m1-hy-mb, (E, F) facet m1-hy-db and (G, H) facet m1-en-dl using (A, C, E, G) a digital microscope and (B, D, F, H) a scanning electron microscope. The specimens of which the pictures were taken are (A, E) ZFMK-MAM-2017.0578 dex, (B, F) ZFMK-MAM-2016.0968 dex, (C) ZFMK-MAM-2016.0945 dex, (D, H) ZFMK-MAM-2016.0936 dex and (G) ZFMK-MAM-2016.0936 sin . The scale bars represent 1 mm.


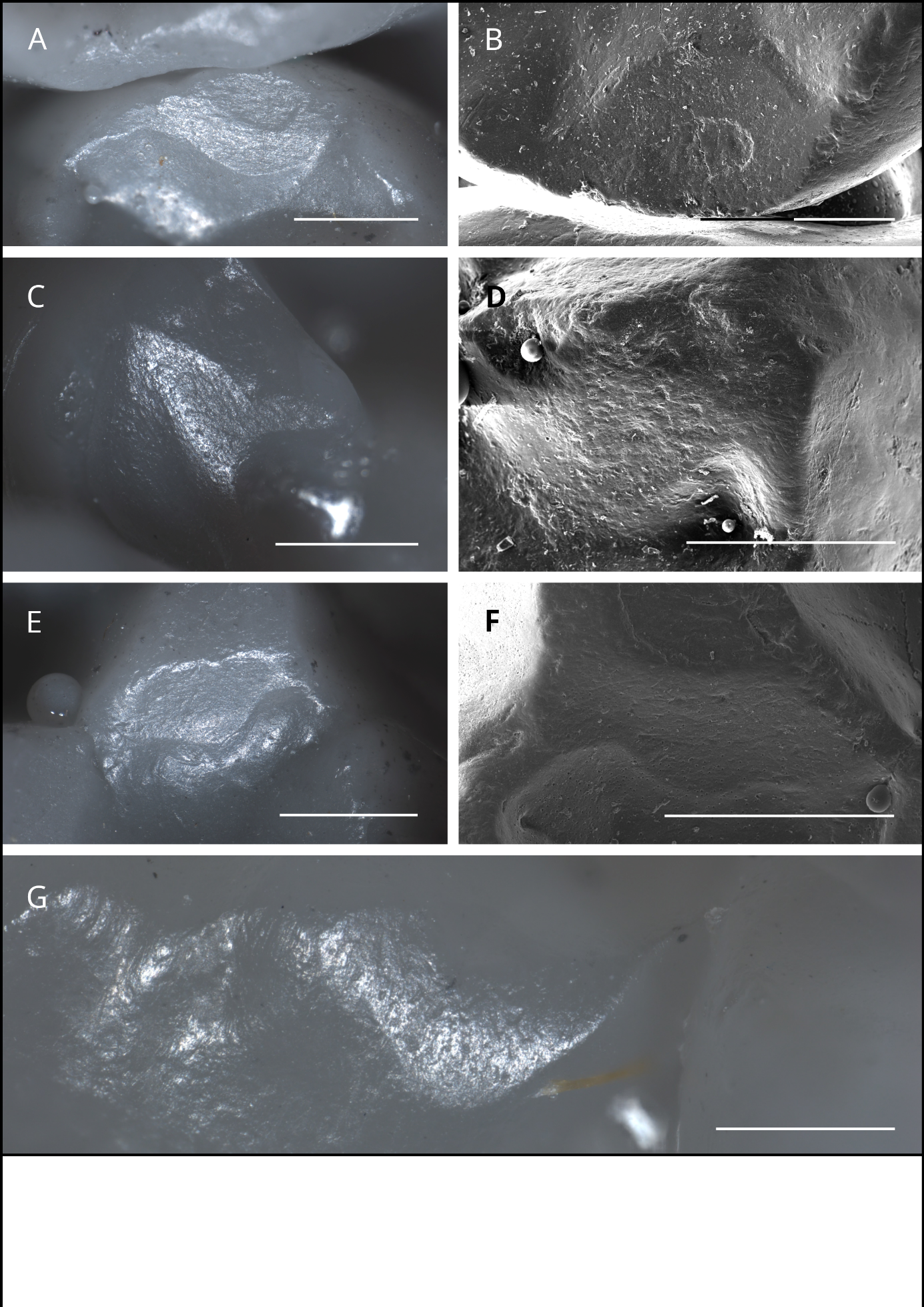


**Figure 17.** Detailed pictures of (A, B) facet m2-pacr-d and (C, D) facet m2-pr-ml, (E, F) facet m2-pr-dl and (G) facet m2-pr-mb using (A, C, E, G) a digital microscope and (B, D, F) a scanning electron microscope. The specimens of which the pictures were taken are (A, B, E, F) ZFMK-MAM-2016.0968 dex, (C, D) ZFMK-MAM-2016.0897 dex and (G) ZFMK-MAM-2016.0894 dex. The scale bars represent 1 mm.


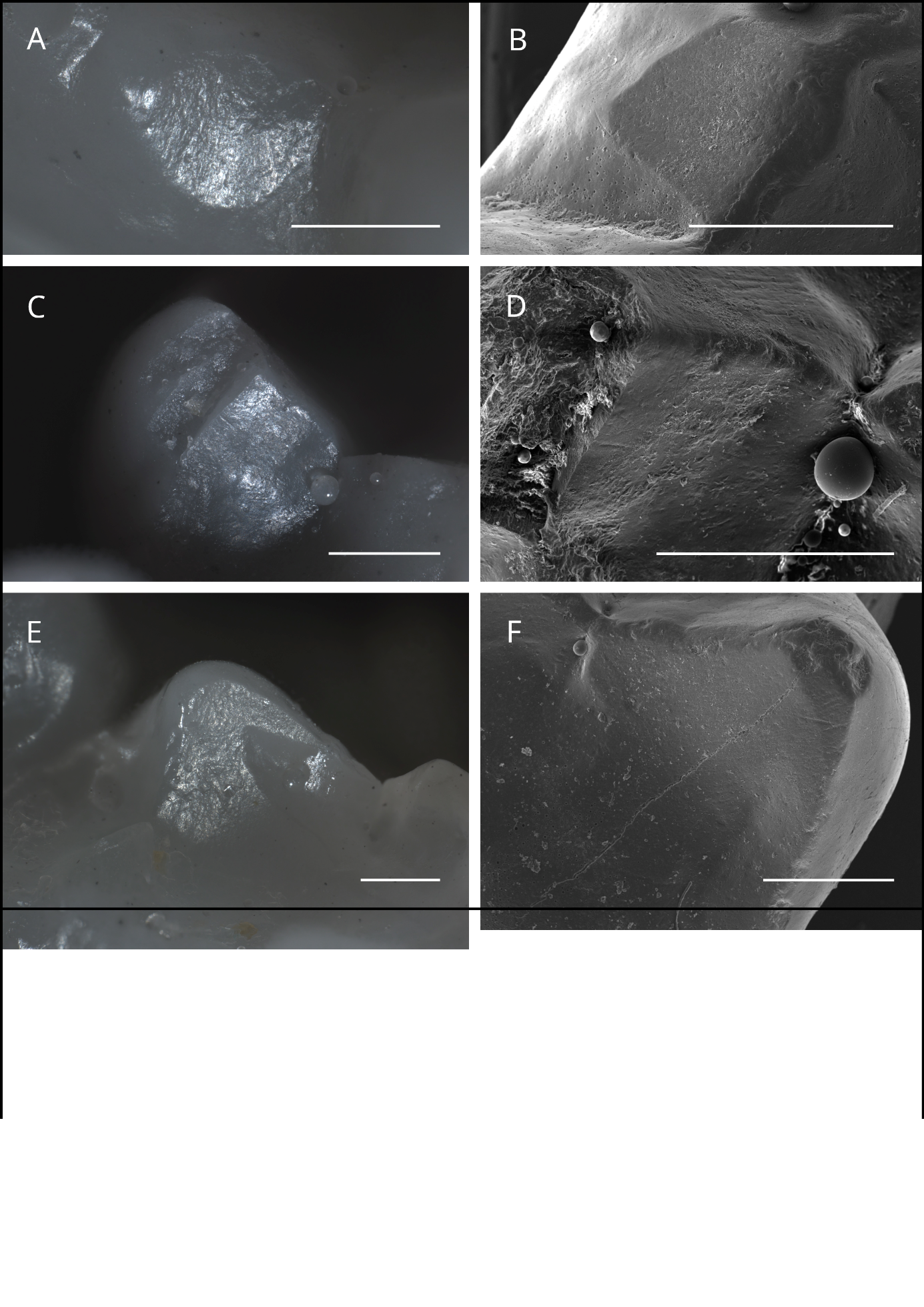


**Figure 18.** Detailed pictures of (A, B) facet m2-pr-db and (C, D) facet m2-me-mb and (E, F) facet m2-me-db using (A, C, E) a digital microscope and (B, D, F) a scanning electron microscope. The specimens of which the pictures were taken are (A) ZFMK-MAM-2016.0939 sin, (B, F) ZFMK-MAM-2016.0968 dex, (C) ZFMK-MAM-2016.0879 sin, (D) ZFMK-MAM-2016.0879 dex and (E) ZFMK-MAM-2016.0936 sin. The scale bars represent 1 mm.


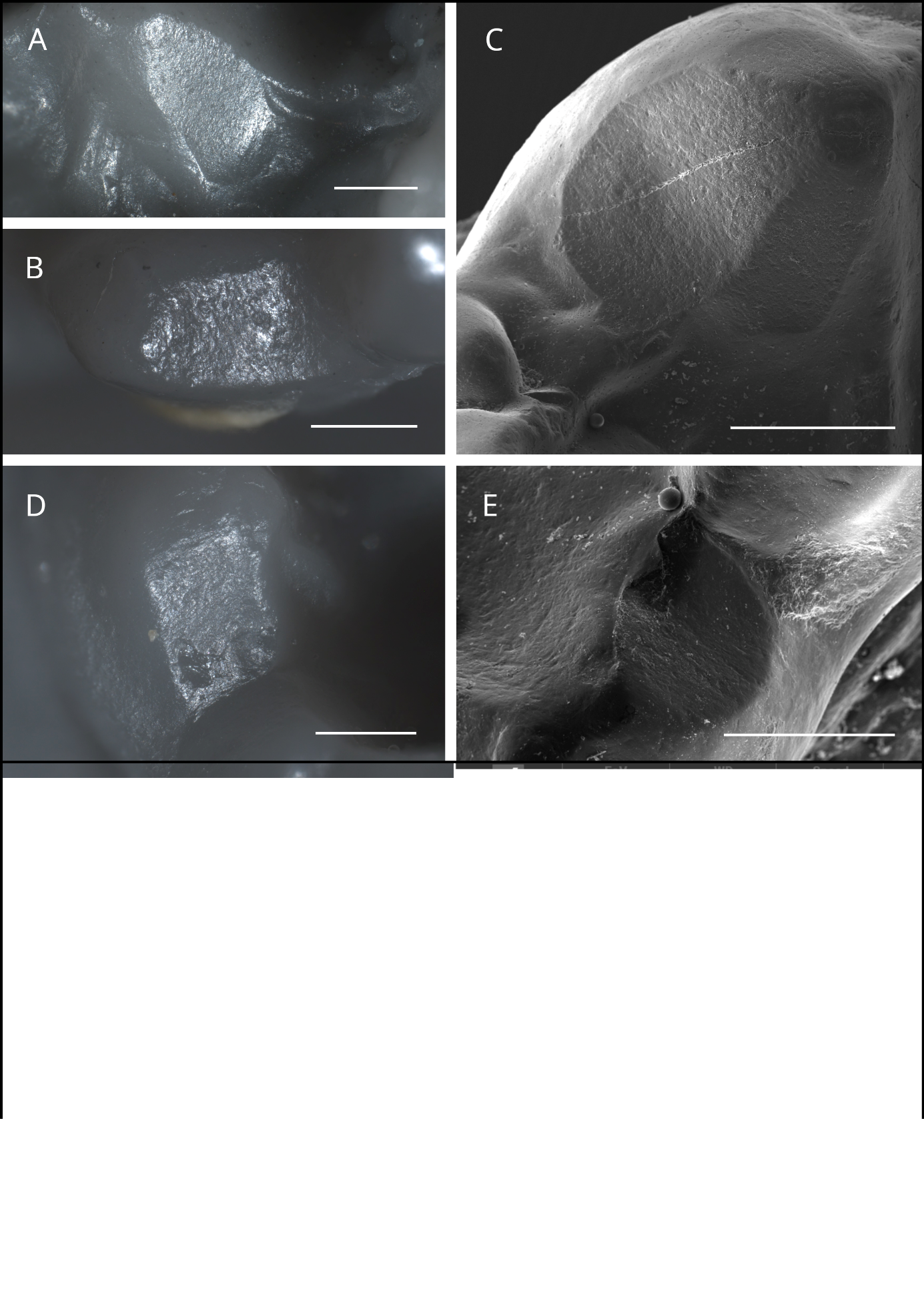


**Figure 19.** Detailed pictures of (A) facet m2-hy-dl and (B) facet m2-hy-db, (C) an overview of facets m2-hy-dl and m2-hy-db and (D, E) facet m2-hy-mb using (A, B, D) a digital microscope and (C, E) a scanning electron microscope. The specimens of which the pictures were taken are (A, C) ZFMK-MAM-2016.0968 dex, (B, E) ZFMK-MAM-2016.0939 sin and (D) ZFMK-MAM-2016.0932 dex. The scale bars represent 1 mm.


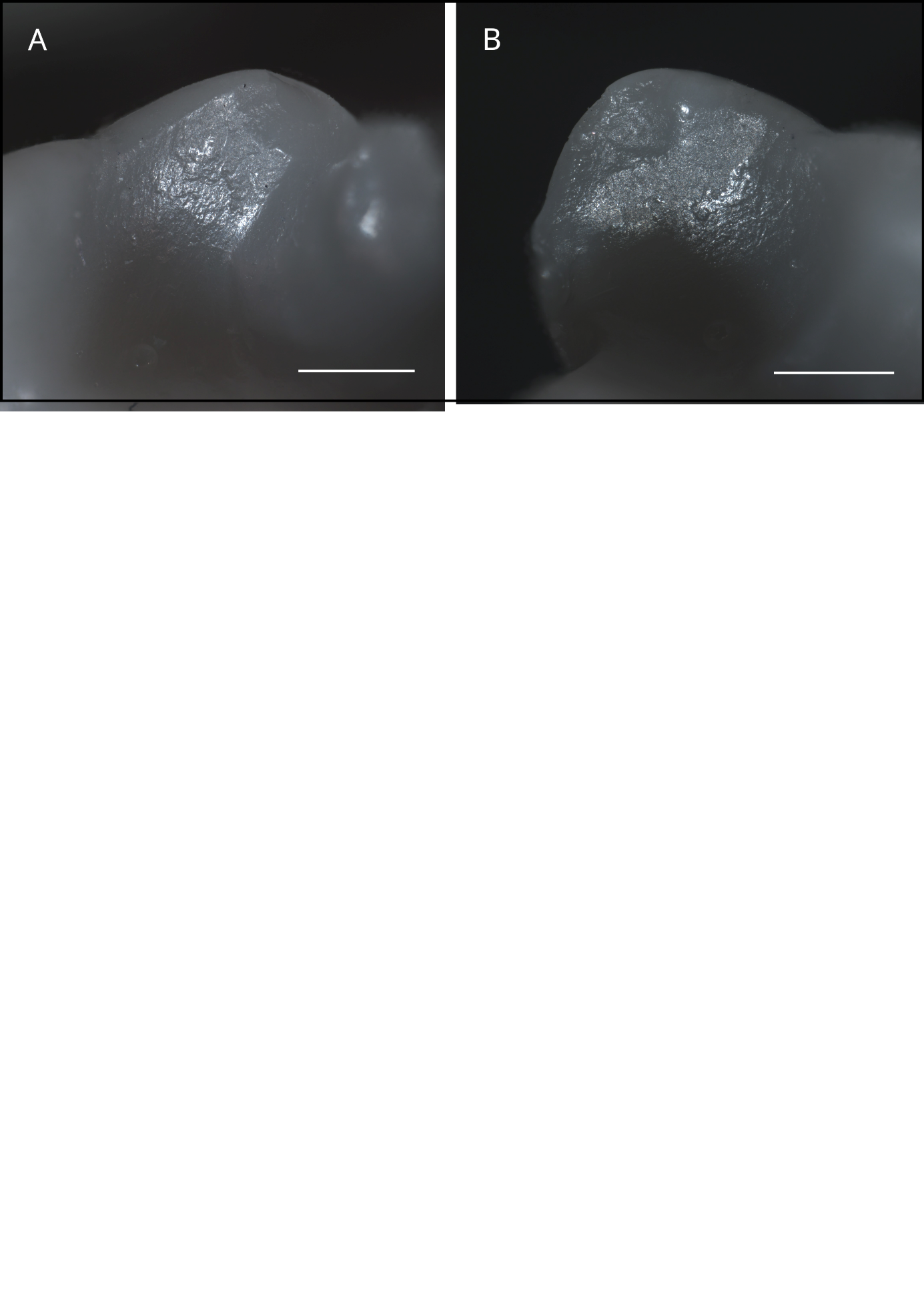


**Figure 20.** Detailed pictures of (A) facet m2-en-db and (B) facet m2-hld-m using a digital microscope. The specimen of which the pictures were taken is ZFMK-MAM-2016.0897 dex. The scale bars represent 1 mm.
